# Integration of Transcriptomic and Proteomic Profiles Reveals Multiple Levels of Genetic Regulation of Taproot Growth in Sugar Beet (*Beta vulgaris* L.)

**DOI:** 10.3389/fpls.2022.882753

**Published:** 2022-07-13

**Authors:** Ningning Li, Yongfeng Zhang, Xuefeng Wang, Huailong Ma, Yaqing Sun, Guolong Li, Shaoying Zhang

**Affiliations:** Sugar Beet Physiological Research Institute, Inner Mongolia Agricultural University, Hohhot, China

**Keywords:** sugar beet, taproot growth and development, proteomics, transcriptomics, xyloglucan endotransglucosylase/hydrolase

## Abstract

Sugar beet taproot growth and development is a complex biological process involving morphogenesis and dry matter accumulation. However, the molecular regulatory mechanisms underlying taproot growth and development remain elusive. We performed a correlation analysis of the proteome and transcriptome in two cultivars (SD13829 and BS02) at the start and the highest points of the taproot growth rate. The corresponding correlation coefficients were 0.6189, 0.7714, 0.6803, and 0.7056 in four comparison groups. A total of 621 genes were regulated at both transcriptional and translational levels, including 190, 71, 140, and 220 in the BS59-VS-BS82, BS59-VS-SD59, BS82-VS-SD82, and SD59-VS-SD82 groups, respectively. Ten, 32, and 68 correlated-DEGs-DEPs (cor-DEGs-DEPs) were significantly enrdiched in the proteome and transcriptome of the BS59-VS-BS82, SD59-VS-SD82, and BS82-VS-SD82 groups, respectively, which included ribonuclease 1-like protein, DEAD-box ATP-dependent RNA helicase, TolB protein, heat shock protein 83, 20 kDa chaperonin, polygalacturonase, endochitinase, brassinolide and gibberellin receptors (BRI1 and GID1), and xyloglucan endotransglucosylase/hydrolase (XTH). In addition, *Beta vulgaris* XTH could enhance the growth and development of Arabidopsis primary roots by improving cell growth in the root tip elongation zone. These findings suggested that taproot growth and expansion might be regulated at transcriptional and posttranscriptional levels and also may be attributed to cell wall metabolism to improve cell wall loosening and elongation.

## Introduction

Sugar beet (*Beta vulgaris* L.), a crucial industrial crop for sugar production, provides ~30% of the world's annual sugar production. It is also an important raw material in the production of animal feed and bioethanol (Liu et al., 2008). The fleshy taproot is the main harvest portion of the beet and is rich in carbohydrates, amino acids, and secondary metabolites. Traditional breeding of sugar beet has aimed to increase the taproot yield (Dohm et al., 2014). The taproot growth and development of sugar beet is a complex biological process involving the vascular bundle formation, cell division and expansion, and dry matter accumulation. Therefore, understanding the regulatory mechanisms underlying taproot growth will allow for new sugar beet cultivars with high yield and quality to be engineered.

To date, gene regulation in root development has been studied in several plant species, such as *Arabidopsis thaliana* (Zhou et al., 2011; Petricka et al., 2012; Smith and De Smet, 2012), *Zea mays* (Taramino et al., 2007), and *Oryza sativa* (Ge and Wang, 2012). However, the taproot of sugar beet is a storage root, and the gene regulation and molecular mechanisms of storage root development are largely unknown. Recently, the thickening mechanism of taproot has been deeply studied. The main cortex splitting is an important sign for the initiation of taproot growth in plants, and the thickening taproot is comprised of the hypocotyl and root axis and is mainly driven by parenchyma cell division and subsequent cell expansion in the cambium, which produces a substantial core of secondary xylem and a slightly broader secondary phloem (Tsuro et al., 2008). Some physiological studies have shown that the hormone metabolism may be involved in regulating thickening of taproot in radish, such as gibberellic acid, abscisic acid, indole acetic acid, and cytokinin (Matveeva et al., 2004; Choi et al., 2011; Jung and McCouch, 2013). In addition, some genes involved in regulating storage root formation have been identified, such as the receptor-like kinase gene was mainly observed in the primary cambium and meristems of the xylem and plays an important role in dividing cells and thickening of taproot (You et al., 2003; Tanaka et al., 2005). MADS-box1 gene can induce cytokinin and jasmonic acid to promote the taproot growth and development (Ku et al., 2008). Recently, radish root transcriptomic studies have shown that many differentially expressed genes (DEGs), (MADS-box, XTH16, EXPA9, CalS CaM, cyclin, and syntaxin) play important roles in many metabolic processes related to taproot thickening, including the cell events, plant hormone metabolism, cell wall modification, and signal transduction and metabolism (Yu et al., 2015, 2016). However, the molecular mechanism of sugar beet taproot thickening remains elusive.

The RNA-seq technology, an important tool for precise transcriptomic analysis (Wang et al., 2010), can identify DEGs in different tissues, organs, or developmental stages in plants (Li et al., 2011; Cheng et al., 2013; Park et al., 2014). Using this approach, the roles of DEGs have been explored in root development in maize (Li et al., 2011), cucumber (Zhang et al., 2014a), Brassica (Zhang et al., 2014b), and lotus (Cheng et al., 2013). Moreover, isobaric tags for relative and absolute quantification (iTRAQ), a robust mass spectrometry (MS) technique, can better understand the differences in the accumulation of protein, which has been applied to study adventitious root development mechanisms in mulberry (Ross et al., 2004; Tang et al., 2016) and apple (Lei et al., 2018). The correlation analysis between proteomics and transcriptomics can better clarify the molecular mechanism of plant tolerance to abiotic and biotic stresses, such as the *Gossypium hirsutum* response to salt stress (Peng et al., 2018), light-induced anthocyanin biosynthesis in *Solanum melongena* (Li et al., 2017), and the *Morus atropurpurea* fruit response to *Ciboria carunculoides* (Dai et al., 2019). However, no studies of taproot growth regulation and thickening have been conducted in sugar beet using combined transcriptome and proteomic analysis. Previously, we performed RNA-seq and comparative analyses in the high-yield cultivar SD13829 (SD) and the low-yield cultivar BS02 (BS) at five growth stages and found that many Gene Ontology (GO) terms, such as cell wall, cytoskeleton, and enzyme-linked receptor protein signaling pathway, were enriched at the highest growth rate stage (82 days after emergence [DAE]) in both cultivar (Zhang et al., 2017). In this study, the two cultivars (SD and BS) at two time points of taproot growth rate (start point: 59 DAE; highest point 82 DAE) were performed proteomic sequencing using iTRAQ and combined with transcriptome data for correlation analysis to further investigate the mechanism of beet taproot growth and development. This study will provide insight into the molecular mechanism of taproot growth and development and also will provide a theoretical basis for cultivating high-yield sugar beet varieties.

## Materials and Methods

### Plant Materials and Treatments

Two cultivars of the sugar beet (*Beta vulgaris*) were used for this study and were described by Zhang et al. (2017). In brief, two representative cultivars (SD13829 and BS02) were obtained by the screening of numerous cultivars. The SD13829, a mono-germy diploid cultivar with high yield, was purchased from Strube GmbH & Co. KG (Solingen, Germany). The taproot of SD13829 exhibited a high growth rate and high fresh weight, but low sucrose content. The BS02 cultivar, a pluri-germy diploid cultivar with low yield, was bred by the Sugar Beet Physiological Research Institute, Inner Mongolia Agricultural University, China. The taproot of BS02 showed a high sucrose content, but low growth rate and fresh weight. Both of the cultivars were grown on the farm of Inner Mongolia Agricultural University with the plant spacing of 25 cm and row spacing of 50 cm, and we had obtained permission from the farmer to collect plant samples. The sampling strategy was based on our previous results; the time point of beginning rapid growth (59 DAE) and the highest growth rate of taproot (82 DAE) were collected in both cultivars. Nine taproots (three biological replicates) at 59 and 82 DAE were collected and frozen in liquid nitrogen for further analysis.

*Arabidopsis thaliana* seeds of the Columbia-0 (Col-0) genotype were surface-sterilized with a solution of 2% sodium hypochlorite and 0.5% Tween 20 and then sown on MS medium. After vernalization at 4°C for 3 d, the seeds were cultured at 22°C under a photoperiod of 16-h light/8-h dark and a photon flux density of 45 μmol m^−2^ s^−1^.

### Protein Preparation

The plant tissues were broken in lysis buffer (with enzyme inhibitors) using the tissue lyser machine and centrifuged at 25,000 × g for 20 min. Five volumes of cold acetone was added to the recovered supernatant and stored at−20°C for 2 h. After centrifuging again, the pellets were dissolved with lysis buffer, and the 10 mM DTT was added into the solution at 56°C for 1 h to disrupt disulfide bonds. The solution was mixed with 55 mM IAM with the dark treatment for 45 min. After five volumes of cold acetone mixture and centrifugation, the pellet was dissolved in lysis buffer and the supernatant was stored at −80°C for further analysis.

### iTRAQ Labeling and Liquid Chromatography–Electrospray Ionization Tandem Mass Spectrometry Analysis

Total proteins were digested by Trypsin Gold (Promega, Madison, WI, USA) with a ratio of 20:1 at 37°C for 12 h. The digested proteins were labeled with the iTRAQ tags using the eight-plex iTRAQ reagent (Applied Biosystems, Foster City, CA, USA) according to the manufacturer's protocol, and the lyophilized labeled peptides were resuspended with the solution A (95% H_2_O, 5% acetonitrile [ACN]; pH 9.8) and loaded onto Gemini C18 column (4.6 × 250 mm). The peptide fractions were eluted with buffer B (5% H_2_O, 95% ACN; pH 9.8) at a flow rate of 1 mL/min. The elutions have monitored the absorbance at 214 nm and collected 20 fractions and lyophilized. The fraction samples were performed by the LC-ESI-MS/MS analysis as described previously (Li et al., 2017). Briefly, the data acquisition was performed using the Triple TOF 5600 System (AB SCIEX, Concord, ON, Canada) fitted with a Nanospray III source (AB SCIEX) at the conditions of 15 psi nebulizer gas, 30 psi curtain gas, 2.5 kV ion spray voltage, and an interface heater temperature of 150°C. The total cycle time was fixed to 3.3 s, the Q2 transmission window for 100 Da was 100%, and the dynamic exclusion was set for 1/2 of peak width (15 s).

### iTRAQ Data Analysis

MGF file was obtained from raw data through Proteome Discoverer 1.2 (Thermo Fisher Scientific, Bremen, Germany) software analysis, and the protein identification used the Mascot software (Matrix Science, London, UK; version 2.3.02) against the database of sugar beet genome. The allowable mass tolerance of the intact peptide mass and fragmented ions were 0.1 and 0.05 Da (ppm) in protein identification, respectively, and a missed cleavage was allowed in the trypsin digest. The variable modifications were the Gln- > pyro-Glu (N-term Q), deamidated (NQ), and oxidation (M), and the fixed modifications were the iTRAQ8plex (K), carbamidomethyl (C), and iTRAQ8plex (N-term). The +2 and +3 were the charge states of peptides. The automatic decoy database search was carried out using the Mascot software. The identified peptides have 95% significance confidence interval to reduce the possibility of false peptide identification. All proteins in the proteome and all genes in the transcriptome were compared using BLAST reciprocal best hit analysis (Altschul et al., 1990). DEGs were screened according to the |log2 ratio| ≥ 1 (*P* < 0.001) and false discovery rate ≤ 0.001. Differentially expressed proteins (DEPs) were screened by the fold change of proteins ≥ 1.5 (*P* < 0.05). The shared DEGs and DEPs were identified from proteome and transcriptome data. Functional annotations of the DEPs or DEGs were conducted using the Blast2GO program and the non-redundant protein database (NR; NCBI). *P* ≤ 0.05 was used to confirm the significance of the GO, KEGG pathway, and MapMan analysis results.

### Genetic Transformation of Arabidopsis

The *BvXTH8* open reading frame was digested from the pBoLn-T-BvXTH vector with SacI and SalI and subcloned into the SacI/SalI site of the pCAMBIA1300 plasmid to construct pCAMBIA1300-BvXTH8, which was used for transformation of *A. thaliana* through Agrobacterium-mediated inflorescence infiltration method (Kim et al., 1999). Transgenic homozygous lines were prepared by hygromycin resistance screening and self-pollination. The homozygous transgenic plants were detected by genomic PCR and real-time RT-PCR. The genomic PCR was performed using the following primers: XTH-F1 (CCCTATATGGCTTCCTCCTC) and XTH-R1 (CAGCAAAACTGCAGTCAGAGT). Real-time RT-PCR was performed using the following primers: XTH-F2 (GTCAGCGGTCAACCATACAC) and XTH-R2 (ACCTTGTGTTGCCCAATCAT). β-actin was used as an internal control.

### XTH Enzyme Activity Assay

The XTH enzyme was obtained as previously reported (Sulova et al., 1995). Briefly, 2 g samples were ground in liquid nitrogen, 1 mL of the 10 mM sodium phosphate buffer (pH 7.0) was added, and the mixture was centrifuged at 10,000 × g for 25 min at 6°C. The precipitate was washed twice with 10 mM sodium phosphate solution (pH 7.0) and then centrifuged at 6°C 10,000 × g for 18 min. The pellet was resuspended in 10 mM sodium phosphate solution at 4°C for 24 h and then centrifuged at 4°C 10,000 × g for 15 min. The supernatant was stored at 4°C and used to detect enzyme activity.

XTH activity was assayed in a reaction solution containing 50 μL of 2 mg/mL xyloglucan, 50 μL of 2 mg/mL xyloglucan oligosaccharides, 50 μL of 400 mM sodium citrate, and 50 μL of the XTH enzyme solution. XTH activity was expressed as the difference measured in the absence and presence of the XTH enzyme solution. After incubating at 37°C for 30 min, 1 M sodium hydroxide was added to terminate the reaction. A freshly prepared solution of 200 μL KI/I2 and 800 μL 20% Na_2_SO_4_ was then added to the reaction, left in a dark room for 30 min, and colorimetrically examined at 620 nm (UV752N; INESA, Shanghai, China). For the blank, 50 μL of sodium phosphate solution was used instead of 50 μL XTH enzyme solution.

### Phenotypic Determination and Microscopic Observation of Transgenic Arabidopsis

Transgenic Arabidopsis seeds were grown on MS medium, and the root length and seedling fresh weight were compared at 2 weeks after seed germination. Ten plants were measured for each transgenic line. The root tips of 10-day-old transgenic seedlings were used for microscopic observation. A 2-mm-long sample from the root tip was used for temporary loading. The cells were observed microscopically adjacent to the elongation zone, and the area of all clearly contoured cells in the range of 2 mm was determined. Five biological replicates were measured for each sample.

## Results

### Overview of the Transcriptomic and Proteomic Analysis

**Figure 1:**
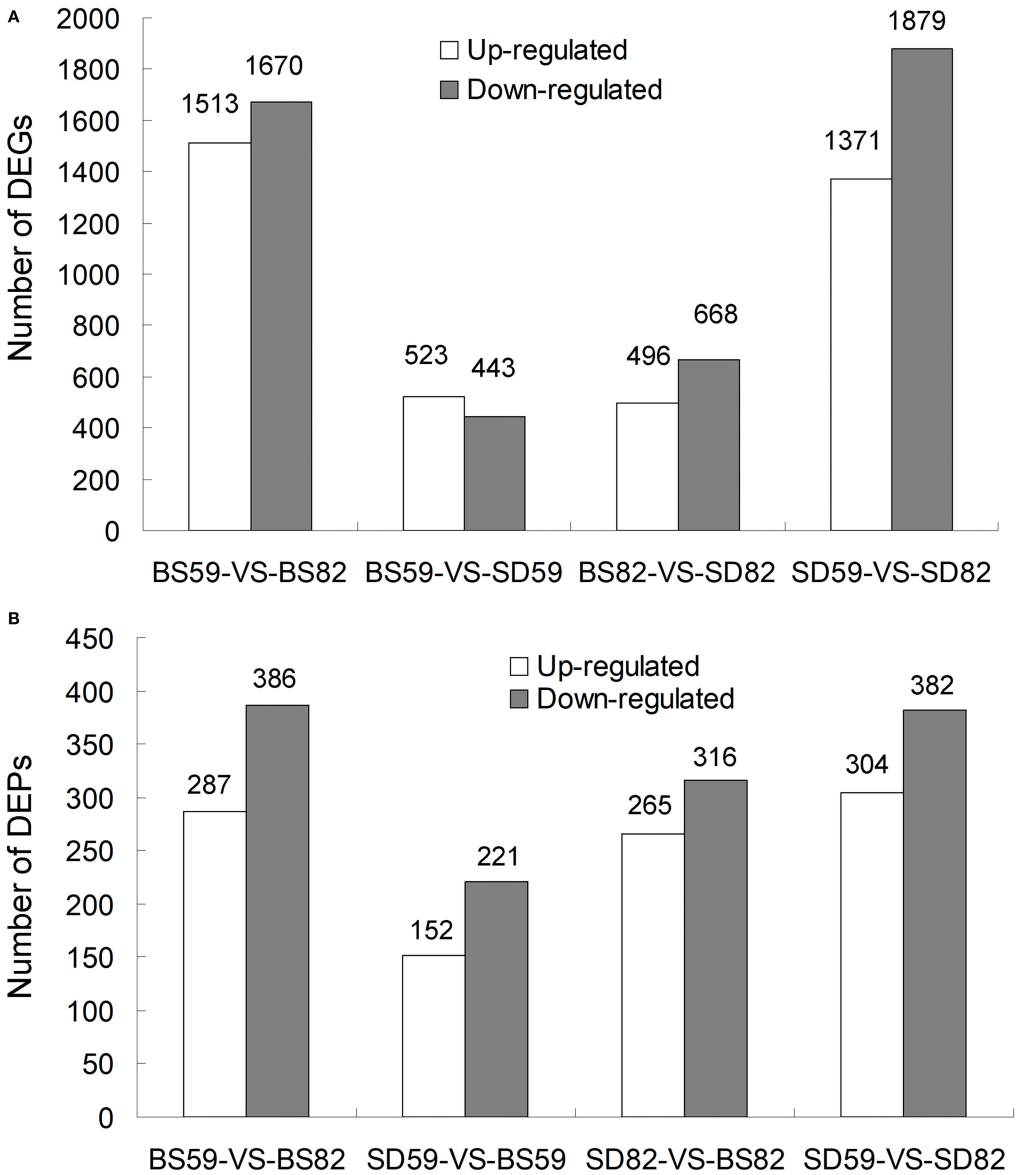
Overview of proteome and transcriptome data analysis. **(A)** Number of DEGs (differentially expressed genes) at two time points of taproot growth rate (59 DAE and 82 DAE) in the BS and SD cultivars. **(B)** Number of DEPs (differentially expressed proteins) at two time points of taproot growth rate in the BS and SD cultivars.

RNA-seq was performed for SD and BS at 59 and 82 DAE. For each sample, 82.23–83.17% of the clean reads were mapped to the genome of *Beta vulgaris* (Dohm et al., 2014), and the uniquely matched clean-read percentages ranged from 70.81% to 71.55% (Supplementary Table 1). DEGs were screened according to the |log2 ratio| ≥ 1 (*P* < 0.001) and false discovery rate ≤ 0.001. A total of 3183 (BS59-VS-BS82), 966 (BS59-VS-SD59), 1164 (BS82-VS-SD82), and 3250 (SD59-VS-SD82) DEGs were found, including 1513, 523, 496, and 1371 upregulated genes and 1670, 443, 668, and 1879 downregulated genes (Figure 1A). Previously, we validated transcriptomic data by qRT-PCR, and the qRT-PCR results were highly consistent with the RNA-seq data, suggesting that the transcriptome data were reliable (Zhang et al., 2017).

The quantitative proteomic analyses were performed for SD and BS at 59 and 82 DAE by iTRAQ platform. A total of 338,982 spectra were generated, 64,267 spectra and 22,197 peptides were matched, and 59,328 unique spectra, 22,197 peptides, and 5,827 proteins were identified with the Q-value ≤ 0.01 (Supplementary Figure 1A). The protein mass distribution, length of peptides, and peptide number distribution were also investigated (Supplementary Figures 1B–D). Differentially expressed proteins (DEPs) were screened by the fold change of proteins ≥1.5 (*P* < 0.05). A total of 673 (BS59-VS-BS82), 373 (BS59-VS-SD59), 581 (BS82-VS-SD82), and 686 (SD59-VS-SD82) DEPs were found, including 287, 152, 265, and 304 upregulated genes and 386, 221, 316, and 382 downregulated genes, respectively (Figure 1B). The more DEPs were found in comparative groups of two growth stages (59 vs. 82), indicating that these DEPs may play important roles in the process of taproot growth and development.

### Correlation Analysis of Transcriptome and Proteome

**Figure 2:**
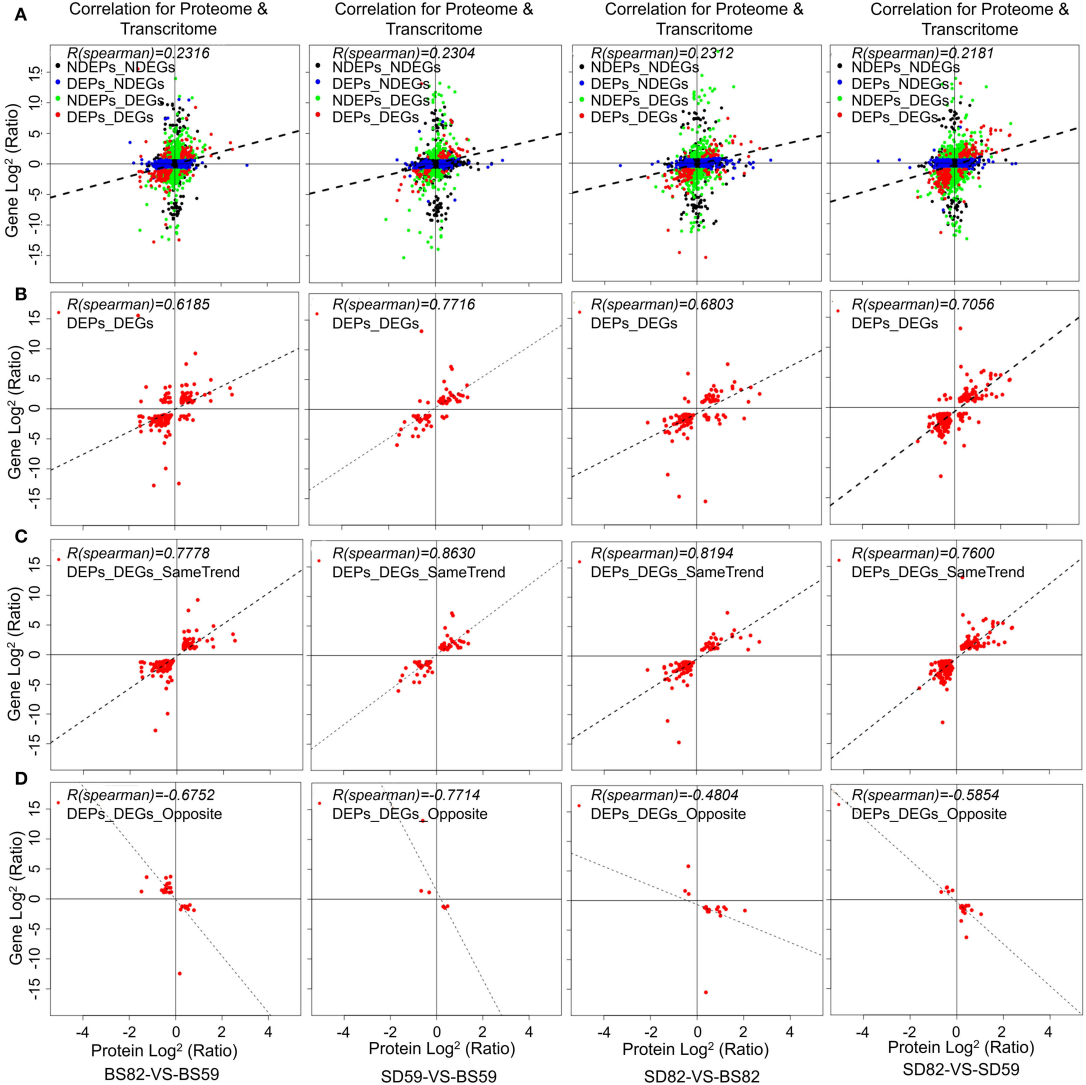
Correlations between protein and mRNA expression. X-axis represents the protein expression level, and Y-axis represents the gene expression level. **(A)** Scatterplots of the relationship between genes quantified in both transcriptomic and proteomic in BS59-VS-BS82, BS59-VS-SD59, BS82-VS-SD82, and SD59-VS-SD82 groups. **(B)** Scatterplots and correlation coefficients between DEGs (differentially expressed genes) and DEPs (differentially expressed proteins). Scatterplots and correlation coefficients between proteins and mRNA expression ratios which are the same **(C)** or opposite **(D)** changing tendency. The black plot indicates none DEPs and DEGs; blue plot indicates DEPs but none DEGs; green plot indicates DEGs but non DEPs; red plot indicates DEPs and DEGs, and all data were log2-transformed.

The correlation analysis of iTRAQ and RNA-seq data was performed, and the correlation coefficient was calculated in four comparison groups (BS59-VS-BS82, BS59-VS-SD59, BS82-VS-SD82, and SD59-VS-SD82; Supplementary Figure 2). The expression levels of proteins and their corresponding transcripts in four comparison groups exhibited lower correlation (r = 0.2316, 0.2304, 0.2312, and 0.2181; Figure 2A), but the DEPs and DEGs showed a higher correlation (r = 0.6189, 0.7714, 0.6803, and 0.7056; Figure 2B). The same or opposite trend correlation coefficients of the DEPs and DEGs also exhibited higher positive or negative correlation (Figures 2C,D). In addition, the lower correlations were observed between DEPs and their corresponding non-additive DEGs, non-additive DEPs and their corresponding DEGs, or non-additive DEPs and their corresponding non-additive DEGs (Supplementary Figure 3).

**Figure 3:**
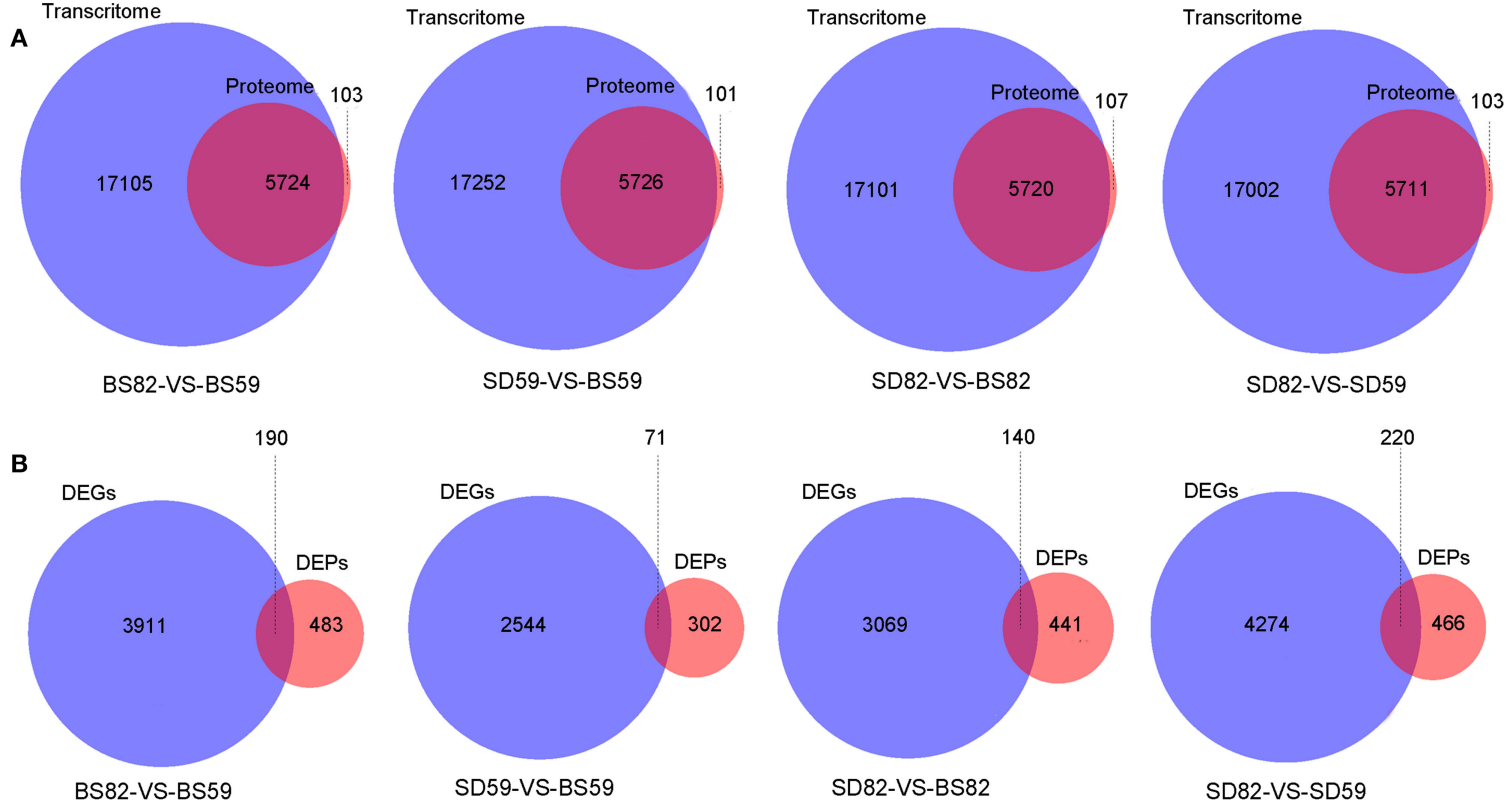
Venn diagrams of the correlation genes between proteome and transcriptome. **(A)** Venn diagram of identification gene number between the proteome and transcriptome. **(B)** The Venn diagram of DEG (differentially expressed gene) and DEP (differentially expressed protein) number from the proteome and transcriptome at four comparative groups.

The correlation analysis showed that 5724 (BS59-VS-BS82), 5726 (BS59-VS-SD59), 5720 (BS82-VS-SD82), and 5711 (SD59-VS-SD82) identification genes were correlated between the proteomic and transcriptomic, respectively (Figure 3A). Proteomic analysis showed that 673 (BS59-VS-BS82), 373 (BS59-VS-SD59), 581 (BS82-VS-SD82), and 686 (SD59-VS-SD82) DEPs were identified. A total of 621 genes (hereafter called cor-DEGs-DEPs), including 190, 71, 140, and 220 in the BS59-VS-BS82, BS59-VS-SD59, BS82-VS-SD82, and SD59-VS-SD82 groups, were regulated at both the mRNA and protein levels, respectively (Figure 3B). Among the 621 cor-DEGs-DEPs, 549 genes exhibited the same trend, including 163, 65, 124, and 198 in the BS59-VS-BS82, BS59-VS-SD59, BS82-VS-SD82, and SD59-VS-SD82 groups, respectively. A total of 72 cor-DEGs-DEPs exhibited the opposite trend (Supplementary Table 2), including 27, 6, 17, and 22 in the BS59-VS-BS82, BS59-VS-SD59, BS82-VS-SD82, and SD59-VS-SD82, respectively.

### GO and Pathway Enrichment Analysis of the cor-DEGs-DEPs

**Figure 4:**
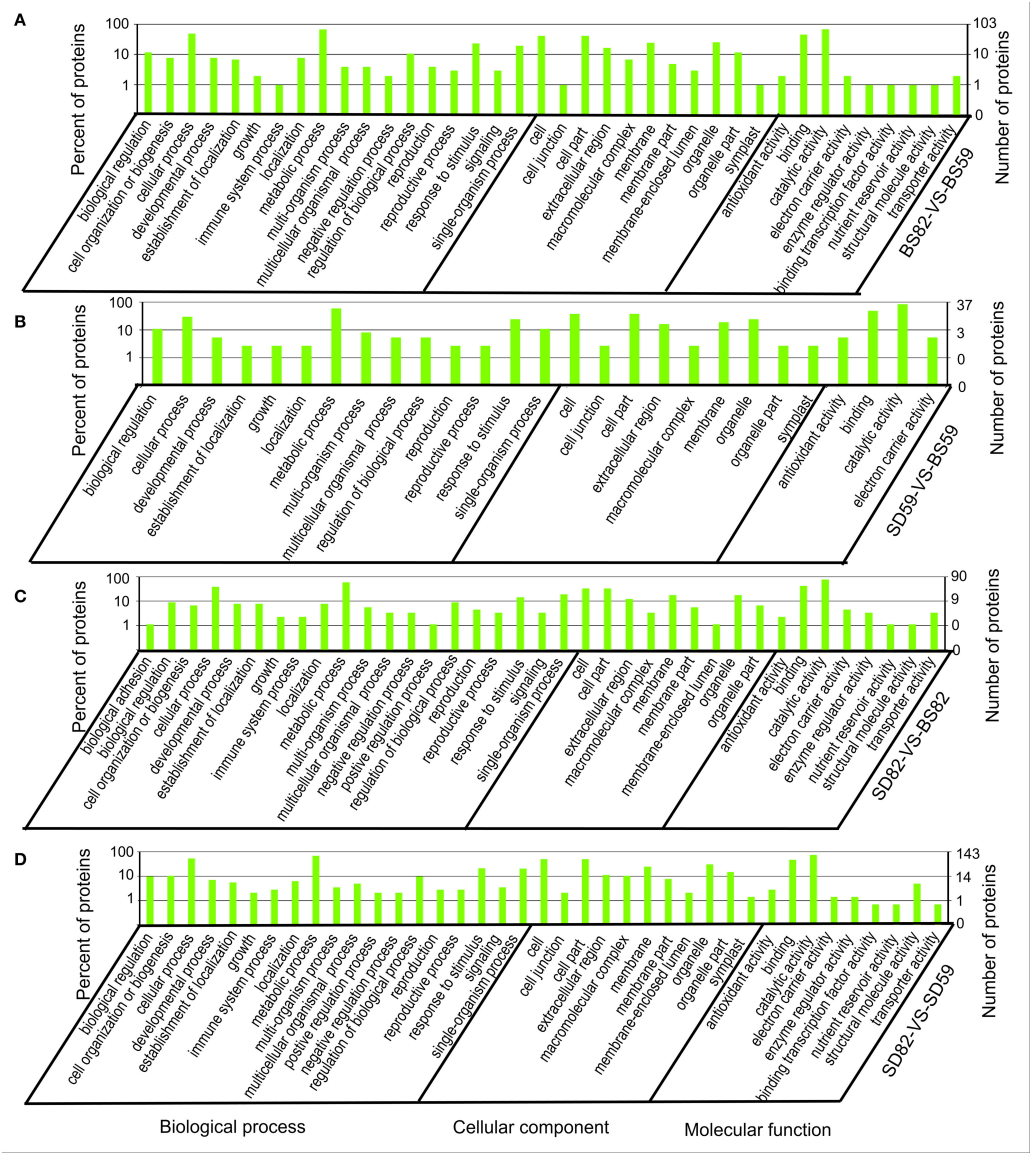
Gene Ontology enrichment analysis of the Cor-DEGs-DEPs (correlated differentially expressed genes and proteins) in the BS82-VS-BS59 **(A)**, SD59-VS-BS59 **(B)**, SD82-VS-BS82 **(C)**, and SD82-VS-SD59 **(D)** groups.

GO annotation results showed that 373 of the 621 cor-DEGs-DEPs were successfully annotated, including 103, 37, 90, and 143 cor-DEGs-DEPs found in the BS59-VS-BS82, BS59-VS-SD59, BS82-VS-SD82, and SD59-VS-SD82 groups (Figure 4). Among the four groups, 40 GO terms were identified and covered a wide range of cellular components, molecular functions, and biological processes. The two largest subcategories were found in the “biological processes” category, including “cellular process” and “metabolic process.” In the “cellular component” category, “cell” and “cell part” were the most abundant GO terms. In the “molecular function” category, two largest subcategories were “binding” and “catalytic activity.”

To better understand the function of cor-DEGs-DEPs, the Kyoto Encyclopedia of Genes and Genomes (KEGG) pathway analysis was performed using a P-value of less than 0.05 as the cutoff, and the result showed that 138 of the 190 (BS59-VS-BS82), 51 of the 71 (BS59-VS-SD59), 112 of the 140 (BS82-VS-SD82), and 170 of the 220 (SD59-VS-SD82) were mapped to 72, 38, 70, and 79 KEGG pathways, respectively (Supplementary Table 3). Two KEGG pathways were highly annotated at both the transcriptional and translational levels in the four groups, including “starch and sucrose metabolism” (ko00500) and “amino sugar and nucleotide sugar metabolism” (ko00520). Moreover, the KEGG pathways of “phenylpropanoid biosynthesis” (ko00940), “biosynthesis of amino acids” (ko01230), and “glutathione metabolism” (ko00480) were significantly enriched in the BS59-VS-SD59 and BS82-VS-SD82 groups, indicating that these processes can be highly differentiated between the SD and BS. In the BS59-VS-BS82 and SD59-VS-SD82 groups, “RNA transport” (ko03013), “protein processing in endoplasmic reticulum” (ko04141), “glyoxylate and dicarboxylate metabolism” (ko00630), “glycine, serine, and threonine metabolism” (ko00260), and “plant hormone signal transduction” (ko04075) were apparently enriched among the cor-DEGs-DEPs. Two cor-DEGs-DEPs were found in the ko04075 pathway. Bv_25960_dhcc.t1 encodes leucine-rich repeat receptor kinase (BRI1), which acts as a brassinolide receptor involving in brassinosteroid signal transduction. Bv2_044480_uecx.t1 encodes gibberellin receptor (GID1) that interacts with DELLA proteins and participates in ubiquitin-mediated proteolysis. These results suggest that the brassinosteroid and gibberellin signal transduction may play an important regulatory role in the taproot growth and development of sugar beet.

### Correlation Analysis of Significant GO Enrichment in the Proteome and Transcriptome

**Figure 5:**
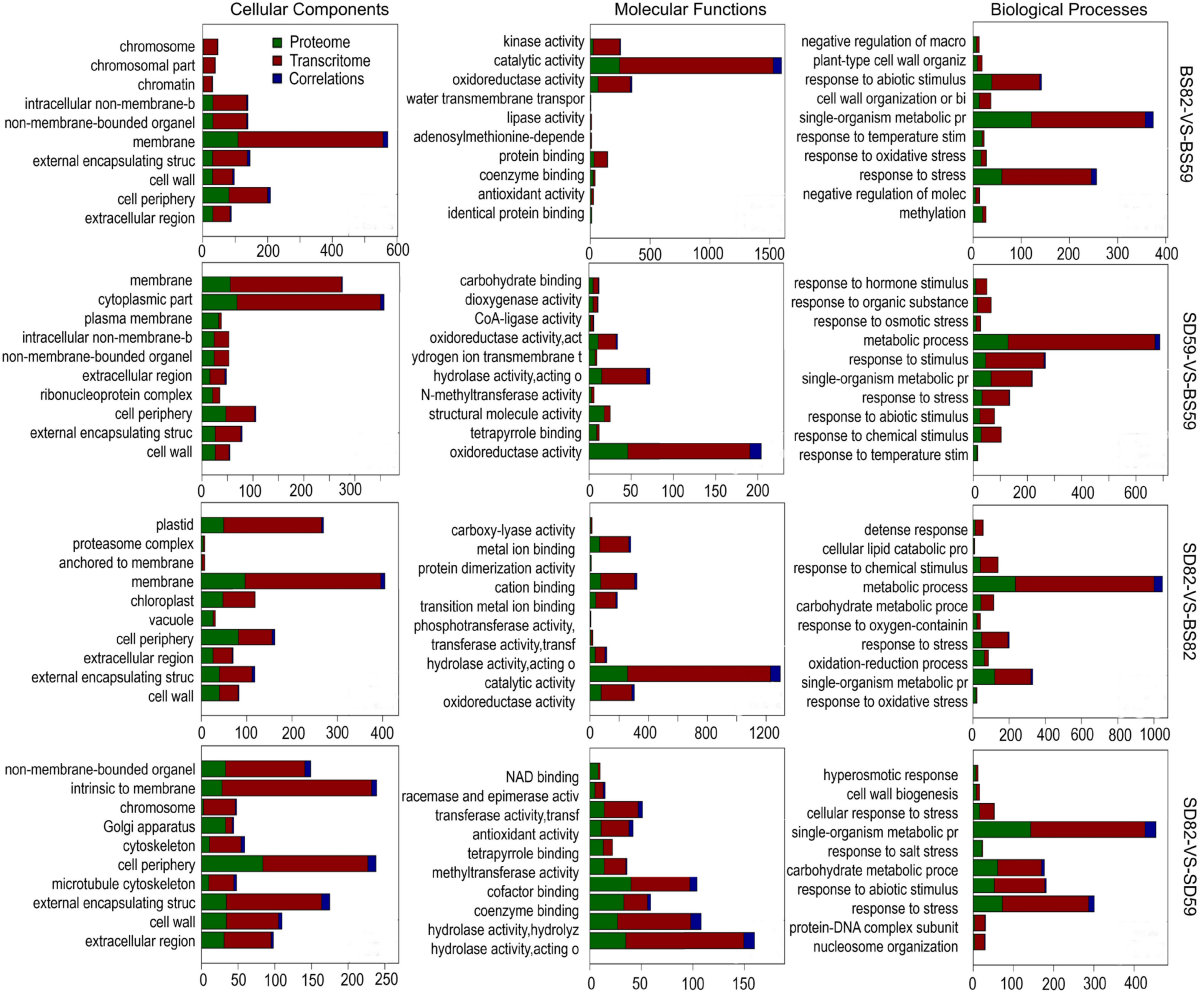
Gene Ontology enrichment analysis of the DEGs (differentially expressed genes) and DEPs (differentially expressed proteins) in the BS82-VS-BS59, SD59-VS-BS59, SD82-VS-BS82, and SD82-VS-SD59 groups at the transcriptome and proteome levels, respectively.

GO analysis of the DEGs and DEPs was performed in four comparative groups at the transcriptome and proteome levels, respectively. The results showed that several subcategories that are associated in the “cellular component” category were “external encapsulating structure,” “membrane,” “cell periphery,” “cytoplasmic part,” and “cell wall.” In the “molecular function” category, the correlation subcategories were “catalytic activity,” “oxidoreductase activity,” “hydrolase activity,” “kinase activity,” and “cation binding.” For the “biological process” category, “response to stress,” “single-organism metabolic process,” and “metabolic process” were correlated among the four groups (Figure 5).

**Figure 6:**
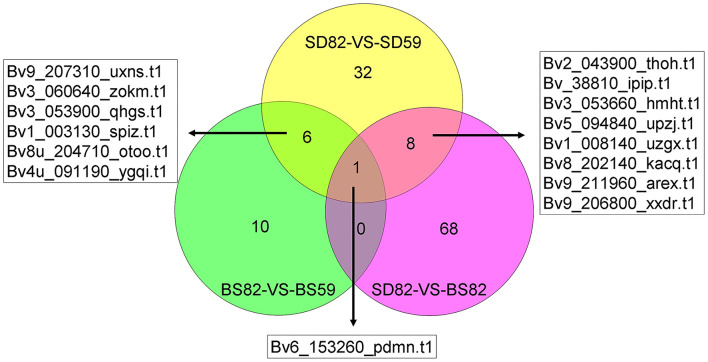
Numbers of shared and unique enrichment cor-DEGs-DEPs in the BS82-VS-BS59, SD82-VS-SD59, and SD82-VS-BS82 groups, and the gene ID of shared enrichment cor-DEGs-DEPs were indicated.

To better understand the regulatory mechanism of taproot growth and development, GO significant enrichment analysis for the DEGs and DEPs was performed using a P-value of less than 0.05 as the cutoff, and the correlation analysis of significantly enriched GO terms was conducted between transcriptome and proteome (Supplementary Figure 4). In the BS82-VS-BS59 groups, four of the significant GO enrichment terms in the proteome and transcriptome were mainly in the “cellular component” category. Ten terms were observed in the SD82-VS-SD59 group, including six in “cellular component,” two in “molecular function,” and two in “biological process.” Five terms were observed in the SD82-VS-BS82 group, including three in “cellular component” and two in “molecular function.” No terms were found in the SD59-VS-BS59 group (Supplementary Figure 5). We systematically integrated these cor-DEGs-DEPs, which were significantly enriched in both the transcriptome and the proteome. The results showed that 10, 32, and 68 cor-DEGs-DEPs were found in the BS82-VS-BS59, SD82-VS-SD59, and SD82-VS-BS82 groups, respectively (Figure 6). Among the three groups, only one cor-DEG-DEP was shared, encoding ribonuclease 1-like protein. Six cor-DEGs-DEPs were shared in the BS82-VS-BS59 and SD82-VS-SD59 groups, including the DEAD-box ATP-dependent RNA helicase (Bv9_207310_uxns.t1), TolB protein-like protein (Bv3_060640_zokm.t1), heat shock protein 83-like (Bv3_053900_qhgs.t1), aspartyl protease family protein 2 (Bv1_003130_spiz.t1), 20 kDa chaperonin, chloroplastic (Bv4u_091190_ygqi.t1), and xyloglucan endotransglucosylase/hydrolase protein 8 (Bv8u_204710_otoo.t1). Eight cor-DEGs-DEPs were shared in the SD82-VS-BS82 and SD82-VS-SD59 groups, encoding the aspartyl protease AED3 (Bv2_043900_thoh.t1), endoglucanase 2 (Bv_38810_ipip.t1), alpha-glucosidase (Bv3_053660_hmht.t1), probable polygalacturonase (Bv5_094840_upzj.t1), basic endochitinase (Bv1_008140_uzgx.t1), acidic mammalian chitinase (Bv8_202140_kacq.t1), ribosome-inactivating protein lychnin (Bv9_211960_arex.t1), and antiviral protein alpha isoform X2 (Bv9_206800_xxdr.t1) (Supplementary Table 4). The results suggested that these cor-DEGs-DEPs might be involved in the expansion of sugar beet taproot.

### Transcriptional Regulation May Be Involved in the Expansion of Sugar Beet Taproot

**Figure 7:**
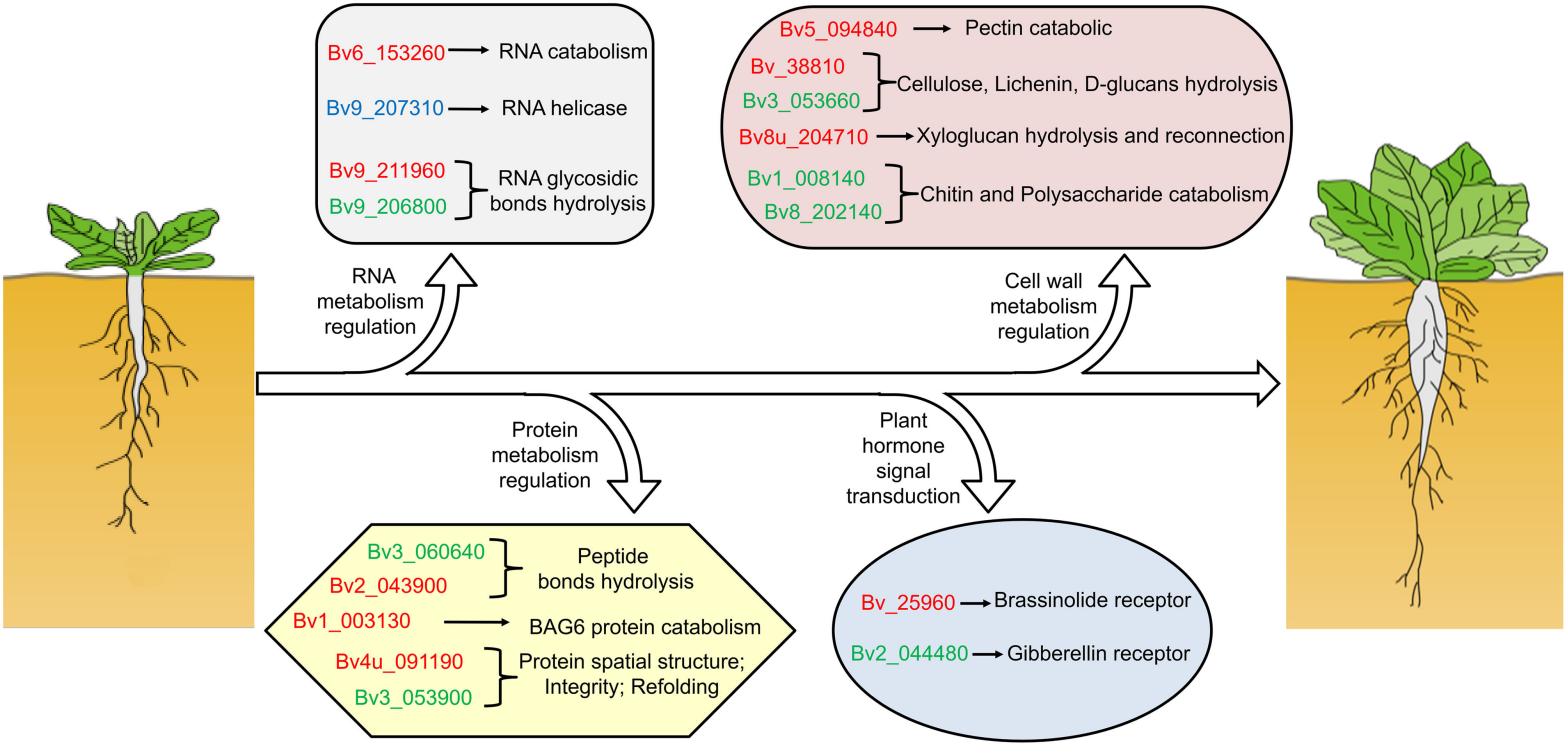
Schematic diagram of the regulation of sugar beet taproot growth and development. Taproot growth and expansion might be regulated at RNA metabolism, protein metabolism, and cell wall metabolism. RNA metabolism relative genes promote RNA catabolism, RNA helicase, and RNA glycosidic bond hydrolysis to restrict the formation of proteins in many processes. Protein metabolism relative genes involved in peptide bonds hydrolysis, BAG6 protein catabolism, and protein spatial structure refolding and maintaining protein integrity to assist in the correct posttranslational assembly of proteins. Cell wall metabolism relative genes involved in pectin, chitin, cellulose, lichenin, D-glucans, xyloglucan hydrolysis, and reconnection, promote the loosening and elongation of the cell wall, and improve cell elongation or expansion. Brassinolide and gibberellin receptor genes are enriched in pathway of plant hormone signal transduction. The red font indicates upregulated, and the green font represents downregulated in both transcriptome and proteome; and the blue font represents downregulated in transcriptome and upregulated in proteome.

Our previous study showed that the taproot of both SD and BS grew slowly before 59 DAE, and the growth rate of taproot was highest at 82 DAE. In addition, the growth rate of taproot in SD was significantly higher than that of BS at 82 DAE (Zhang et al., 2017). Some regulatory processes at the RNA level were significantly enriched in both the proteome and transcriptome data by comparison of SD82-VS-BS82, SD82-VS-SD59, and BS82-VS-BS59. For example, one shared enriched protein encoding a ribonuclease 1-like protein (Bv6_153260_pdmn.t1) was found in three comparative groups (Figure 6). This protein catalyzed the hydrolysis of ester linkages within ribonucleic acid by creating internal breaks to promote RNA catabolism, which also plays a role in remobilizing phosphate, particularly when cells senesce or when phosphate is limited. In the SD82-VS-SD59 and BS82-VS-BS59 groups, one shared enriched protein encoded a DEAD-box ATP-dependent RNA helicase (Bv9_207310_uxns.t1), which is necessary for mRNA export from the nucleus and can positively regulate the CBF/DREB transcription factors to enhance plant chilling and freezing tolerance (Gong et al., 2002, 2005). Two shared enriched proteins were found in the comparative groups SD82-VS-SD59 and SD82-VS-BS82 (Figure 6). These proteins were Bv9_211960_arex.t1 and Bv9_206800_xxdr.t1, encoding ribosome-inactivating protein lychnin and antiviral protein alpha isoform X2, which act as RNA glycosylases to catalyze the hydrolysis of N-glycosidic bonds in an RNA molecule, and play an important role in negative regulation of translation, restricting the formation of proteins in many processes (Figure 7). These results suggested that the transcriptional regulatory processes involving these proteins might play a crucial role in the growth and development of sugar beet taproot.

### Regulation of Protein Metabolism May Be Involved in the Expansion of Sugar Beet Taproot

Based on a combined analysis of the transcriptome and proteome, we found that some protein metabolism regulatory processes were significantly enriched by comparison of the SD82-VS-BS82, SD82-VS-SD59, and BS82-VS-BS59 groups. Four shared enriched proteins were found in the SD82-VS-SD59 and BS82-VS-BS59 groups: Bv3_060640_zokm.t1, Bv3_053900_qhgs.t1, Bv1_003130_spiz.t1, and Bv4u_091190_ygqi.t1, encoding TolB protein-like protein, heat shock protein 83-like, aspartyl protease family protein 2, and 20 kDa chaperonin, respectively (Figure 6 and Supplementary Table 4). TolB protein-like proteins can cleave peptide bonds to hydrolyze proteins into smaller polypeptides and/or amino acids. The heat shock protein 83-like maintains the structure and integrity of a protein, prevents it from degrading or aggregating, and promotes folding of single-chain polypeptides or multisubunit complexes into the correct tertiary structure. The aspartyl protease family protein 2 may act as an aspartic-type endopeptidase involved in protein catabolic processes of BAG6 and plant basal immunity (Li et al., 2016). The 20 kDa chaperonin is involved in chaperone cofactor-dependent protein refolding to assist in the correct posttranslational non-covalent assembly of proteins, which is also involved in the positive regulation of superoxide dismutase activity (Bonshtien et al., 2007; Kuo et al., 2013) and negative regulation of the abscisic acid-activated signaling pathway (Zhang et al., 2013) (Figure 7). In the SD82-VS-SD59 and SD82-VS-BS82 groups, Bv2_043900_thoh.t1 was a shared enriched protein, which encodes aspartyl protease AED3 and acts as aspartic-type endopeptidase, catalyzing the hydrolysis of internal alpha-peptide bonds in a polypeptide chain resulting in protein degradation (Figures 6, 7). This protein is also involved in the regulation of programmed cell death. These results suggested that these protein metabolism processes might play a crucial role in the expansion of sugar beet taproot.

### Regulation of Metabolism of the Cell Wall May Play an Important Role in the Expansion of Sugar Beet Taproot

The plant cell wall is mainly composed of cellulose, hemicellulose, tannin, and pectin and is involved in plant cell wall metabolism throughout the growth and development of plants. In this study, we found that many cell wall metabolism regulatory processes were significantly enriched in the SD82-VS-BS82, SD82-VS-SD59, and BS82-VS-BS59 groups. For example, Bv8u_204710_otoo.t1 was enriched in SD82-VS-SD59 and BS82-VS-BS59 (Figure 6). This protein encodes xyloglucan endotransglucosylase/hydrolase 8 protein (XTH8), can cleave and reconnect xyloglucan polymer through the hydrolysis of xyloglucan or internal transglycosylation, and then participate in the primary cell wall construction of plant growth tissue. In the SD82-VS-SD59 and SD82-VS-BS82 groups, five enriched genes were observed both in the proteome and transcriptome: Bv_38810_ipip.t1, Bv3_053660_hmht.t1, Bv5_094840_upzj.t1, Bv1_008140_uzgx.t1, and Bv8_202140_kacq.t1 (Figure 6). Bv_38810_ipip.t1 encodes an endoglucanase 2, which catalyzes the hydrolysis of beta-D-glucosidic linkages in cellulose, lichenin, and cereal beta-D-glucans, and may be involved in the sloughing (cell–cell separation) of root cap cells from the root tip of cell wall breakdown, which is important in plant development because it assists penetration of the growing root into the soil (Campillo et al., 2004). Bv3_053660_hmht.t1 encodes an alpha-glucosidase that catalyzes the hydrolysis of terminal alpha-D-glucosidic links in alpha-D-glucans, which are essential for stable accumulation of EFR (Burn et al., 2002; Lu et al., 2009). Bv5_094840_upzj.t1 is a probable polygalacturonase involved in cell separation in the final stages of pod shatter, resulting in the breakdown of the cell wall, and the pectin catabolic process, resulting in the breakdown of pectin, a polymer containing a backbone of alpha-1,4-linked D-galacturonic acid residues (González-Carranza et al., 2007; Ogawa et al., 2009). Bv1_008140_uzgx.t1 and Bv8_202140_kacq.t1 encode an endochitinase, which catalyzes the hydrolysis of N-acetyl-beta-D-glucosaminide (1->4)-beta-linkages in chitin and chitodextrins and are involved in cell wall macromolecule, chitin, and polysaccharide catabolism (Figure 7). These results suggested that cell wall metabolism plays important roles in sugar beet root enlargement.

### *Beta vulgaris XTH8* (*BvXTH8*) Can Enhance the Growth and Development of Arabidopsis Primary Roots by Improving Cell Growth in the Root Tip Elongation Zone

**Figure 8:**
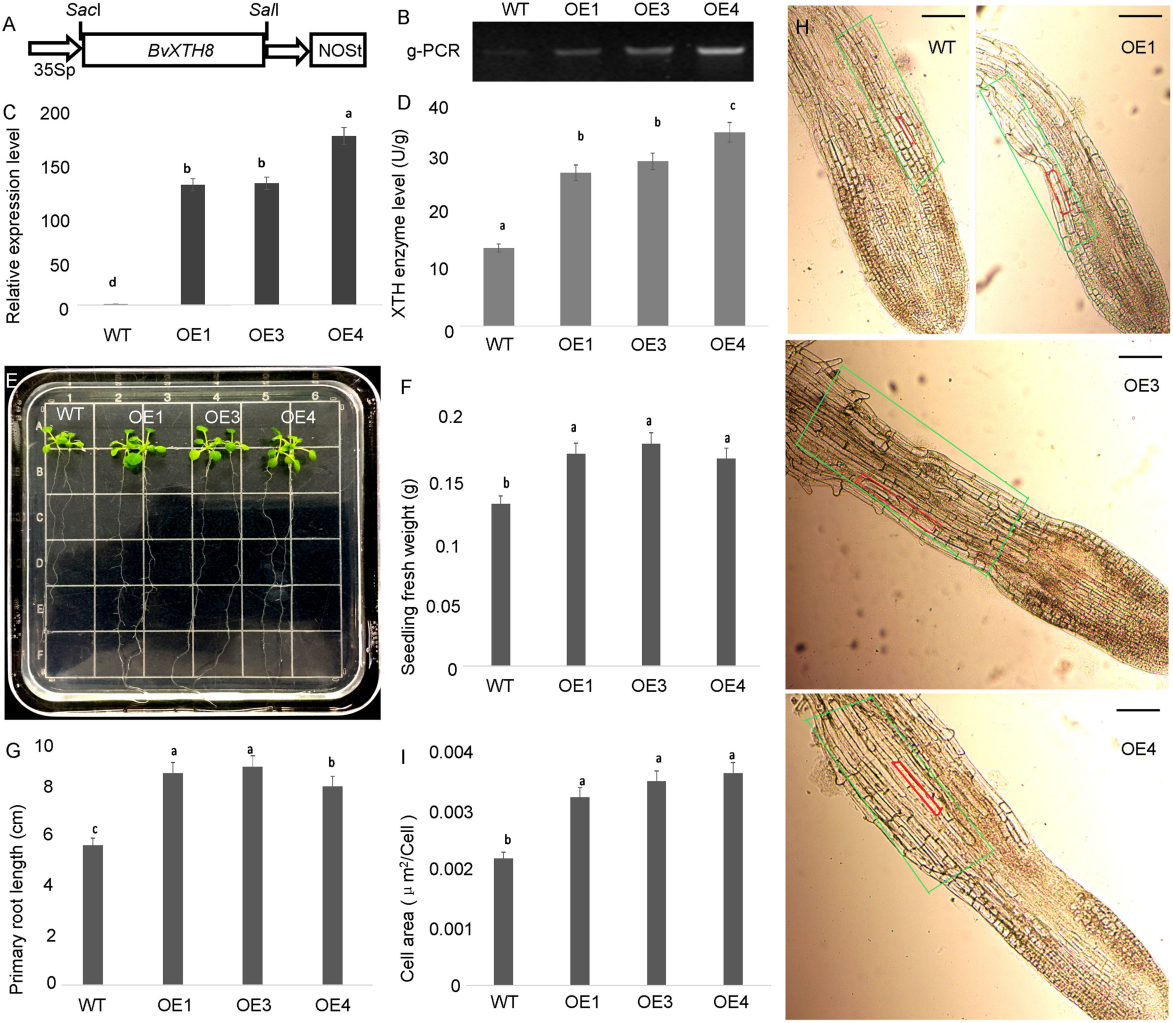
Functional identification of xyloglucan endotransglucosylase/hydrolase 8 (*BvXTH8*) from *Beta vulgaris*. **(A)** T-DNA region of the vector pCAMBIA1300-BvXTH8 used to produce transgenic plants. **(B)** Genomic PCR analysis to confirm insertion of *BvXTH8* into the genome of the transgenic lines. **(C)** The relative expression levels of *BvXTH8* were quantified in wild-type (WT) and transgenic plants by real-time RT-PCR. **(D)** BvXTH enzyme levels of WT and transgenic plants. **(E)** WT and transgenic plants grown on MS medium for 2 weeks, and measurements of the seedling fresh weight **(F)** and primary root length **(G)**. The microscopic observation of roots in WT and transgenic plants **(H)**, and the elongation zone areas of WT and transgenic plants were counted **(I)**. The superscript letters indicates the values which are not significantly different (*P* < 0.05, Duncan's MRT). The scale bar represents 100 μm. Data are expressed as means ± standard errors (*n* = 5).

To analyze the functions of *BvXTH8* (Bv8u_204710_otoo.t1), we constructed the pCAMBIA1300-BvXTH8 plasmid and introduced it into Arabidopsis plants through Agrobacterium-mediated transformation (Figure 8A). Homozygous transgenic lines were obtained using the self-pollination. Three homozygous lines overexpressing *BvXTH8* were validated by genomic PCR (Figure 8B). Real-time RT-PCR results indicate that the *BvXTH8* transcriptional levels in transgenic plants were significantly higher than in the wild type (WT) (Figure 8C). Measurement of XTH enzyme levels from WT and transgenic plants revealed that transgenic plants expressing *BvXTH8* had higher XTH activity than the WT (Figure 8D). These results suggested that the *BvXTH8* is stably expressed at transcript and protein levels in Arabidopsis.

Transgenic Arabidopsis seedlings overexpressing *BvXTH8* exhibited higher primary root lengths and fresh weights compared with WT plants at 2 weeks after sowing (Figures 8E–G). The microscopic observation of cells in the elongation zone showed that cells in the root tip elongation zone of transgenic plants were significantly longer than that of WT (Figure 8H), and cells in the elongation zone of transgenic plants were significantly increased compared to the WT (Figure 8I). These results indicate that *BvXTH8* can enhance the growth and development of Arabidopsis primary roots by improving cell growth in the root tip elongation zone.

## Discussion

Recent studies have shown that the correlations between the transcriptome and proteome can effectively identify target genes. For example, 792 and 1496 DEGs (transcriptome) and 404 and 870 DEPs (proteome) were identified to be more responsive to *C. carunculoides* infection in stage 1 and stage 2, respectively, than the control samples; however, only 47 and 120 genes were found to be responsive to *C. carunculoides* infection in stage 1 and stage 2 through correlated analysis of the transcriptome and proteome (Dai et al., 2019). Similarly, in this study, 3183, 966, 1164, and 3250 DEGs (transcriptome) and 673, 373, 581, and 686 DEPs (proteome) were found in the BS59-VS-BS82, BS59-VS-SD59, BS82-VS-SD82, and SD59-VS-SD82 groups, respectively (Figure 1). However, correlation of the transcriptome and proteome showed that only 190, 71, 140, and 220 genes were found in the BS59-VS-BS82, BS59-VS-SD59, BS82-VS-SD82, and SD59-VS-SD82 groups, respectively (Figure 3B), indicating that transcriptome and proteome association analysis greatly narrows the scope of target genes and efficiently identifies candidate genes related to plant root development.

*Beta vulgaris* is an important fleshy taproot sugar crop. Therefore, it is important to understand the mechanism of sugar beet taproot formation. Studies have shown that three interrelated processes may be involved in the sugar beet taproot growth: cell division in the secondary meristem rings (Hayward, 1938), the derivative accumulation of the cambiums cell (Elliott and Weston, 1993), and carbohydrate accumulation of parenchymal cell (Bellin et al., 2007). Recently, some studies have explained the sucrose accumulation in the developing taproot of sugar beet by cDNA-amplified fragment length polymorphism (Trebbi and McGrath, 2009), and the taproot-expressed candidate genes have been screened by EST sequencing (Bellin et al., 2002). Our previous transcriptome showed that many GO terms were involved in taproot growth and development, including the cell wall, cytoskeleton, enzyme-linked receptor protein signaling pathway, and multiple hormone metabolism pathway, such as auxin, gibberellin, cytokinin, and brassinosteroid, also play important roles in the taproot growth and development of sugar beet (Zhang et al., 2017). However, so far, the research on the molecular regulation mechanism of taproot growth and development is still not sufficient. In this study, the transcriptomic and proteomic correlations of the taproot initial growth stage and the maximum growth rate stage in two cultivars were performed to explore the molecular regulatory mechanisms of taproot growth in sugar beet.

Recently, many studies have shown using transcriptomic data analysis techniques that key GO terms and KEGG pathways may be involved in plant taproot development. For example, some GO terms, such as “cell wall,” “regulation of biological process,” and “cytoskeleton,” are involved in taproot development of radish and carrot (Wang et al., 2015; Yu et al., 2016), and the secondary metabolite biosynthesis pathway and starch and sucrose metabolic pathway were associated with secondary taproot thickening in radish (Mitsui et al., 2015; Sun et al., 2015). Previously, we found that the GO terms, such as “cell wall,” “enzyme-linked receptor protein signaling pathway,” and “cytoskeleton,” were enriched at 82 DAE, when the sugar beet taproot enters the most rapid growth stage. The plant hormone signaling transduction pathway and starch and sucrose metabolism pathway were involved in sugar beet taproot growth and sucrose accumulation during growth stages (Zhang et al., 2017). In this study, we performed GO and KEGG pathway enrichment analyses based on the correlation of DEGs and DEPs and found that several GO terms, such as “external encapsulating structure,” “cell wall,” “catalytic activity,” “single-organism metabolic process,” and “response to stress,” were significantly correlated in the four groups (Figure 5). In addition, the RNA transport, protein processing in endoplasmic reticulum, and plant hormone signal transduction showed a significant correlations in the KEGG enrichment of DEGs and DEPs. In particular, *BRI1* and *GID1* can encode receptor proteins involving in brassinosteroid and gibberellin signal transduction, thereby regulating taproot growth and development of sugar beet (Figure 7). Among these GO terms, many metabolic regulatory processes at the level of transcription and translation were significantly enriched in both proteome and transcriptome data of the comparison groups. At the transcriptional level, the ribonuclease 1-like protein involved in RNA catabolic process and the DEAD-box ATP-dependent RNA helicase is indispensable for mRNA export from the nucleus (Gong et al., 2002, 2005). Furthermore, the ribosome-inactivating protein lychnin and antiviral protein alpha isoform X2 are involved in negative regulation of translation restricted protein production (Figure 7). At the translational level, TolB protein-like protein, aspartyl protease AED3, and aspartyl protease family protein 2 are involved in the hydrolysis of proteins into smaller polypeptides, and regulation of programmed cell death and plant basal immunity (Li et al., 2016), and heat shock protein 83-like and 20 kDa chaperonin are involved in maintaining the structure and integrity of a protein and facilitating folding of single-chain polypeptides into the correct tertiary structure (Figure 7) (Bonshtien et al., 2007; Kuo et al., 2013). The data suggested that the cor-DEGs-DEPs enriched in these GO terms were responsible for the growth and development of sugar beet taproot *via* the regulation of these metabolic processes at the transcriptional and translational levels.

Plant root growth and development is a complex biological process, which contains the first and second vascular cambia initiation, and secondary xylem and phloem development (Yu et al., 2016). During plant growth, the first and second vascular cambia initiations are mainly stimulated by an external signal factor, and secondary xylem and phloem development, such as cell differentiation, division, and expansion, is mainly regulated by a series of metabolic processes, such as cell wall metabolism, starch and sucrose metabolism, and storage and energy metabolism (Petricka et al., 2012). Recently, the cell wall metabolism-related regulatory genes have been extensively studied in other plant species (Liu et al., 2013; Lakhotia et al., 2014), and the roles of these genes in root development were also determined (Khan et al., 2011; Bustos-Sanmamed et al., 2013). In radish, the *ARFs, IAA11*, and *bHLH74* are involved in root development and regulate vascular cell differentiation (Bao et al., 2014; Zhang et al., 2014c). The LRR protein kinase-like protein gene, *LACs*, and *EXPA9* are involved in cell wall formation and loosening (Dolan and Davies, 2004; Cai et al., 2006), whereas *CESA6* and *BAM4* are participated in cell wall synthesis and degradation (Cosgrove, 2005; Van Sandt et al., 2007). In sugar beet, the taproot rapid growth may be regulated by cell wall elongation, cell mitosis, and cell growth metabolism, such as the enzyme beta-glucosidase and pectinesterase may be necessary for cell wall elongation metabolism (Moustacas et al., 1991); the kinesin C2/C3 and LC8 dynein light chain may play important roles in regulating the cell mitosis metabolism (Xiao et al., 2010; Mary et al., 2015); and the brassinosteroid insensitive protein 1, gibberellin-regulated protein 1, FER, and HAIKU2 receptor-like kinase may be involved in the cell growth metabolism (Luo et al., 2005; Guo et al., 2009; She et al., 2011; Jiang et al., 2013). In this study, many cor-DEGs-DEPs related to cell wall metabolism were observed in both the proteome and transcriptome: Endoglucanase 2 catalyzes the hydrolysis of cellulose and is involved in the sloughing of root cap cells from the root tip (Campillo et al., 2004); polygalacturonase is involved in the cell separation of the final stages of pod shatter, resulting in breakdown of the cell wall, and pectin catabolism, resulting in the breakdown of pectin (González-Carranza et al., 2007); endochitinase catalyzes the hydrolysis of chitin and chitodextrins and is involved in the catabolism of cell wall macromolecules, chitin, and polysaccharides (Ogawa et al., 2009); XTH8 cleaves and religates xyloglucan polymers and is involved in cell wall construction of growing tissues and the accumulation of hemicelluloses (Figure 7) (Han et al., 2016). We also further confirmed that *BvXTH8* could promote the elongation and growth of cells in the elongation zone of plant roots through heterologous expression in *Arabidopsis* (Figure 8), which is consistent with previous reports by Vissenberg et al. (2005) and Lee et al. (2010, 2018). These results suggested that the processes of cell wall metabolism play an important role in taproot development and thickening of sugar beet, which may regulate the catabolism and biosynthesis of cell wall macromolecular substances, promote the loosening and elongation of the cell wall, improve cell elongation or expansion, and improve the development and thickening of the beet taproot system.

## Conclusion

In this study, the transcriptomics and proteomics of two cultivars (SD and BS) on the taproot initial growth stage and the maximum growth rate stage were correlated to explore the molecular regulatory mechanisms of taproot growth in sugar beet. The correlation analysis of the proteome and transcriptome showed that taproot growth and expansion might be regulated at transcriptional and posttranscriptional, and the hormone signal transduction, which also may be attributed to cell wall metabolism to promote cell wall loosening and elongation and improve cell elongation or expansion. This work provides insight into the molecular mechanism of taproot growth and development and facilitates the genetic engineering of new sugar beet cultivars with a high yield and quality.

## Data Availability Statement

The original contributions presented in the study are publicly available. This data can be found here: RNA-seq data was deposited in NCBI SRA BioProject https://www.ncbi.nlm.nih.gov/sra/?term=SRP090408; proteomic data was deposited in ProteomeXchange under accession no PXD031889.

## Author Contributions

SZ conceived and supervised this study. SZ, YS, GL, and NL designed experiments. YZ, XW, and HM performed the experiments. NL analyzed and interpreted the data and wrote the manuscript. SZ, GL, and YS participated in the discussion and provided valuable advice and practical contributions. All the authors approved the final version of the manuscript prior to submission.

## Funding

This study was supported by the National Natural Science Foundation of China (32060506 and 31760416) and the Advanced Talents Research Foundation of Inner Mongolia Agricultural University (NDYB2018-11).

## Conflict of Interest

The authors declare that the research was conducted in the absence of any commercial or financial relationships that could be construed as a potential conflict of interest.

## Publisher's Note

All claims expressed in this article are solely those of the authors and do not necessarily represent those of their affiliated organizations, or those of the publisher, the editors and the reviewers. Any product that may be evaluated in this article, or claim that may be made by its manufacturer, is not guaranteed or endorsed by the publisher.
