# Supplementary material for: Integration of Transcriptomic and Proteomic Profiles Reveals Multiple Levels of Genetic Regulation of Taproot Growth in Sugar Beet (Beta vulgaris L.)
Source: Front Plant Sci. 2022 Jul 13;13:882753. doi: 10.3389/fpls.2022.882753 (PMC9326478; doi:10.3389/fpls.2022.882753)
Supplement: Supplementary file 1 [file Data_Sheet_1.docx]

Supplementary Figures


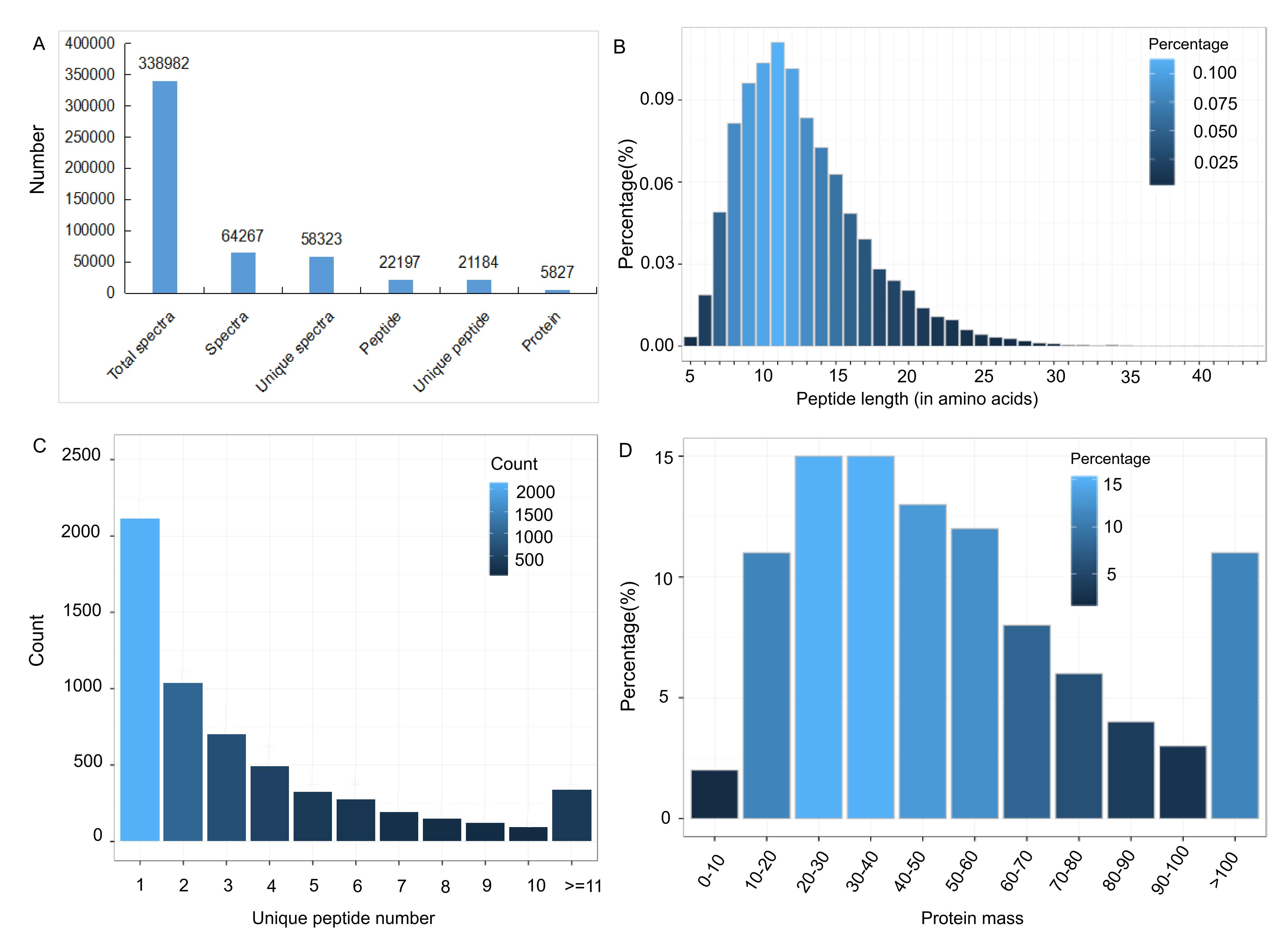


**Supplementary Figure 1**. Overview of the proteomic results. (**A**) Protein identification overview. (**B**) Peptide length distribution. (**C**) Peptide number distribution. (**D**) The protein mass distribution.


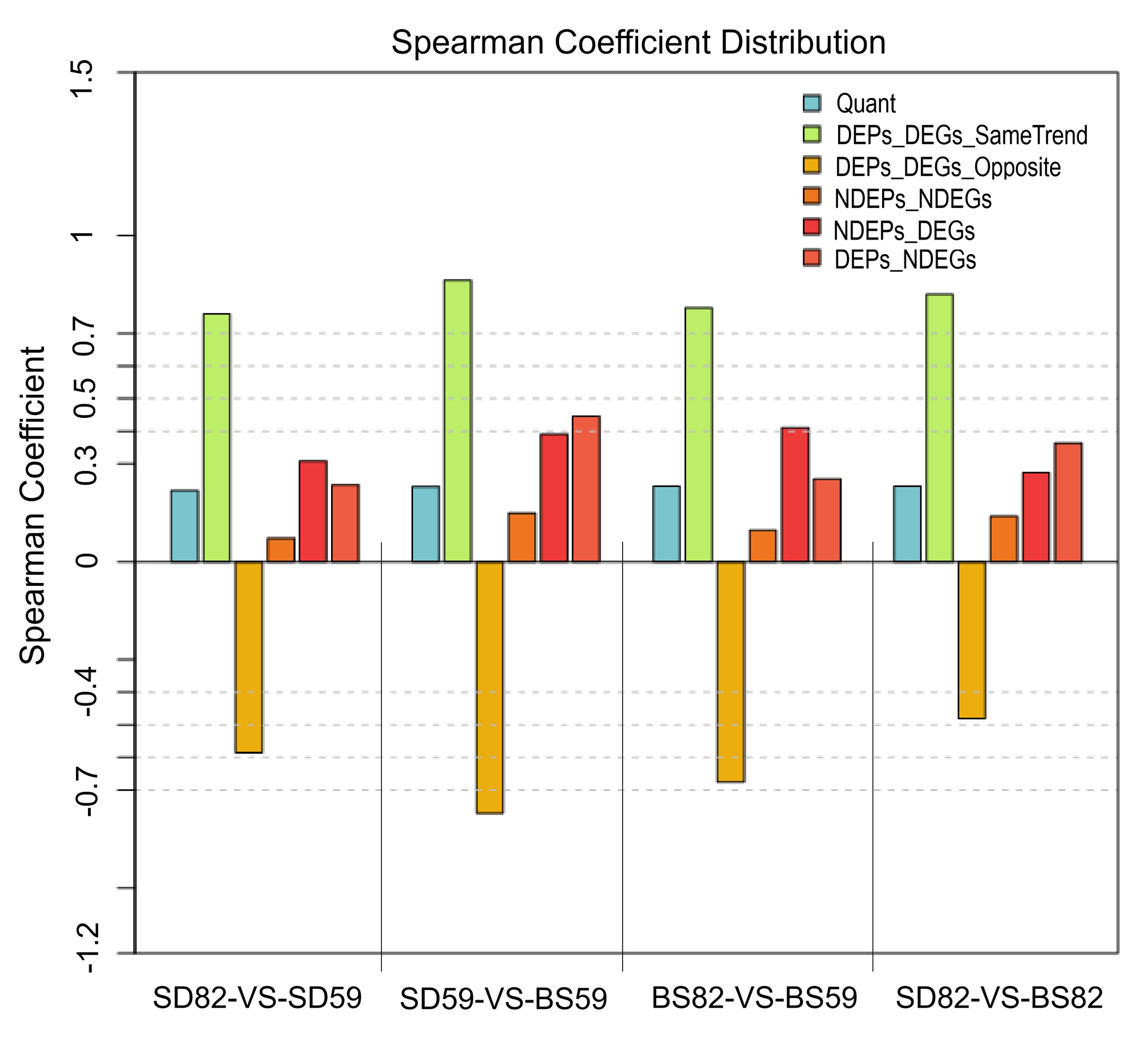


**Supplementary Figure 2**. Statistics of the correlation coefficients of quantitative correlation results of four comparative groups divided into five categories.


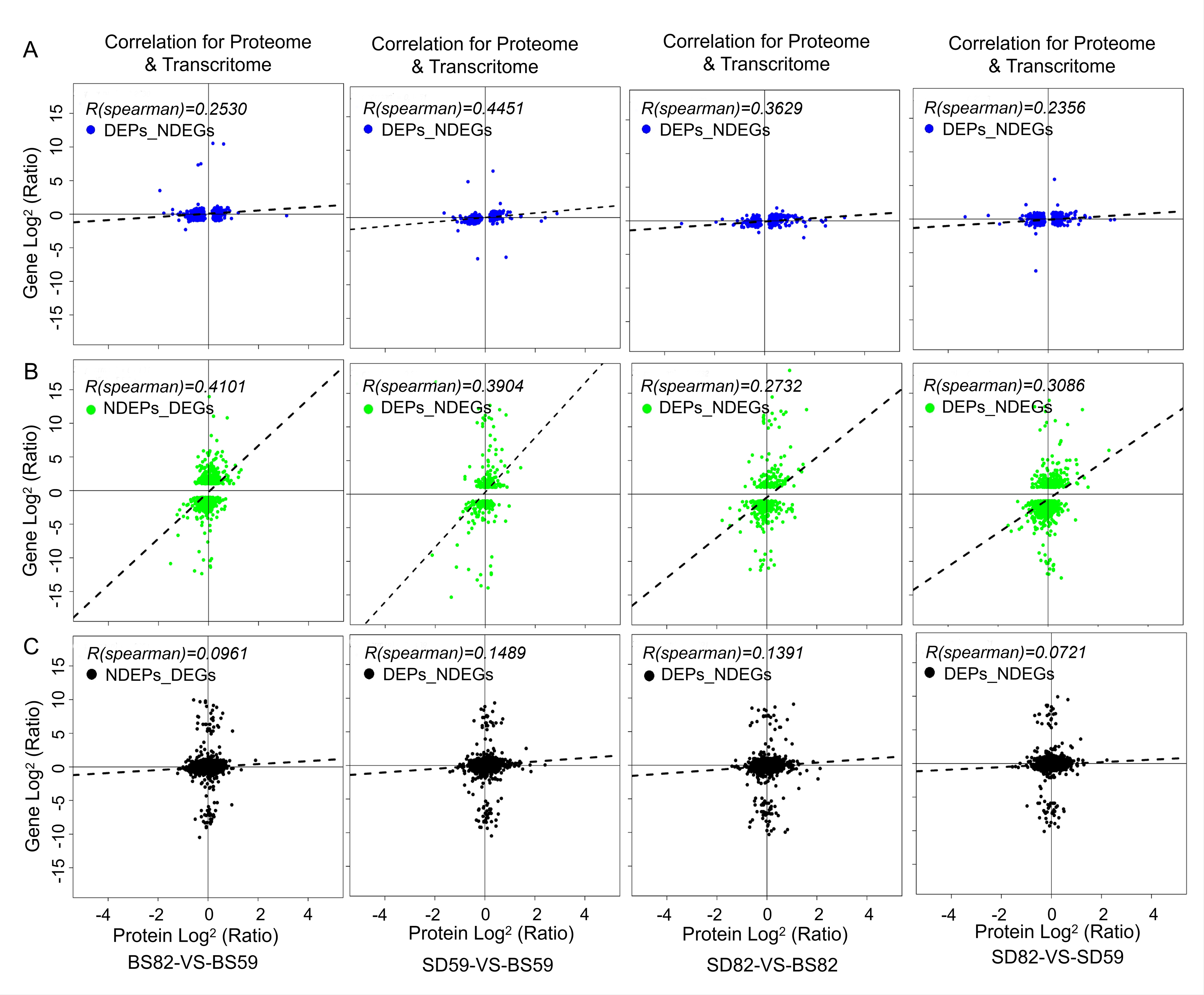


**Supplementary Figure 3**. Scatterplots and correlation coefficients between DEPs and NDEGs (**A**), NDEPs and DEGs (**B**), and NDEPs and NDEGs (**C**).


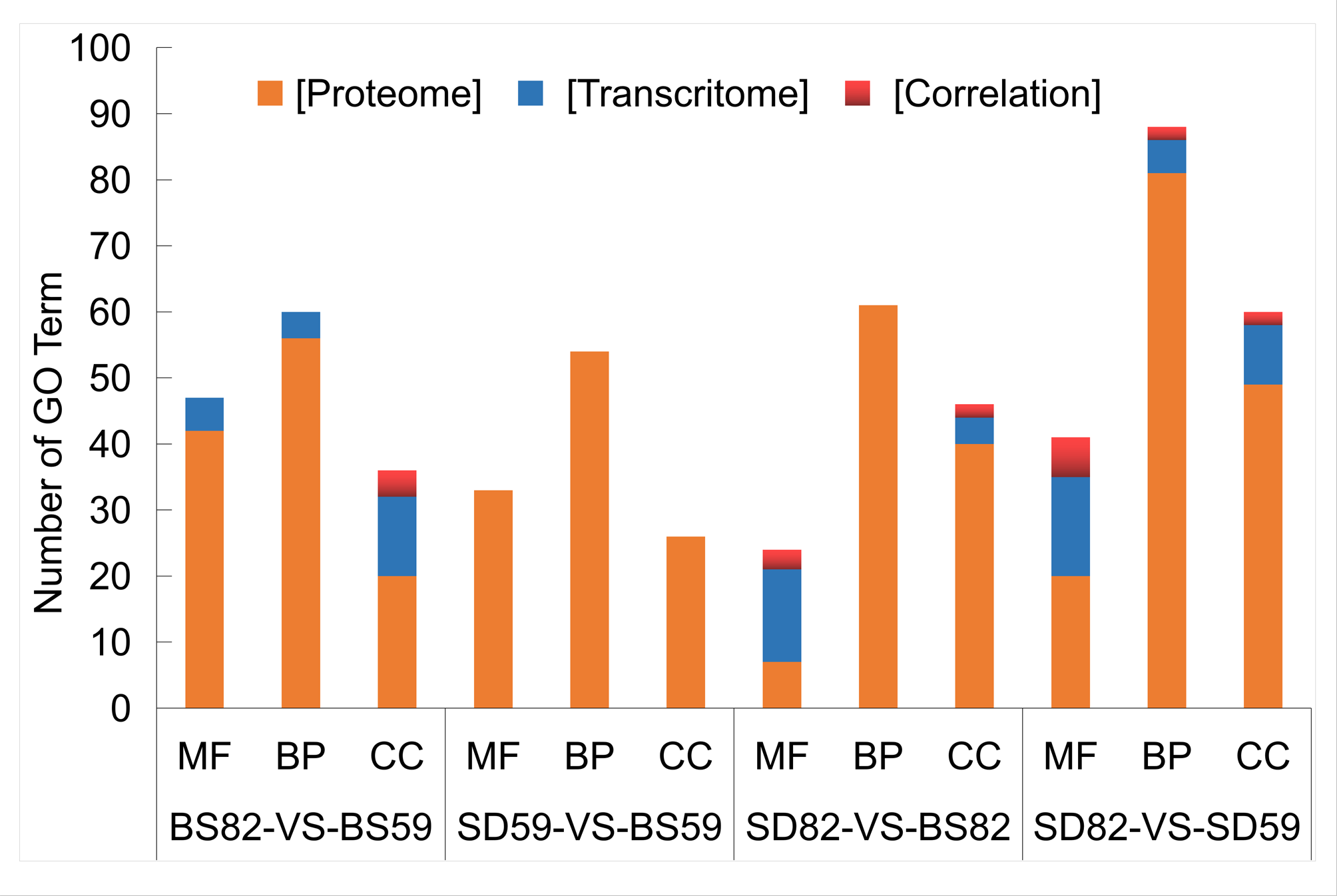


**Supplementary Figure 4**. Number of significant enrichment GO terms in the BS82-VS-BS59, SD59-VS-BS59, SD82-VS-BS82, and SD82-VS-SD59 groups at transcriptome and proteome


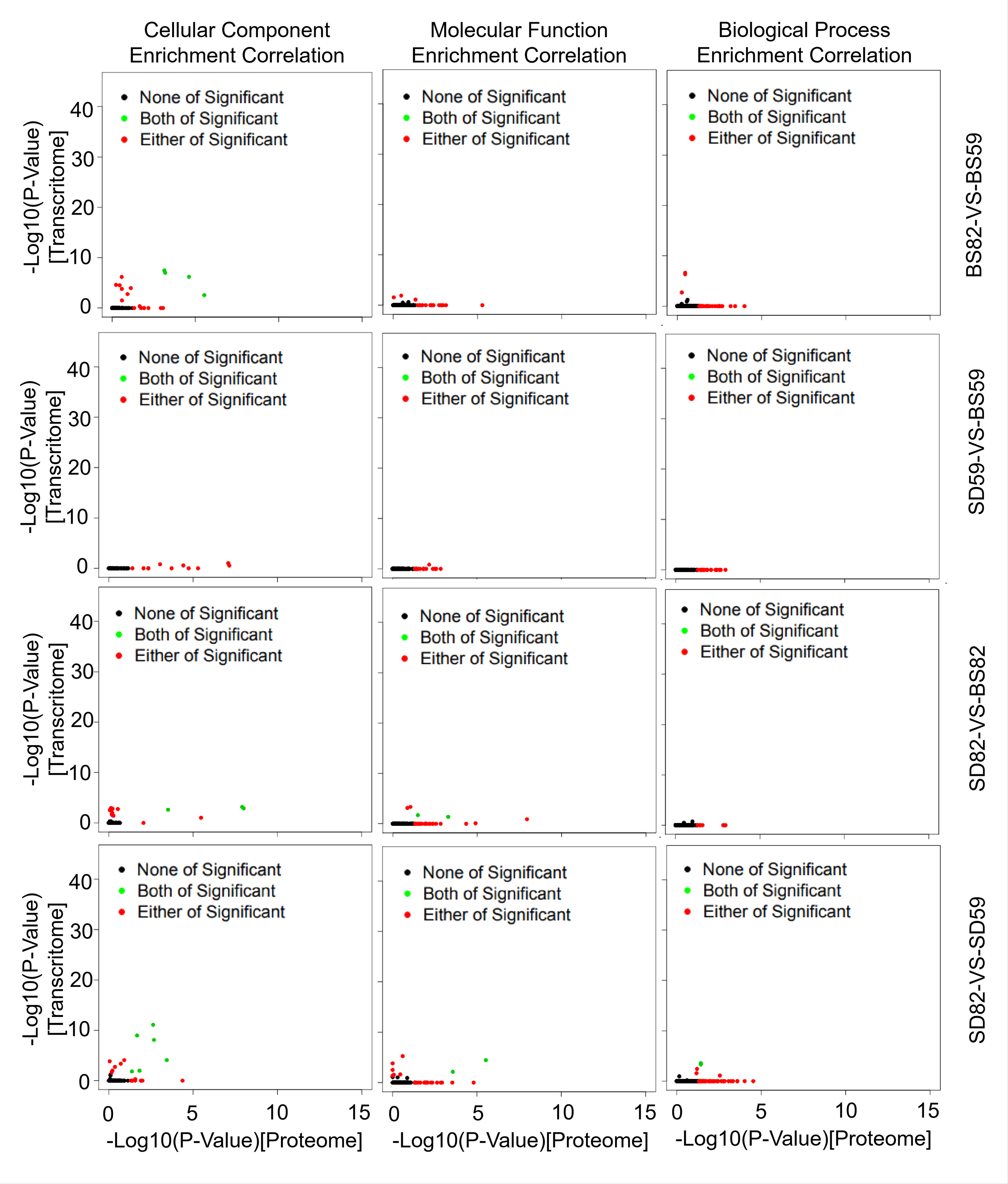


**Supplementary Figure 5**. Correlation analysis of significant enrichment GO terms in the BS82-VS-BS59, SD59-VS-BS59, SD82-VS-BS82, and SD82-VS-SD59 groups between at transcriptome and proteome.
