# Supplementary material for: Integration of Transcriptomic and Proteomic Profiles Reveals Multiple Levels of Genetic Regulation of Taproot Growth in Sugar Beet (Beta vulgaris L.)
Source: Front Plant Sci. 2022 Jul 13;13:882753. doi: 10.3389/fpls.2022.882753 (PMC9326478; doi:10.3389/fpls.2022.882753)
Supplement: Supplementary file 2 [file Data_Sheet_2.docx]

Supplementary Tables

**Supplementary Table 1**. Summary of transcriptome mapping results.

| Sample Name | Total Reads | Total Mapped  Reads | Unique Match | Multi- position  Match | Total Unmapped  Reads |
| --- | --- | --- | --- | --- | --- |
| BS59 | 50,575,736  (100.00%) | 41,856,308  (82.76%) | 35,980,490  (71.14%) | 5,875,818  (11.62%) | 8,719,426  (17.24%) |
| BS82 | 50,438,146  (100.00%) | 41,474,712  (82.23%) | 35,713,530  (70.81%) | 5,761,182  (11.42%) | 8,963,432  (17.77%) |
| SD59 | 50,920,054  (100.00%) | 42,166,760  (82.81%) | 36,431,802  (71.55%) | 5,734,958  (11.26%) | 8,753,292  (17.19%) |
| SD82 | 50,892,430  (100.00%) | 42,327,024  (83.17%) | 36,399,996  (71.52%) | 5,927,028  (11.65%) | 8,565,404  (16.83%) |

**Supplementary Table 2**. Summary of cor-DEGs-DEPs genes results in four comparative groups.

|  | NO.of the cor‐DEGs‐DEPs genes | NO. of the same trend DEGs‐DEPs | NO. of the opposite trend DEGs‐DEPs |
| --- | --- | --- | --- |
| BS59-VS-BS82 | 190 | 163 | 27 |
| BS59-VS-SD59 | 71 | 65 | 6 |
| BS82-VS-SD82 | 140 | 123 | 17 |
| SD59-VS-SD82 | 220 | 198 | 22 |
| Total | 621 | 549 | 72 |

**Supplementary Table 3**. Pathway enrichment analyses of cor-DEGs-DEPs in four comparative groups.

| **BS82-VS-BS59_Cor_DEGs_DEPs** | | | |
| --- | --- | --- | --- |
| # | Pathway | Diff Proteins with pathway annotation (138) | Pathway ID |
| 1 | [Metabolic pathways](file:///E:\\%E7%8E%8B%E9%9B%AA%E5%B3%B0\\iTRAQ\\%E5%8D%8E%E5%A4%A7%E5%9F%BA%E5%9B%A0\\%E5%8D%8E%E5%A4%A7%E6%95%B0%E6%8D%AE\\%E8%9B%8B%E7%99%BD%E8%B4%A8%E7%BB%84%E4%B8%8E%E8%BD%AC%E5%BD%95%E7%BB%84%E5%85%B3%E8%81%94%E5%88%86%E6%9E%90\\F15FTSNCKF3616\\F15FTSNCKF3616\\Correlation\\SubCorrelation2Function\\Pathway\\BS_82-VS-BS_59_Cor_DEPs_Pathway\\BS_82-VS-BS_59_Cor_DEPs.htm#gene1#gene1) | 45 (32.61%) | ko01100 |
| 2 | [Biosynthesis of secondary metabolites](file:///E:\\%E7%8E%8B%E9%9B%AA%E5%B3%B0\\iTRAQ\\%E5%8D%8E%E5%A4%A7%E5%9F%BA%E5%9B%A0\\%E5%8D%8E%E5%A4%A7%E6%95%B0%E6%8D%AE\\%E8%9B%8B%E7%99%BD%E8%B4%A8%E7%BB%84%E4%B8%8E%E8%BD%AC%E5%BD%95%E7%BB%84%E5%85%B3%E8%81%94%E5%88%86%E6%9E%90\\F15FTSNCKF3616\\F15FTSNCKF3616\\Correlation\\SubCorrelation2Function\\Pathway\\BS_82-VS-BS_59_Cor_DEPs_Pathway\\BS_82-VS-BS_59_Cor_DEPs.htm#gene2#gene2) | 27 (19.57%) | ko01110 |
| 3 | [Protein processing in endoplasmic reticulum](file:///E:\\%E7%8E%8B%E9%9B%AA%E5%B3%B0\\iTRAQ\\%E5%8D%8E%E5%A4%A7%E5%9F%BA%E5%9B%A0\\%E5%8D%8E%E5%A4%A7%E6%95%B0%E6%8D%AE\\%E8%9B%8B%E7%99%BD%E8%B4%A8%E7%BB%84%E4%B8%8E%E8%BD%AC%E5%BD%95%E7%BB%84%E5%85%B3%E8%81%94%E5%88%86%E6%9E%90\\F15FTSNCKF3616\\F15FTSNCKF3616\\Correlation\\SubCorrelation2Function\\Pathway\\BS_82-VS-BS_59_Cor_DEPs_Pathway\\BS_82-VS-BS_59_Cor_DEPs.htm#gene3#gene3) | 9 (6.52%) | ko04141 |
| 4 | [Carbon metabolism](file:///E:\\%E7%8E%8B%E9%9B%AA%E5%B3%B0\\iTRAQ\\%E5%8D%8E%E5%A4%A7%E5%9F%BA%E5%9B%A0\\%E5%8D%8E%E5%A4%A7%E6%95%B0%E6%8D%AE\\%E8%9B%8B%E7%99%BD%E8%B4%A8%E7%BB%84%E4%B8%8E%E8%BD%AC%E5%BD%95%E7%BB%84%E5%85%B3%E8%81%94%E5%88%86%E6%9E%90\\F15FTSNCKF3616\\F15FTSNCKF3616\\Correlation\\SubCorrelation2Function\\Pathway\\BS_82-VS-BS_59_Cor_DEPs_Pathway\\BS_82-VS-BS_59_Cor_DEPs.htm#gene4#gene4) | 9 (6.52%) | ko01200 |
| 5 | [Peroxisome](file:///E:\\%E7%8E%8B%E9%9B%AA%E5%B3%B0\\iTRAQ\\%E5%8D%8E%E5%A4%A7%E5%9F%BA%E5%9B%A0\\%E5%8D%8E%E5%A4%A7%E6%95%B0%E6%8D%AE\\%E8%9B%8B%E7%99%BD%E8%B4%A8%E7%BB%84%E4%B8%8E%E8%BD%AC%E5%BD%95%E7%BB%84%E5%85%B3%E8%81%94%E5%88%86%E6%9E%90\\F15FTSNCKF3616\\F15FTSNCKF3616\\Correlation\\SubCorrelation2Function\\Pathway\\BS_82-VS-BS_59_Cor_DEPs_Pathway\\BS_82-VS-BS_59_Cor_DEPs.htm#gene5#gene5) | 7 (5.07%) | ko04146 |
| 6 | [Glyoxylate and dicarboxylate metabolism](file:///E:\\%E7%8E%8B%E9%9B%AA%E5%B3%B0\\iTRAQ\\%E5%8D%8E%E5%A4%A7%E5%9F%BA%E5%9B%A0\\%E5%8D%8E%E5%A4%A7%E6%95%B0%E6%8D%AE\\%E8%9B%8B%E7%99%BD%E8%B4%A8%E7%BB%84%E4%B8%8E%E8%BD%AC%E5%BD%95%E7%BB%84%E5%85%B3%E8%81%94%E5%88%86%E6%9E%90\\F15FTSNCKF3616\\F15FTSNCKF3616\\Correlation\\SubCorrelation2Function\\Pathway\\BS_82-VS-BS_59_Cor_DEPs_Pathway\\BS_82-VS-BS_59_Cor_DEPs.htm#gene6#gene6) | 7 (5.07%) | ko00630 |
| 7 | [Phenylpropanoid biosynthesis](file:///E:\\%E7%8E%8B%E9%9B%AA%E5%B3%B0\\iTRAQ\\%E5%8D%8E%E5%A4%A7%E5%9F%BA%E5%9B%A0\\%E5%8D%8E%E5%A4%A7%E6%95%B0%E6%8D%AE\\%E8%9B%8B%E7%99%BD%E8%B4%A8%E7%BB%84%E4%B8%8E%E8%BD%AC%E5%BD%95%E7%BB%84%E5%85%B3%E8%81%94%E5%88%86%E6%9E%90\\F15FTSNCKF3616\\F15FTSNCKF3616\\Correlation\\SubCorrelation2Function\\Pathway\\BS_82-VS-BS_59_Cor_DEPs_Pathway\\BS_82-VS-BS_59_Cor_DEPs.htm#gene7#gene7) | 7 (5.07%) | ko00940 |
| 8 | [Plant-pathogen interaction](file:///E:\\%E7%8E%8B%E9%9B%AA%E5%B3%B0\\iTRAQ\\%E5%8D%8E%E5%A4%A7%E5%9F%BA%E5%9B%A0\\%E5%8D%8E%E5%A4%A7%E6%95%B0%E6%8D%AE\\%E8%9B%8B%E7%99%BD%E8%B4%A8%E7%BB%84%E4%B8%8E%E8%BD%AC%E5%BD%95%E7%BB%84%E5%85%B3%E8%81%94%E5%88%86%E6%9E%90\\F15FTSNCKF3616\\F15FTSNCKF3616\\Correlation\\SubCorrelation2Function\\Pathway\\BS_82-VS-BS_59_Cor_DEPs_Pathway\\BS_82-VS-BS_59_Cor_DEPs.htm#gene8#gene8) | 7 (5.07%) | ko04626 |
| 9 | [Endocytosis](file:///E:\\%E7%8E%8B%E9%9B%AA%E5%B3%B0\\iTRAQ\\%E5%8D%8E%E5%A4%A7%E5%9F%BA%E5%9B%A0\\%E5%8D%8E%E5%A4%A7%E6%95%B0%E6%8D%AE\\%E8%9B%8B%E7%99%BD%E8%B4%A8%E7%BB%84%E4%B8%8E%E8%BD%AC%E5%BD%95%E7%BB%84%E5%85%B3%E8%81%94%E5%88%86%E6%9E%90\\F15FTSNCKF3616\\F15FTSNCKF3616\\Correlation\\SubCorrelation2Function\\Pathway\\BS_82-VS-BS_59_Cor_DEPs_Pathway\\BS_82-VS-BS_59_Cor_DEPs.htm#gene9#gene9) | 6 (4.35%) | ko04144 |
| 10 | [RNA transport](file:///E:\\%E7%8E%8B%E9%9B%AA%E5%B3%B0\\iTRAQ\\%E5%8D%8E%E5%A4%A7%E5%9F%BA%E5%9B%A0\\%E5%8D%8E%E5%A4%A7%E6%95%B0%E6%8D%AE\\%E8%9B%8B%E7%99%BD%E8%B4%A8%E7%BB%84%E4%B8%8E%E8%BD%AC%E5%BD%95%E7%BB%84%E5%85%B3%E8%81%94%E5%88%86%E6%9E%90\\F15FTSNCKF3616\\F15FTSNCKF3616\\Correlation\\SubCorrelation2Function\\Pathway\\BS_82-VS-BS_59_Cor_DEPs_Pathway\\BS_82-VS-BS_59_Cor_DEPs.htm#gene10#gene10) | 6 (4.35%) | ko03013 |
| 11 | [Amino sugar and nucleotide sugar metabolism](file:///E:\\%E7%8E%8B%E9%9B%AA%E5%B3%B0\\iTRAQ\\%E5%8D%8E%E5%A4%A7%E5%9F%BA%E5%9B%A0\\%E5%8D%8E%E5%A4%A7%E6%95%B0%E6%8D%AE\\%E8%9B%8B%E7%99%BD%E8%B4%A8%E7%BB%84%E4%B8%8E%E8%BD%AC%E5%BD%95%E7%BB%84%E5%85%B3%E8%81%94%E5%88%86%E6%9E%90\\F15FTSNCKF3616\\F15FTSNCKF3616\\Correlation\\SubCorrelation2Function\\Pathway\\BS_82-VS-BS_59_Cor_DEPs_Pathway\\BS_82-VS-BS_59_Cor_DEPs.htm#gene11#gene11) | 5 (3.62%) | ko00520 |
| 12 | [Starch and sucrose metabolism](file:///E:\\%E7%8E%8B%E9%9B%AA%E5%B3%B0\\iTRAQ\\%E5%8D%8E%E5%A4%A7%E5%9F%BA%E5%9B%A0\\%E5%8D%8E%E5%A4%A7%E6%95%B0%E6%8D%AE\\%E8%9B%8B%E7%99%BD%E8%B4%A8%E7%BB%84%E4%B8%8E%E8%BD%AC%E5%BD%95%E7%BB%84%E5%85%B3%E8%81%94%E5%88%86%E6%9E%90\\F15FTSNCKF3616\\F15FTSNCKF3616\\Correlation\\SubCorrelation2Function\\Pathway\\BS_82-VS-BS_59_Cor_DEPs_Pathway\\BS_82-VS-BS_59_Cor_DEPs.htm#gene12#gene12) | 5 (3.62%) | ko00500 |
| 13 | [Biosynthesis of amino acids](file:///E:\\%E7%8E%8B%E9%9B%AA%E5%B3%B0\\iTRAQ\\%E5%8D%8E%E5%A4%A7%E5%9F%BA%E5%9B%A0\\%E5%8D%8E%E5%A4%A7%E6%95%B0%E6%8D%AE\\%E8%9B%8B%E7%99%BD%E8%B4%A8%E7%BB%84%E4%B8%8E%E8%BD%AC%E5%BD%95%E7%BB%84%E5%85%B3%E8%81%94%E5%88%86%E6%9E%90\\F15FTSNCKF3616\\F15FTSNCKF3616\\Correlation\\SubCorrelation2Function\\Pathway\\BS_82-VS-BS_59_Cor_DEPs_Pathway\\BS_82-VS-BS_59_Cor_DEPs.htm#gene13#gene13) | 4 (2.9%) | ko01230 |
| 14 | [Nitrogen metabolism](file:///E:\\%E7%8E%8B%E9%9B%AA%E5%B3%B0\\iTRAQ\\%E5%8D%8E%E5%A4%A7%E5%9F%BA%E5%9B%A0\\%E5%8D%8E%E5%A4%A7%E6%95%B0%E6%8D%AE\\%E8%9B%8B%E7%99%BD%E8%B4%A8%E7%BB%84%E4%B8%8E%E8%BD%AC%E5%BD%95%E7%BB%84%E5%85%B3%E8%81%94%E5%88%86%E6%9E%90\\F15FTSNCKF3616\\F15FTSNCKF3616\\Correlation\\SubCorrelation2Function\\Pathway\\BS_82-VS-BS_59_Cor_DEPs_Pathway\\BS_82-VS-BS_59_Cor_DEPs.htm#gene14#gene14) | 4 (2.9%) | ko00910 |
| 15 | [Galactose metabolism](file:///E:\\%E7%8E%8B%E9%9B%AA%E5%B3%B0\\iTRAQ\\%E5%8D%8E%E5%A4%A7%E5%9F%BA%E5%9B%A0\\%E5%8D%8E%E5%A4%A7%E6%95%B0%E6%8D%AE\\%E8%9B%8B%E7%99%BD%E8%B4%A8%E7%BB%84%E4%B8%8E%E8%BD%AC%E5%BD%95%E7%BB%84%E5%85%B3%E8%81%94%E5%88%86%E6%9E%90\\F15FTSNCKF3616\\F15FTSNCKF3616\\Correlation\\SubCorrelation2Function\\Pathway\\BS_82-VS-BS_59_Cor_DEPs_Pathway\\BS_82-VS-BS_59_Cor_DEPs.htm#gene15#gene15) | 4 (2.9%) | ko00052 |
| 16 | [Pyrimidine metabolism](file:///E:\\%E7%8E%8B%E9%9B%AA%E5%B3%B0\\iTRAQ\\%E5%8D%8E%E5%A4%A7%E5%9F%BA%E5%9B%A0\\%E5%8D%8E%E5%A4%A7%E6%95%B0%E6%8D%AE\\%E8%9B%8B%E7%99%BD%E8%B4%A8%E7%BB%84%E4%B8%8E%E8%BD%AC%E5%BD%95%E7%BB%84%E5%85%B3%E8%81%94%E5%88%86%E6%9E%90\\F15FTSNCKF3616\\F15FTSNCKF3616\\Correlation\\SubCorrelation2Function\\Pathway\\BS_82-VS-BS_59_Cor_DEPs_Pathway\\BS_82-VS-BS_59_Cor_DEPs.htm#gene16#gene16) | 4 (2.9%) | ko00240 |
| 17 | [Glycine, serine and threonine metabolism](file:///E:\\%E7%8E%8B%E9%9B%AA%E5%B3%B0\\iTRAQ\\%E5%8D%8E%E5%A4%A7%E5%9F%BA%E5%9B%A0\\%E5%8D%8E%E5%A4%A7%E6%95%B0%E6%8D%AE\\%E8%9B%8B%E7%99%BD%E8%B4%A8%E7%BB%84%E4%B8%8E%E8%BD%AC%E5%BD%95%E7%BB%84%E5%85%B3%E8%81%94%E5%88%86%E6%9E%90\\F15FTSNCKF3616\\F15FTSNCKF3616\\Correlation\\SubCorrelation2Function\\Pathway\\BS_82-VS-BS_59_Cor_DEPs_Pathway\\BS_82-VS-BS_59_Cor_DEPs.htm#gene17#gene17) | 4 (2.9%) | ko00260 |
| 18 | [Plant hormone signal transduction](file:///E:\\%E7%8E%8B%E9%9B%AA%E5%B3%B0\\iTRAQ\\%E5%8D%8E%E5%A4%A7%E5%9F%BA%E5%9B%A0\\%E5%8D%8E%E5%A4%A7%E6%95%B0%E6%8D%AE\\%E8%9B%8B%E7%99%BD%E8%B4%A8%E7%BB%84%E4%B8%8E%E8%BD%AC%E5%BD%95%E7%BB%84%E5%85%B3%E8%81%94%E5%88%86%E6%9E%90\\F15FTSNCKF3616\\F15FTSNCKF3616\\Correlation\\SubCorrelation2Function\\Pathway\\BS_82-VS-BS_59_Cor_DEPs_Pathway\\BS_82-VS-BS_59_Cor_DEPs.htm#gene18#gene18) | 3 (2.17%) | ko04075 |
| 19 | [Sphingolipid metabolism](file:///E:\\%E7%8E%8B%E9%9B%AA%E5%B3%B0\\iTRAQ\\%E5%8D%8E%E5%A4%A7%E5%9F%BA%E5%9B%A0\\%E5%8D%8E%E5%A4%A7%E6%95%B0%E6%8D%AE\\%E8%9B%8B%E7%99%BD%E8%B4%A8%E7%BB%84%E4%B8%8E%E8%BD%AC%E5%BD%95%E7%BB%84%E5%85%B3%E8%81%94%E5%88%86%E6%9E%90\\F15FTSNCKF3616\\F15FTSNCKF3616\\Correlation\\SubCorrelation2Function\\Pathway\\BS_82-VS-BS_59_Cor_DEPs_Pathway\\BS_82-VS-BS_59_Cor_DEPs.htm#gene19#gene19) | 3 (2.17%) | ko00600 |
| 20 | [Glutathione metabolism](file:///E:\\%E7%8E%8B%E9%9B%AA%E5%B3%B0\\iTRAQ\\%E5%8D%8E%E5%A4%A7%E5%9F%BA%E5%9B%A0\\%E5%8D%8E%E5%A4%A7%E6%95%B0%E6%8D%AE\\%E8%9B%8B%E7%99%BD%E8%B4%A8%E7%BB%84%E4%B8%8E%E8%BD%AC%E5%BD%95%E7%BB%84%E5%85%B3%E8%81%94%E5%88%86%E6%9E%90\\F15FTSNCKF3616\\F15FTSNCKF3616\\Correlation\\SubCorrelation2Function\\Pathway\\BS_82-VS-BS_59_Cor_DEPs_Pathway\\BS_82-VS-BS_59_Cor_DEPs.htm#gene20#gene20) | 3 (2.17%) | ko00480 |
| 21 | [Glycerophospholipid metabolism](file:///E:\\%E7%8E%8B%E9%9B%AA%E5%B3%B0\\iTRAQ\\%E5%8D%8E%E5%A4%A7%E5%9F%BA%E5%9B%A0\\%E5%8D%8E%E5%A4%A7%E6%95%B0%E6%8D%AE\\%E8%9B%8B%E7%99%BD%E8%B4%A8%E7%BB%84%E4%B8%8E%E8%BD%AC%E5%BD%95%E7%BB%84%E5%85%B3%E8%81%94%E5%88%86%E6%9E%90\\F15FTSNCKF3616\\F15FTSNCKF3616\\Correlation\\SubCorrelation2Function\\Pathway\\BS_82-VS-BS_59_Cor_DEPs_Pathway\\BS_82-VS-BS_59_Cor_DEPs.htm#gene21#gene21) | 3 (2.17%) | ko00564 |
| 22 | [Carbon fixation in photosynthetic organisms](file:///E:\\%E7%8E%8B%E9%9B%AA%E5%B3%B0\\iTRAQ\\%E5%8D%8E%E5%A4%A7%E5%9F%BA%E5%9B%A0\\%E5%8D%8E%E5%A4%A7%E6%95%B0%E6%8D%AE\\%E8%9B%8B%E7%99%BD%E8%B4%A8%E7%BB%84%E4%B8%8E%E8%BD%AC%E5%BD%95%E7%BB%84%E5%85%B3%E8%81%94%E5%88%86%E6%9E%90\\F15FTSNCKF3616\\F15FTSNCKF3616\\Correlation\\SubCorrelation2Function\\Pathway\\BS_82-VS-BS_59_Cor_DEPs_Pathway\\BS_82-VS-BS_59_Cor_DEPs.htm#gene22#gene22) | 3 (2.17%) | ko00710 |
| 23 | [Pyruvate metabolism](file:///E:\\%E7%8E%8B%E9%9B%AA%E5%B3%B0\\iTRAQ\\%E5%8D%8E%E5%A4%A7%E5%9F%BA%E5%9B%A0\\%E5%8D%8E%E5%A4%A7%E6%95%B0%E6%8D%AE\\%E8%9B%8B%E7%99%BD%E8%B4%A8%E7%BB%84%E4%B8%8E%E8%BD%AC%E5%BD%95%E7%BB%84%E5%85%B3%E8%81%94%E5%88%86%E6%9E%90\\F15FTSNCKF3616\\F15FTSNCKF3616\\Correlation\\SubCorrelation2Function\\Pathway\\BS_82-VS-BS_59_Cor_DEPs_Pathway\\BS_82-VS-BS_59_Cor_DEPs.htm#gene23#gene23) | 3 (2.17%) | ko00620 |
| 24 | [Purine metabolism](file:///E:\\%E7%8E%8B%E9%9B%AA%E5%B3%B0\\iTRAQ\\%E5%8D%8E%E5%A4%A7%E5%9F%BA%E5%9B%A0\\%E5%8D%8E%E5%A4%A7%E6%95%B0%E6%8D%AE\\%E8%9B%8B%E7%99%BD%E8%B4%A8%E7%BB%84%E4%B8%8E%E8%BD%AC%E5%BD%95%E7%BB%84%E5%85%B3%E8%81%94%E5%88%86%E6%9E%90\\F15FTSNCKF3616\\F15FTSNCKF3616\\Correlation\\SubCorrelation2Function\\Pathway\\BS_82-VS-BS_59_Cor_DEPs_Pathway\\BS_82-VS-BS_59_Cor_DEPs.htm#gene24#gene24) | 2 (1.45%) | ko00230 |
| 25 | [Flavonoid biosynthesis](file:///E:\\%E7%8E%8B%E9%9B%AA%E5%B3%B0\\iTRAQ\\%E5%8D%8E%E5%A4%A7%E5%9F%BA%E5%9B%A0\\%E5%8D%8E%E5%A4%A7%E6%95%B0%E6%8D%AE\\%E8%9B%8B%E7%99%BD%E8%B4%A8%E7%BB%84%E4%B8%8E%E8%BD%AC%E5%BD%95%E7%BB%84%E5%85%B3%E8%81%94%E5%88%86%E6%9E%90\\F15FTSNCKF3616\\F15FTSNCKF3616\\Correlation\\SubCorrelation2Function\\Pathway\\BS_82-VS-BS_59_Cor_DEPs_Pathway\\BS_82-VS-BS_59_Cor_DEPs.htm#gene25#gene25) | 2 (1.45%) | ko00941 |
| 26 | [alpha-Linolenic acid metabolism](file:///E:\\%E7%8E%8B%E9%9B%AA%E5%B3%B0\\iTRAQ\\%E5%8D%8E%E5%A4%A7%E5%9F%BA%E5%9B%A0\\%E5%8D%8E%E5%A4%A7%E6%95%B0%E6%8D%AE\\%E8%9B%8B%E7%99%BD%E8%B4%A8%E7%BB%84%E4%B8%8E%E8%BD%AC%E5%BD%95%E7%BB%84%E5%85%B3%E8%81%94%E5%88%86%E6%9E%90\\F15FTSNCKF3616\\F15FTSNCKF3616\\Correlation\\SubCorrelation2Function\\Pathway\\BS_82-VS-BS_59_Cor_DEPs_Pathway\\BS_82-VS-BS_59_Cor_DEPs.htm#gene26#gene26) | 2 (1.45%) | ko00592 |
| 27 | [Inositol phosphate metabolism](file:///E:\\%E7%8E%8B%E9%9B%AA%E5%B3%B0\\iTRAQ\\%E5%8D%8E%E5%A4%A7%E5%9F%BA%E5%9B%A0\\%E5%8D%8E%E5%A4%A7%E6%95%B0%E6%8D%AE\\%E8%9B%8B%E7%99%BD%E8%B4%A8%E7%BB%84%E4%B8%8E%E8%BD%AC%E5%BD%95%E7%BB%84%E5%85%B3%E8%81%94%E5%88%86%E6%9E%90\\F15FTSNCKF3616\\F15FTSNCKF3616\\Correlation\\SubCorrelation2Function\\Pathway\\BS_82-VS-BS_59_Cor_DEPs_Pathway\\BS_82-VS-BS_59_Cor_DEPs.htm#gene27#gene27) | 2 (1.45%) | ko00562 |
| 28 | [Arginine biosynthesis](file:///E:\\%E7%8E%8B%E9%9B%AA%E5%B3%B0\\iTRAQ\\%E5%8D%8E%E5%A4%A7%E5%9F%BA%E5%9B%A0\\%E5%8D%8E%E5%A4%A7%E6%95%B0%E6%8D%AE\\%E8%9B%8B%E7%99%BD%E8%B4%A8%E7%BB%84%E4%B8%8E%E8%BD%AC%E5%BD%95%E7%BB%84%E5%85%B3%E8%81%94%E5%88%86%E6%9E%90\\F15FTSNCKF3616\\F15FTSNCKF3616\\Correlation\\SubCorrelation2Function\\Pathway\\BS_82-VS-BS_59_Cor_DEPs_Pathway\\BS_82-VS-BS_59_Cor_DEPs.htm#gene28#gene28) | 2 (1.45%) | ko00220 |
| 29 | [Glycolysis / Gluconeogenesis](file:///E:\\%E7%8E%8B%E9%9B%AA%E5%B3%B0\\iTRAQ\\%E5%8D%8E%E5%A4%A7%E5%9F%BA%E5%9B%A0\\%E5%8D%8E%E5%A4%A7%E6%95%B0%E6%8D%AE\\%E8%9B%8B%E7%99%BD%E8%B4%A8%E7%BB%84%E4%B8%8E%E8%BD%AC%E5%BD%95%E7%BB%84%E5%85%B3%E8%81%94%E5%88%86%E6%9E%90\\F15FTSNCKF3616\\F15FTSNCKF3616\\Correlation\\SubCorrelation2Function\\Pathway\\BS_82-VS-BS_59_Cor_DEPs_Pathway\\BS_82-VS-BS_59_Cor_DEPs.htm#gene29#gene29) | 2 (1.45%) | ko00010 |
| 30 | [mRNA surveillance pathway](file:///E:\\%E7%8E%8B%E9%9B%AA%E5%B3%B0\\iTRAQ\\%E5%8D%8E%E5%A4%A7%E5%9F%BA%E5%9B%A0\\%E5%8D%8E%E5%A4%A7%E6%95%B0%E6%8D%AE\\%E8%9B%8B%E7%99%BD%E8%B4%A8%E7%BB%84%E4%B8%8E%E8%BD%AC%E5%BD%95%E7%BB%84%E5%85%B3%E8%81%94%E5%88%86%E6%9E%90\\F15FTSNCKF3616\\F15FTSNCKF3616\\Correlation\\SubCorrelation2Function\\Pathway\\BS_82-VS-BS_59_Cor_DEPs_Pathway\\BS_82-VS-BS_59_Cor_DEPs.htm#gene30#gene30) | 2 (1.45%) | ko03015 |
| 31 | [Ascorbate and aldarate metabolism](file:///E:\\%E7%8E%8B%E9%9B%AA%E5%B3%B0\\iTRAQ\\%E5%8D%8E%E5%A4%A7%E5%9F%BA%E5%9B%A0\\%E5%8D%8E%E5%A4%A7%E6%95%B0%E6%8D%AE\\%E8%9B%8B%E7%99%BD%E8%B4%A8%E7%BB%84%E4%B8%8E%E8%BD%AC%E5%BD%95%E7%BB%84%E5%85%B3%E8%81%94%E5%88%86%E6%9E%90\\F15FTSNCKF3616\\F15FTSNCKF3616\\Correlation\\SubCorrelation2Function\\Pathway\\BS_82-VS-BS_59_Cor_DEPs_Pathway\\BS_82-VS-BS_59_Cor_DEPs.htm#gene31#gene31) | 2 (1.45%) | ko00053 |
| 32 | [DNA replication](file:///E:\\%E7%8E%8B%E9%9B%AA%E5%B3%B0\\iTRAQ\\%E5%8D%8E%E5%A4%A7%E5%9F%BA%E5%9B%A0\\%E5%8D%8E%E5%A4%A7%E6%95%B0%E6%8D%AE\\%E8%9B%8B%E7%99%BD%E8%B4%A8%E7%BB%84%E4%B8%8E%E8%BD%AC%E5%BD%95%E7%BB%84%E5%85%B3%E8%81%94%E5%88%86%E6%9E%90\\F15FTSNCKF3616\\F15FTSNCKF3616\\Correlation\\SubCorrelation2Function\\Pathway\\BS_82-VS-BS_59_Cor_DEPs_Pathway\\BS_82-VS-BS_59_Cor_DEPs.htm#gene32#gene32) | 2 (1.45%) | ko03030 |
| 33 | [Alanine, aspartate and glutamate metabolism](file:///E:\\%E7%8E%8B%E9%9B%AA%E5%B3%B0\\iTRAQ\\%E5%8D%8E%E5%A4%A7%E5%9F%BA%E5%9B%A0\\%E5%8D%8E%E5%A4%A7%E6%95%B0%E6%8D%AE\\%E8%9B%8B%E7%99%BD%E8%B4%A8%E7%BB%84%E4%B8%8E%E8%BD%AC%E5%BD%95%E7%BB%84%E5%85%B3%E8%81%94%E5%88%86%E6%9E%90\\F15FTSNCKF3616\\F15FTSNCKF3616\\Correlation\\SubCorrelation2Function\\Pathway\\BS_82-VS-BS_59_Cor_DEPs_Pathway\\BS_82-VS-BS_59_Cor_DEPs.htm#gene33#gene33) | 2 (1.45%) | ko00250 |
| 34 | [Tryptophan metabolism](file:///E:\\%E7%8E%8B%E9%9B%AA%E5%B3%B0\\iTRAQ\\%E5%8D%8E%E5%A4%A7%E5%9F%BA%E5%9B%A0\\%E5%8D%8E%E5%A4%A7%E6%95%B0%E6%8D%AE\\%E8%9B%8B%E7%99%BD%E8%B4%A8%E7%BB%84%E4%B8%8E%E8%BD%AC%E5%BD%95%E7%BB%84%E5%85%B3%E8%81%94%E5%88%86%E6%9E%90\\F15FTSNCKF3616\\F15FTSNCKF3616\\Correlation\\SubCorrelation2Function\\Pathway\\BS_82-VS-BS_59_Cor_DEPs_Pathway\\BS_82-VS-BS_59_Cor_DEPs.htm#gene34#gene34) | 2 (1.45%) | ko00380 |
| 35 | [Proteasome](file:///E:\\%E7%8E%8B%E9%9B%AA%E5%B3%B0\\iTRAQ\\%E5%8D%8E%E5%A4%A7%E5%9F%BA%E5%9B%A0\\%E5%8D%8E%E5%A4%A7%E6%95%B0%E6%8D%AE\\%E8%9B%8B%E7%99%BD%E8%B4%A8%E7%BB%84%E4%B8%8E%E8%BD%AC%E5%BD%95%E7%BB%84%E5%85%B3%E8%81%94%E5%88%86%E6%9E%90\\F15FTSNCKF3616\\F15FTSNCKF3616\\Correlation\\SubCorrelation2Function\\Pathway\\BS_82-VS-BS_59_Cor_DEPs_Pathway\\BS_82-VS-BS_59_Cor_DEPs.htm#gene35#gene35) | 2 (1.45%) | ko03050 |
| 36 | [Fructose and mannose metabolism](file:///E:\\%E7%8E%8B%E9%9B%AA%E5%B3%B0\\iTRAQ\\%E5%8D%8E%E5%A4%A7%E5%9F%BA%E5%9B%A0\\%E5%8D%8E%E5%A4%A7%E6%95%B0%E6%8D%AE\\%E8%9B%8B%E7%99%BD%E8%B4%A8%E7%BB%84%E4%B8%8E%E8%BD%AC%E5%BD%95%E7%BB%84%E5%85%B3%E8%81%94%E5%88%86%E6%9E%90\\F15FTSNCKF3616\\F15FTSNCKF3616\\Correlation\\SubCorrelation2Function\\Pathway\\BS_82-VS-BS_59_Cor_DEPs_Pathway\\BS_82-VS-BS_59_Cor_DEPs.htm#gene36#gene36) | 2 (1.45%) | ko00051 |
| 37 | [Cyanoamino acid metabolism](file:///E:\\%E7%8E%8B%E9%9B%AA%E5%B3%B0\\iTRAQ\\%E5%8D%8E%E5%A4%A7%E5%9F%BA%E5%9B%A0\\%E5%8D%8E%E5%A4%A7%E6%95%B0%E6%8D%AE\\%E8%9B%8B%E7%99%BD%E8%B4%A8%E7%BB%84%E4%B8%8E%E8%BD%AC%E5%BD%95%E7%BB%84%E5%85%B3%E8%81%94%E5%88%86%E6%9E%90\\F15FTSNCKF3616\\F15FTSNCKF3616\\Correlation\\SubCorrelation2Function\\Pathway\\BS_82-VS-BS_59_Cor_DEPs_Pathway\\BS_82-VS-BS_59_Cor_DEPs.htm#gene37#gene37) | 2 (1.45%) | ko00460 |
| 38 | [Pentose and glucuronate interconversions](file:///E:\\%E7%8E%8B%E9%9B%AA%E5%B3%B0\\iTRAQ\\%E5%8D%8E%E5%A4%A7%E5%9F%BA%E5%9B%A0\\%E5%8D%8E%E5%A4%A7%E6%95%B0%E6%8D%AE\\%E8%9B%8B%E7%99%BD%E8%B4%A8%E7%BB%84%E4%B8%8E%E8%BD%AC%E5%BD%95%E7%BB%84%E5%85%B3%E8%81%94%E5%88%86%E6%9E%90\\F15FTSNCKF3616\\F15FTSNCKF3616\\Correlation\\SubCorrelation2Function\\Pathway\\BS_82-VS-BS_59_Cor_DEPs_Pathway\\BS_82-VS-BS_59_Cor_DEPs.htm#gene38#gene38) | 2 (1.45%) | ko00040 |
| 39 | [Glycosphingolipid biosynthesis - ganglio series](file:///E:\\%E7%8E%8B%E9%9B%AA%E5%B3%B0\\iTRAQ\\%E5%8D%8E%E5%A4%A7%E5%9F%BA%E5%9B%A0\\%E5%8D%8E%E5%A4%A7%E6%95%B0%E6%8D%AE\\%E8%9B%8B%E7%99%BD%E8%B4%A8%E7%BB%84%E4%B8%8E%E8%BD%AC%E5%BD%95%E7%BB%84%E5%85%B3%E8%81%94%E5%88%86%E6%9E%90\\F15FTSNCKF3616\\F15FTSNCKF3616\\Correlation\\SubCorrelation2Function\\Pathway\\BS_82-VS-BS_59_Cor_DEPs_Pathway\\BS_82-VS-BS_59_Cor_DEPs.htm#gene39#gene39) | 1 (0.72%) | ko00604 |
| 40 | [ABC transporters](file:///E:\\%E7%8E%8B%E9%9B%AA%E5%B3%B0\\iTRAQ\\%E5%8D%8E%E5%A4%A7%E5%9F%BA%E5%9B%A0\\%E5%8D%8E%E5%A4%A7%E6%95%B0%E6%8D%AE\\%E8%9B%8B%E7%99%BD%E8%B4%A8%E7%BB%84%E4%B8%8E%E8%BD%AC%E5%BD%95%E7%BB%84%E5%85%B3%E8%81%94%E5%88%86%E6%9E%90\\F15FTSNCKF3616\\F15FTSNCKF3616\\Correlation\\SubCorrelation2Function\\Pathway\\BS_82-VS-BS_59_Cor_DEPs_Pathway\\BS_82-VS-BS_59_Cor_DEPs.htm#gene40#gene40) | 1 (0.72%) | ko02010 |
| 41 | [Linoleic acid metabolism](file:///E:\\%E7%8E%8B%E9%9B%AA%E5%B3%B0\\iTRAQ\\%E5%8D%8E%E5%A4%A7%E5%9F%BA%E5%9B%A0\\%E5%8D%8E%E5%A4%A7%E6%95%B0%E6%8D%AE\\%E8%9B%8B%E7%99%BD%E8%B4%A8%E7%BB%84%E4%B8%8E%E8%BD%AC%E5%BD%95%E7%BB%84%E5%85%B3%E8%81%94%E5%88%86%E6%9E%90\\F15FTSNCKF3616\\F15FTSNCKF3616\\Correlation\\SubCorrelation2Function\\Pathway\\BS_82-VS-BS_59_Cor_DEPs_Pathway\\BS_82-VS-BS_59_Cor_DEPs.htm#gene41#gene41) | 1 (0.72%) | ko00591 |
| 42 | [Lysine degradation](file:///E:\\%E7%8E%8B%E9%9B%AA%E5%B3%B0\\iTRAQ\\%E5%8D%8E%E5%A4%A7%E5%9F%BA%E5%9B%A0\\%E5%8D%8E%E5%A4%A7%E6%95%B0%E6%8D%AE\\%E8%9B%8B%E7%99%BD%E8%B4%A8%E7%BB%84%E4%B8%8E%E8%BD%AC%E5%BD%95%E7%BB%84%E5%85%B3%E8%81%94%E5%88%86%E6%9E%90\\F15FTSNCKF3616\\F15FTSNCKF3616\\Correlation\\SubCorrelation2Function\\Pathway\\BS_82-VS-BS_59_Cor_DEPs_Pathway\\BS_82-VS-BS_59_Cor_DEPs.htm#gene42#gene42) | 1 (0.72%) | ko00310 |
| 43 | [Porphyrin and chlorophyll metabolism](file:///E:\\%E7%8E%8B%E9%9B%AA%E5%B3%B0\\iTRAQ\\%E5%8D%8E%E5%A4%A7%E5%9F%BA%E5%9B%A0\\%E5%8D%8E%E5%A4%A7%E6%95%B0%E6%8D%AE\\%E8%9B%8B%E7%99%BD%E8%B4%A8%E7%BB%84%E4%B8%8E%E8%BD%AC%E5%BD%95%E7%BB%84%E5%85%B3%E8%81%94%E5%88%86%E6%9E%90\\F15FTSNCKF3616\\F15FTSNCKF3616\\Correlation\\SubCorrelation2Function\\Pathway\\BS_82-VS-BS_59_Cor_DEPs_Pathway\\BS_82-VS-BS_59_Cor_DEPs.htm#gene43#gene43) | 1 (0.72%) | ko00860 |
| 44 | [Tropane, piperidine and pyridine alkaloid biosynthesis](file:///E:\\%E7%8E%8B%E9%9B%AA%E5%B3%B0\\iTRAQ\\%E5%8D%8E%E5%A4%A7%E5%9F%BA%E5%9B%A0\\%E5%8D%8E%E5%A4%A7%E6%95%B0%E6%8D%AE\\%E8%9B%8B%E7%99%BD%E8%B4%A8%E7%BB%84%E4%B8%8E%E8%BD%AC%E5%BD%95%E7%BB%84%E5%85%B3%E8%81%94%E5%88%86%E6%9E%90\\F15FTSNCKF3616\\F15FTSNCKF3616\\Correlation\\SubCorrelation2Function\\Pathway\\BS_82-VS-BS_59_Cor_DEPs_Pathway\\BS_82-VS-BS_59_Cor_DEPs.htm#gene44#gene44) | 1 (0.72%) | ko00960 |
| 45 | [Betalain biosynthesis](file:///E:\\%E7%8E%8B%E9%9B%AA%E5%B3%B0\\iTRAQ\\%E5%8D%8E%E5%A4%A7%E5%9F%BA%E5%9B%A0\\%E5%8D%8E%E5%A4%A7%E6%95%B0%E6%8D%AE\\%E8%9B%8B%E7%99%BD%E8%B4%A8%E7%BB%84%E4%B8%8E%E8%BD%AC%E5%BD%95%E7%BB%84%E5%85%B3%E8%81%94%E5%88%86%E6%9E%90\\F15FTSNCKF3616\\F15FTSNCKF3616\\Correlation\\SubCorrelation2Function\\Pathway\\BS_82-VS-BS_59_Cor_DEPs_Pathway\\BS_82-VS-BS_59_Cor_DEPs.htm#gene45#gene45) | 1 (0.72%) | ko00965 |
| 46 | [Carotenoid biosynthesis](file:///E:\\%E7%8E%8B%E9%9B%AA%E5%B3%B0\\iTRAQ\\%E5%8D%8E%E5%A4%A7%E5%9F%BA%E5%9B%A0\\%E5%8D%8E%E5%A4%A7%E6%95%B0%E6%8D%AE\\%E8%9B%8B%E7%99%BD%E8%B4%A8%E7%BB%84%E4%B8%8E%E8%BD%AC%E5%BD%95%E7%BB%84%E5%85%B3%E8%81%94%E5%88%86%E6%9E%90\\F15FTSNCKF3616\\F15FTSNCKF3616\\Correlation\\SubCorrelation2Function\\Pathway\\BS_82-VS-BS_59_Cor_DEPs_Pathway\\BS_82-VS-BS_59_Cor_DEPs.htm#gene46#gene46) | 1 (0.72%) | ko00906 |
| 47 | [N-Glycan biosynthesis](file:///E:\\%E7%8E%8B%E9%9B%AA%E5%B3%B0\\iTRAQ\\%E5%8D%8E%E5%A4%A7%E5%9F%BA%E5%9B%A0\\%E5%8D%8E%E5%A4%A7%E6%95%B0%E6%8D%AE\\%E8%9B%8B%E7%99%BD%E8%B4%A8%E7%BB%84%E4%B8%8E%E8%BD%AC%E5%BD%95%E7%BB%84%E5%85%B3%E8%81%94%E5%88%86%E6%9E%90\\F15FTSNCKF3616\\F15FTSNCKF3616\\Correlation\\SubCorrelation2Function\\Pathway\\BS_82-VS-BS_59_Cor_DEPs_Pathway\\BS_82-VS-BS_59_Cor_DEPs.htm#gene47#gene47) | 1 (0.72%) | ko00510 |
| 48 | [Terpenoid backbone biosynthesis](file:///E:\\%E7%8E%8B%E9%9B%AA%E5%B3%B0\\iTRAQ\\%E5%8D%8E%E5%A4%A7%E5%9F%BA%E5%9B%A0\\%E5%8D%8E%E5%A4%A7%E6%95%B0%E6%8D%AE\\%E8%9B%8B%E7%99%BD%E8%B4%A8%E7%BB%84%E4%B8%8E%E8%BD%AC%E5%BD%95%E7%BB%84%E5%85%B3%E8%81%94%E5%88%86%E6%9E%90\\F15FTSNCKF3616\\F15FTSNCKF3616\\Correlation\\SubCorrelation2Function\\Pathway\\BS_82-VS-BS_59_Cor_DEPs_Pathway\\BS_82-VS-BS_59_Cor_DEPs.htm#gene48#gene48) | 1 (0.72%) | ko00900 |
| 49 | [Protein export](file:///E:\\%E7%8E%8B%E9%9B%AA%E5%B3%B0\\iTRAQ\\%E5%8D%8E%E5%A4%A7%E5%9F%BA%E5%9B%A0\\%E5%8D%8E%E5%A4%A7%E6%95%B0%E6%8D%AE\\%E8%9B%8B%E7%99%BD%E8%B4%A8%E7%BB%84%E4%B8%8E%E8%BD%AC%E5%BD%95%E7%BB%84%E5%85%B3%E8%81%94%E5%88%86%E6%9E%90\\F15FTSNCKF3616\\F15FTSNCKF3616\\Correlation\\SubCorrelation2Function\\Pathway\\BS_82-VS-BS_59_Cor_DEPs_Pathway\\BS_82-VS-BS_59_Cor_DEPs.htm#gene49#gene49) | 1 (0.72%) | ko03060 |
| 50 | [2-Oxocarboxylic acid metabolism](file:///E:\\%E7%8E%8B%E9%9B%AA%E5%B3%B0\\iTRAQ\\%E5%8D%8E%E5%A4%A7%E5%9F%BA%E5%9B%A0\\%E5%8D%8E%E5%A4%A7%E6%95%B0%E6%8D%AE\\%E8%9B%8B%E7%99%BD%E8%B4%A8%E7%BB%84%E4%B8%8E%E8%BD%AC%E5%BD%95%E7%BB%84%E5%85%B3%E8%81%94%E5%88%86%E6%9E%90\\F15FTSNCKF3616\\F15FTSNCKF3616\\Correlation\\SubCorrelation2Function\\Pathway\\BS_82-VS-BS_59_Cor_DEPs_Pathway\\BS_82-VS-BS_59_Cor_DEPs.htm#gene50#gene50) | 1 (0.72%) | ko01210 |
| 51 | [Glycosaminoglycan degradation](file:///E:\\%E7%8E%8B%E9%9B%AA%E5%B3%B0\\iTRAQ\\%E5%8D%8E%E5%A4%A7%E5%9F%BA%E5%9B%A0\\%E5%8D%8E%E5%A4%A7%E6%95%B0%E6%8D%AE\\%E8%9B%8B%E7%99%BD%E8%B4%A8%E7%BB%84%E4%B8%8E%E8%BD%AC%E5%BD%95%E7%BB%84%E5%85%B3%E8%81%94%E5%88%86%E6%9E%90\\F15FTSNCKF3616\\F15FTSNCKF3616\\Correlation\\SubCorrelation2Function\\Pathway\\BS_82-VS-BS_59_Cor_DEPs_Pathway\\BS_82-VS-BS_59_Cor_DEPs.htm#gene51#gene51) | 1 (0.72%) | ko00531 |
| 52 | [Cysteine and methionine metabolism](file:///E:\\%E7%8E%8B%E9%9B%AA%E5%B3%B0\\iTRAQ\\%E5%8D%8E%E5%A4%A7%E5%9F%BA%E5%9B%A0\\%E5%8D%8E%E5%A4%A7%E6%95%B0%E6%8D%AE\\%E8%9B%8B%E7%99%BD%E8%B4%A8%E7%BB%84%E4%B8%8E%E8%BD%AC%E5%BD%95%E7%BB%84%E5%85%B3%E8%81%94%E5%88%86%E6%9E%90\\F15FTSNCKF3616\\F15FTSNCKF3616\\Correlation\\SubCorrelation2Function\\Pathway\\BS_82-VS-BS_59_Cor_DEPs_Pathway\\BS_82-VS-BS_59_Cor_DEPs.htm#gene52#gene52) | 1 (0.72%) | ko00270 |
| 53 | [Other glycan degradation](file:///E:\\%E7%8E%8B%E9%9B%AA%E5%B3%B0\\iTRAQ\\%E5%8D%8E%E5%A4%A7%E5%9F%BA%E5%9B%A0\\%E5%8D%8E%E5%A4%A7%E6%95%B0%E6%8D%AE\\%E8%9B%8B%E7%99%BD%E8%B4%A8%E7%BB%84%E4%B8%8E%E8%BD%AC%E5%BD%95%E7%BB%84%E5%85%B3%E8%81%94%E5%88%86%E6%9E%90\\F15FTSNCKF3616\\F15FTSNCKF3616\\Correlation\\SubCorrelation2Function\\Pathway\\BS_82-VS-BS_59_Cor_DEPs_Pathway\\BS_82-VS-BS_59_Cor_DEPs.htm#gene53#gene53) | 1 (0.72%) | ko00511 |
| 54 | [Spliceosome](file:///E:\\%E7%8E%8B%E9%9B%AA%E5%B3%B0\\iTRAQ\\%E5%8D%8E%E5%A4%A7%E5%9F%BA%E5%9B%A0\\%E5%8D%8E%E5%A4%A7%E6%95%B0%E6%8D%AE\\%E8%9B%8B%E7%99%BD%E8%B4%A8%E7%BB%84%E4%B8%8E%E8%BD%AC%E5%BD%95%E7%BB%84%E5%85%B3%E8%81%94%E5%88%86%E6%9E%90\\F15FTSNCKF3616\\F15FTSNCKF3616\\Correlation\\SubCorrelation2Function\\Pathway\\BS_82-VS-BS_59_Cor_DEPs_Pathway\\BS_82-VS-BS_59_Cor_DEPs.htm#gene54#gene54) | 1 (0.72%) | ko03040 |
| 55 | [Fatty acid elongation](file:///E:\\%E7%8E%8B%E9%9B%AA%E5%B3%B0\\iTRAQ\\%E5%8D%8E%E5%A4%A7%E5%9F%BA%E5%9B%A0\\%E5%8D%8E%E5%A4%A7%E6%95%B0%E6%8D%AE\\%E8%9B%8B%E7%99%BD%E8%B4%A8%E7%BB%84%E4%B8%8E%E8%BD%AC%E5%BD%95%E7%BB%84%E5%85%B3%E8%81%94%E5%88%86%E6%9E%90\\F15FTSNCKF3616\\F15FTSNCKF3616\\Correlation\\SubCorrelation2Function\\Pathway\\BS_82-VS-BS_59_Cor_DEPs_Pathway\\BS_82-VS-BS_59_Cor_DEPs.htm#gene55#gene55) | 1 (0.72%) | ko00062 |
| 56 | [Isoquinoline alkaloid biosynthesis](file:///E:\\%E7%8E%8B%E9%9B%AA%E5%B3%B0\\iTRAQ\\%E5%8D%8E%E5%A4%A7%E5%9F%BA%E5%9B%A0\\%E5%8D%8E%E5%A4%A7%E6%95%B0%E6%8D%AE\\%E8%9B%8B%E7%99%BD%E8%B4%A8%E7%BB%84%E4%B8%8E%E8%BD%AC%E5%BD%95%E7%BB%84%E5%85%B3%E8%81%94%E5%88%86%E6%9E%90\\F15FTSNCKF3616\\F15FTSNCKF3616\\Correlation\\SubCorrelation2Function\\Pathway\\BS_82-VS-BS_59_Cor_DEPs_Pathway\\BS_82-VS-BS_59_Cor_DEPs.htm#gene56#gene56) | 1 (0.72%) | ko00950 |
| 57 | [Stilbenoid, diarylheptanoid and gingerol biosynthesis](file:///E:\\%E7%8E%8B%E9%9B%AA%E5%B3%B0\\iTRAQ\\%E5%8D%8E%E5%A4%A7%E5%9F%BA%E5%9B%A0\\%E5%8D%8E%E5%A4%A7%E6%95%B0%E6%8D%AE\\%E8%9B%8B%E7%99%BD%E8%B4%A8%E7%BB%84%E4%B8%8E%E8%BD%AC%E5%BD%95%E7%BB%84%E5%85%B3%E8%81%94%E5%88%86%E6%9E%90\\F15FTSNCKF3616\\F15FTSNCKF3616\\Correlation\\SubCorrelation2Function\\Pathway\\BS_82-VS-BS_59_Cor_DEPs_Pathway\\BS_82-VS-BS_59_Cor_DEPs.htm#gene57#gene57) | 1 (0.72%) | ko00945 |
| 58 | [Citrate cycle (TCA cycle)](file:///E:\\%E7%8E%8B%E9%9B%AA%E5%B3%B0\\iTRAQ\\%E5%8D%8E%E5%A4%A7%E5%9F%BA%E5%9B%A0\\%E5%8D%8E%E5%A4%A7%E6%95%B0%E6%8D%AE\\%E8%9B%8B%E7%99%BD%E8%B4%A8%E7%BB%84%E4%B8%8E%E8%BD%AC%E5%BD%95%E7%BB%84%E5%85%B3%E8%81%94%E5%88%86%E6%9E%90\\F15FTSNCKF3616\\F15FTSNCKF3616\\Correlation\\SubCorrelation2Function\\Pathway\\BS_82-VS-BS_59_Cor_DEPs_Pathway\\BS_82-VS-BS_59_Cor_DEPs.htm#gene58#gene58) | 1 (0.72%) | ko00020 |
| 59 | [Phenylalanine metabolism](file:///E:\\%E7%8E%8B%E9%9B%AA%E5%B3%B0\\iTRAQ\\%E5%8D%8E%E5%A4%A7%E5%9F%BA%E5%9B%A0\\%E5%8D%8E%E5%A4%A7%E6%95%B0%E6%8D%AE\\%E8%9B%8B%E7%99%BD%E8%B4%A8%E7%BB%84%E4%B8%8E%E8%BD%AC%E5%BD%95%E7%BB%84%E5%85%B3%E8%81%94%E5%88%86%E6%9E%90\\F15FTSNCKF3616\\F15FTSNCKF3616\\Correlation\\SubCorrelation2Function\\Pathway\\BS_82-VS-BS_59_Cor_DEPs_Pathway\\BS_82-VS-BS_59_Cor_DEPs.htm#gene59#gene59) | 1 (0.72%) | ko00360 |
| 60 | [Glycerolipid metabolism](file:///E:\\%E7%8E%8B%E9%9B%AA%E5%B3%B0\\iTRAQ\\%E5%8D%8E%E5%A4%A7%E5%9F%BA%E5%9B%A0\\%E5%8D%8E%E5%A4%A7%E6%95%B0%E6%8D%AE\\%E8%9B%8B%E7%99%BD%E8%B4%A8%E7%BB%84%E4%B8%8E%E8%BD%AC%E5%BD%95%E7%BB%84%E5%85%B3%E8%81%94%E5%88%86%E6%9E%90\\F15FTSNCKF3616\\F15FTSNCKF3616\\Correlation\\SubCorrelation2Function\\Pathway\\BS_82-VS-BS_59_Cor_DEPs_Pathway\\BS_82-VS-BS_59_Cor_DEPs.htm#gene60#gene60) | 1 (0.72%) | ko00561 |
| 61 | [Ubiquinone and other terpenoid-quinone biosynthesis](file:///E:\\%E7%8E%8B%E9%9B%AA%E5%B3%B0\\iTRAQ\\%E5%8D%8E%E5%A4%A7%E5%9F%BA%E5%9B%A0\\%E5%8D%8E%E5%A4%A7%E6%95%B0%E6%8D%AE\\%E8%9B%8B%E7%99%BD%E8%B4%A8%E7%BB%84%E4%B8%8E%E8%BD%AC%E5%BD%95%E7%BB%84%E5%85%B3%E8%81%94%E5%88%86%E6%9E%90\\F15FTSNCKF3616\\F15FTSNCKF3616\\Correlation\\SubCorrelation2Function\\Pathway\\BS_82-VS-BS_59_Cor_DEPs_Pathway\\BS_82-VS-BS_59_Cor_DEPs.htm#gene61#gene61) | 1 (0.72%) | ko00130 |
| 62 | [Steroid biosynthesis](file:///E:\\%E7%8E%8B%E9%9B%AA%E5%B3%B0\\iTRAQ\\%E5%8D%8E%E5%A4%A7%E5%9F%BA%E5%9B%A0\\%E5%8D%8E%E5%A4%A7%E6%95%B0%E6%8D%AE\\%E8%9B%8B%E7%99%BD%E8%B4%A8%E7%BB%84%E4%B8%8E%E8%BD%AC%E5%BD%95%E7%BB%84%E5%85%B3%E8%81%94%E5%88%86%E6%9E%90\\F15FTSNCKF3616\\F15FTSNCKF3616\\Correlation\\SubCorrelation2Function\\Pathway\\BS_82-VS-BS_59_Cor_DEPs_Pathway\\BS_82-VS-BS_59_Cor_DEPs.htm#gene62#gene62) | 1 (0.72%) | ko00100 |
| 63 | [Nicotinate and nicotinamide metabolism](file:///E:\\%E7%8E%8B%E9%9B%AA%E5%B3%B0\\iTRAQ\\%E5%8D%8E%E5%A4%A7%E5%9F%BA%E5%9B%A0\\%E5%8D%8E%E5%A4%A7%E6%95%B0%E6%8D%AE\\%E8%9B%8B%E7%99%BD%E8%B4%A8%E7%BB%84%E4%B8%8E%E8%BD%AC%E5%BD%95%E7%BB%84%E5%85%B3%E8%81%94%E5%88%86%E6%9E%90\\F15FTSNCKF3616\\F15FTSNCKF3616\\Correlation\\SubCorrelation2Function\\Pathway\\BS_82-VS-BS_59_Cor_DEPs_Pathway\\BS_82-VS-BS_59_Cor_DEPs.htm#gene63#gene63) | 1 (0.72%) | ko00760 |
| 64 | [Cutin, suberine and wax biosynthesis](file:///E:\\%E7%8E%8B%E9%9B%AA%E5%B3%B0\\iTRAQ\\%E5%8D%8E%E5%A4%A7%E5%9F%BA%E5%9B%A0\\%E5%8D%8E%E5%A4%A7%E6%95%B0%E6%8D%AE\\%E8%9B%8B%E7%99%BD%E8%B4%A8%E7%BB%84%E4%B8%8E%E8%BD%AC%E5%BD%95%E7%BB%84%E5%85%B3%E8%81%94%E5%88%86%E6%9E%90\\F15FTSNCKF3616\\F15FTSNCKF3616\\Correlation\\SubCorrelation2Function\\Pathway\\BS_82-VS-BS_59_Cor_DEPs_Pathway\\BS_82-VS-BS_59_Cor_DEPs.htm#gene64#gene64) | 1 (0.72%) | ko00073 |
| 65 | [RNA polymerase](file:///E:\\%E7%8E%8B%E9%9B%AA%E5%B3%B0\\iTRAQ\\%E5%8D%8E%E5%A4%A7%E5%9F%BA%E5%9B%A0\\%E5%8D%8E%E5%A4%A7%E6%95%B0%E6%8D%AE\\%E8%9B%8B%E7%99%BD%E8%B4%A8%E7%BB%84%E4%B8%8E%E8%BD%AC%E5%BD%95%E7%BB%84%E5%85%B3%E8%81%94%E5%88%86%E6%9E%90\\F15FTSNCKF3616\\F15FTSNCKF3616\\Correlation\\SubCorrelation2Function\\Pathway\\BS_82-VS-BS_59_Cor_DEPs_Pathway\\BS_82-VS-BS_59_Cor_DEPs.htm#gene65#gene65) | 1 (0.72%) | ko03020 |
| 66 | [Valine, leucine and isoleucine degradation](file:///E:\\%E7%8E%8B%E9%9B%AA%E5%B3%B0\\iTRAQ\\%E5%8D%8E%E5%A4%A7%E5%9F%BA%E5%9B%A0\\%E5%8D%8E%E5%A4%A7%E6%95%B0%E6%8D%AE\\%E8%9B%8B%E7%99%BD%E8%B4%A8%E7%BB%84%E4%B8%8E%E8%BD%AC%E5%BD%95%E7%BB%84%E5%85%B3%E8%81%94%E5%88%86%E6%9E%90\\F15FTSNCKF3616\\F15FTSNCKF3616\\Correlation\\SubCorrelation2Function\\Pathway\\BS_82-VS-BS_59_Cor_DEPs_Pathway\\BS_82-VS-BS_59_Cor_DEPs.htm#gene66#gene66) | 1 (0.72%) | ko00280 |
| 67 | [Fatty acid metabolism](file:///E:\\%E7%8E%8B%E9%9B%AA%E5%B3%B0\\iTRAQ\\%E5%8D%8E%E5%A4%A7%E5%9F%BA%E5%9B%A0\\%E5%8D%8E%E5%A4%A7%E6%95%B0%E6%8D%AE\\%E8%9B%8B%E7%99%BD%E8%B4%A8%E7%BB%84%E4%B8%8E%E8%BD%AC%E5%BD%95%E7%BB%84%E5%85%B3%E8%81%94%E5%88%86%E6%9E%90\\F15FTSNCKF3616\\F15FTSNCKF3616\\Correlation\\SubCorrelation2Function\\Pathway\\BS_82-VS-BS_59_Cor_DEPs_Pathway\\BS_82-VS-BS_59_Cor_DEPs.htm#gene67#gene67) | 1 (0.72%) | ko01212 |
| 68 | [beta-Alanine metabolism](file:///E:\\%E7%8E%8B%E9%9B%AA%E5%B3%B0\\iTRAQ\\%E5%8D%8E%E5%A4%A7%E5%9F%BA%E5%9B%A0\\%E5%8D%8E%E5%A4%A7%E6%95%B0%E6%8D%AE\\%E8%9B%8B%E7%99%BD%E8%B4%A8%E7%BB%84%E4%B8%8E%E8%BD%AC%E5%BD%95%E7%BB%84%E5%85%B3%E8%81%94%E5%88%86%E6%9E%90\\F15FTSNCKF3616\\F15FTSNCKF3616\\Correlation\\SubCorrelation2Function\\Pathway\\BS_82-VS-BS_59_Cor_DEPs_Pathway\\BS_82-VS-BS_59_Cor_DEPs.htm#gene68#gene68) | 1 (0.72%) | ko00410 |
| 69 | [Glycosphingolipid biosynthesis - globo series](file:///E:\\%E7%8E%8B%E9%9B%AA%E5%B3%B0\\iTRAQ\\%E5%8D%8E%E5%A4%A7%E5%9F%BA%E5%9B%A0\\%E5%8D%8E%E5%A4%A7%E6%95%B0%E6%8D%AE\\%E8%9B%8B%E7%99%BD%E8%B4%A8%E7%BB%84%E4%B8%8E%E8%BD%AC%E5%BD%95%E7%BB%84%E5%85%B3%E8%81%94%E5%88%86%E6%9E%90\\F15FTSNCKF3616\\F15FTSNCKF3616\\Correlation\\SubCorrelation2Function\\Pathway\\BS_82-VS-BS_59_Cor_DEPs_Pathway\\BS_82-VS-BS_59_Cor_DEPs.htm#gene69#gene69) | 1 (0.72%) | ko00603 |
| 70 | [Phagosome](file:///E:\\%E7%8E%8B%E9%9B%AA%E5%B3%B0\\iTRAQ\\%E5%8D%8E%E5%A4%A7%E5%9F%BA%E5%9B%A0\\%E5%8D%8E%E5%A4%A7%E6%95%B0%E6%8D%AE\\%E8%9B%8B%E7%99%BD%E8%B4%A8%E7%BB%84%E4%B8%8E%E8%BD%AC%E5%BD%95%E7%BB%84%E5%85%B3%E8%81%94%E5%88%86%E6%9E%90\\F15FTSNCKF3616\\F15FTSNCKF3616\\Correlation\\SubCorrelation2Function\\Pathway\\BS_82-VS-BS_59_Cor_DEPs_Pathway\\BS_82-VS-BS_59_Cor_DEPs.htm#gene70#gene70) | 1 (0.72%) | ko04145 |
| 71 | [Ubiquitin mediated proteolysis](file:///E:\\%E7%8E%8B%E9%9B%AA%E5%B3%B0\\iTRAQ\\%E5%8D%8E%E5%A4%A7%E5%9F%BA%E5%9B%A0\\%E5%8D%8E%E5%A4%A7%E6%95%B0%E6%8D%AE\\%E8%9B%8B%E7%99%BD%E8%B4%A8%E7%BB%84%E4%B8%8E%E8%BD%AC%E5%BD%95%E7%BB%84%E5%85%B3%E8%81%94%E5%88%86%E6%9E%90\\F15FTSNCKF3616\\F15FTSNCKF3616\\Correlation\\SubCorrelation2Function\\Pathway\\BS_82-VS-BS_59_Cor_DEPs_Pathway\\BS_82-VS-BS_59_Cor_DEPs.htm#gene71#gene71) | 1 (0.72%) | ko04120 |
| 72 | [Tyrosine metabolism](file:///E:\\%E7%8E%8B%E9%9B%AA%E5%B3%B0\\iTRAQ\\%E5%8D%8E%E5%A4%A7%E5%9F%BA%E5%9B%A0\\%E5%8D%8E%E5%A4%A7%E6%95%B0%E6%8D%AE\\%E8%9B%8B%E7%99%BD%E8%B4%A8%E7%BB%84%E4%B8%8E%E8%BD%AC%E5%BD%95%E7%BB%84%E5%85%B3%E8%81%94%E5%88%86%E6%9E%90\\F15FTSNCKF3616\\F15FTSNCKF3616\\Correlation\\SubCorrelation2Function\\Pathway\\BS_82-VS-BS_59_Cor_DEPs_Pathway\\BS_82-VS-BS_59_Cor_DEPs.htm#gene72#gene72) | 1 (0.72%) | ko00350 |
| **SD59-VS-BS59_Cor_DEGs_DEPs** | | | |
| # | Pathway | Diff Proteins with pathway annotation (51) | Pathway ID |
| 1 | [Metabolic pathways](file:///E:\\%E7%8E%8B%E9%9B%AA%E5%B3%B0\\iTRAQ\\%E5%8D%8E%E5%A4%A7%E5%9F%BA%E5%9B%A0\\%E5%8D%8E%E5%A4%A7%E6%95%B0%E6%8D%AE\\%E8%9B%8B%E7%99%BD%E8%B4%A8%E7%BB%84%E4%B8%8E%E8%BD%AC%E5%BD%95%E7%BB%84%E5%85%B3%E8%81%94%E5%88%86%E6%9E%90\\F15FTSNCKF3616\\F15FTSNCKF3616\\Correlation\\SubCorrelation2Function\\Pathway\\SD_59-VS-BS_59_Cor_DEPs_Pathway\\SD_59-VS-BS_59_Cor_DEPs.htm#gene1#gene1) | 23 (45.1%) | ko01100 |
| 2 | [Biosynthesis of secondary metabolites](file:///E:\\%E7%8E%8B%E9%9B%AA%E5%B3%B0\\iTRAQ\\%E5%8D%8E%E5%A4%A7%E5%9F%BA%E5%9B%A0\\%E5%8D%8E%E5%A4%A7%E6%95%B0%E6%8D%AE\\%E8%9B%8B%E7%99%BD%E8%B4%A8%E7%BB%84%E4%B8%8E%E8%BD%AC%E5%BD%95%E7%BB%84%E5%85%B3%E8%81%94%E5%88%86%E6%9E%90\\F15FTSNCKF3616\\F15FTSNCKF3616\\Correlation\\SubCorrelation2Function\\Pathway\\SD_59-VS-BS_59_Cor_DEPs_Pathway\\SD_59-VS-BS_59_Cor_DEPs.htm#gene2#gene2) | 16 (31.37%) | ko01110 |
| 3 | [Plant-pathogen interaction](file:///E:\\%E7%8E%8B%E9%9B%AA%E5%B3%B0\\iTRAQ\\%E5%8D%8E%E5%A4%A7%E5%9F%BA%E5%9B%A0\\%E5%8D%8E%E5%A4%A7%E6%95%B0%E6%8D%AE\\%E8%9B%8B%E7%99%BD%E8%B4%A8%E7%BB%84%E4%B8%8E%E8%BD%AC%E5%BD%95%E7%BB%84%E5%85%B3%E8%81%94%E5%88%86%E6%9E%90\\F15FTSNCKF3616\\F15FTSNCKF3616\\Correlation\\SubCorrelation2Function\\Pathway\\SD_59-VS-BS_59_Cor_DEPs_Pathway\\SD_59-VS-BS_59_Cor_DEPs.htm#gene3#gene3) | 6 (11.76%) | ko04626 |
| 4 | [Phenylpropanoid biosynthesis](file:///E:\\%E7%8E%8B%E9%9B%AA%E5%B3%B0\\iTRAQ\\%E5%8D%8E%E5%A4%A7%E5%9F%BA%E5%9B%A0\\%E5%8D%8E%E5%A4%A7%E6%95%B0%E6%8D%AE\\%E8%9B%8B%E7%99%BD%E8%B4%A8%E7%BB%84%E4%B8%8E%E8%BD%AC%E5%BD%95%E7%BB%84%E5%85%B3%E8%81%94%E5%88%86%E6%9E%90\\F15FTSNCKF3616\\F15FTSNCKF3616\\Correlation\\SubCorrelation2Function\\Pathway\\SD_59-VS-BS_59_Cor_DEPs_Pathway\\SD_59-VS-BS_59_Cor_DEPs.htm#gene4#gene4) | 6 (11.76%) | ko00940 |
| 5 | [Biosynthesis of amino acids](file:///E:\\%E7%8E%8B%E9%9B%AA%E5%B3%B0\\iTRAQ\\%E5%8D%8E%E5%A4%A7%E5%9F%BA%E5%9B%A0\\%E5%8D%8E%E5%A4%A7%E6%95%B0%E6%8D%AE\\%E8%9B%8B%E7%99%BD%E8%B4%A8%E7%BB%84%E4%B8%8E%E8%BD%AC%E5%BD%95%E7%BB%84%E5%85%B3%E8%81%94%E5%88%86%E6%9E%90\\F15FTSNCKF3616\\F15FTSNCKF3616\\Correlation\\SubCorrelation2Function\\Pathway\\SD_59-VS-BS_59_Cor_DEPs_Pathway\\SD_59-VS-BS_59_Cor_DEPs.htm#gene5#gene5) | 5 (9.8%) | ko01230 |
| 6 | [Starch and sucrose metabolism](file:///E:\\%E7%8E%8B%E9%9B%AA%E5%B3%B0\\iTRAQ\\%E5%8D%8E%E5%A4%A7%E5%9F%BA%E5%9B%A0\\%E5%8D%8E%E5%A4%A7%E6%95%B0%E6%8D%AE\\%E8%9B%8B%E7%99%BD%E8%B4%A8%E7%BB%84%E4%B8%8E%E8%BD%AC%E5%BD%95%E7%BB%84%E5%85%B3%E8%81%94%E5%88%86%E6%9E%90\\F15FTSNCKF3616\\F15FTSNCKF3616\\Correlation\\SubCorrelation2Function\\Pathway\\SD_59-VS-BS_59_Cor_DEPs_Pathway\\SD_59-VS-BS_59_Cor_DEPs.htm#gene6#gene6) | 5 (9.8%) | ko00500 |
| 7 | [Pentose and glucuronate interconversions](file:///E:\\%E7%8E%8B%E9%9B%AA%E5%B3%B0\\iTRAQ\\%E5%8D%8E%E5%A4%A7%E5%9F%BA%E5%9B%A0\\%E5%8D%8E%E5%A4%A7%E6%95%B0%E6%8D%AE\\%E8%9B%8B%E7%99%BD%E8%B4%A8%E7%BB%84%E4%B8%8E%E8%BD%AC%E5%BD%95%E7%BB%84%E5%85%B3%E8%81%94%E5%88%86%E6%9E%90\\F15FTSNCKF3616\\F15FTSNCKF3616\\Correlation\\SubCorrelation2Function\\Pathway\\SD_59-VS-BS_59_Cor_DEPs_Pathway\\SD_59-VS-BS_59_Cor_DEPs.htm#gene7#gene7) | 4 (7.84%) | ko00040 |
| 8 | [Ascorbate and aldarate metabolism](file:///E:\\%E7%8E%8B%E9%9B%AA%E5%B3%B0\\iTRAQ\\%E5%8D%8E%E5%A4%A7%E5%9F%BA%E5%9B%A0\\%E5%8D%8E%E5%A4%A7%E6%95%B0%E6%8D%AE\\%E8%9B%8B%E7%99%BD%E8%B4%A8%E7%BB%84%E4%B8%8E%E8%BD%AC%E5%BD%95%E7%BB%84%E5%85%B3%E8%81%94%E5%88%86%E6%9E%90\\F15FTSNCKF3616\\F15FTSNCKF3616\\Correlation\\SubCorrelation2Function\\Pathway\\SD_59-VS-BS_59_Cor_DEPs_Pathway\\SD_59-VS-BS_59_Cor_DEPs.htm#gene8#gene8) | 4 (7.84%) | ko00053 |
| 9 | [Carbon metabolism](file:///E:\\%E7%8E%8B%E9%9B%AA%E5%B3%B0\\iTRAQ\\%E5%8D%8E%E5%A4%A7%E5%9F%BA%E5%9B%A0\\%E5%8D%8E%E5%A4%A7%E6%95%B0%E6%8D%AE\\%E8%9B%8B%E7%99%BD%E8%B4%A8%E7%BB%84%E4%B8%8E%E8%BD%AC%E5%BD%95%E7%BB%84%E5%85%B3%E8%81%94%E5%88%86%E6%9E%90\\F15FTSNCKF3616\\F15FTSNCKF3616\\Correlation\\SubCorrelation2Function\\Pathway\\SD_59-VS-BS_59_Cor_DEPs_Pathway\\SD_59-VS-BS_59_Cor_DEPs.htm#gene9#gene9) | 4 (7.84%) | ko01200 |
| 10 | [Glutathione metabolism](file:///E:\\%E7%8E%8B%E9%9B%AA%E5%B3%B0\\iTRAQ\\%E5%8D%8E%E5%A4%A7%E5%9F%BA%E5%9B%A0\\%E5%8D%8E%E5%A4%A7%E6%95%B0%E6%8D%AE\\%E8%9B%8B%E7%99%BD%E8%B4%A8%E7%BB%84%E4%B8%8E%E8%BD%AC%E5%BD%95%E7%BB%84%E5%85%B3%E8%81%94%E5%88%86%E6%9E%90\\F15FTSNCKF3616\\F15FTSNCKF3616\\Correlation\\SubCorrelation2Function\\Pathway\\SD_59-VS-BS_59_Cor_DEPs_Pathway\\SD_59-VS-BS_59_Cor_DEPs.htm#gene10#gene10) | 3 (5.88%) | ko00480 |
| 11 | [Phenylalanine metabolism](file:///E:\\%E7%8E%8B%E9%9B%AA%E5%B3%B0\\iTRAQ\\%E5%8D%8E%E5%A4%A7%E5%9F%BA%E5%9B%A0\\%E5%8D%8E%E5%A4%A7%E6%95%B0%E6%8D%AE\\%E8%9B%8B%E7%99%BD%E8%B4%A8%E7%BB%84%E4%B8%8E%E8%BD%AC%E5%BD%95%E7%BB%84%E5%85%B3%E8%81%94%E5%88%86%E6%9E%90\\F15FTSNCKF3616\\F15FTSNCKF3616\\Correlation\\SubCorrelation2Function\\Pathway\\SD_59-VS-BS_59_Cor_DEPs_Pathway\\SD_59-VS-BS_59_Cor_DEPs.htm#gene11#gene11) | 3 (5.88%) | ko00360 |
| 12 | [Cyanoamino acid metabolism](file:///E:\\%E7%8E%8B%E9%9B%AA%E5%B3%B0\\iTRAQ\\%E5%8D%8E%E5%A4%A7%E5%9F%BA%E5%9B%A0\\%E5%8D%8E%E5%A4%A7%E6%95%B0%E6%8D%AE\\%E8%9B%8B%E7%99%BD%E8%B4%A8%E7%BB%84%E4%B8%8E%E8%BD%AC%E5%BD%95%E7%BB%84%E5%85%B3%E8%81%94%E5%88%86%E6%9E%90\\F15FTSNCKF3616\\F15FTSNCKF3616\\Correlation\\SubCorrelation2Function\\Pathway\\SD_59-VS-BS_59_Cor_DEPs_Pathway\\SD_59-VS-BS_59_Cor_DEPs.htm#gene12#gene12) | 3 (5.88%) | ko00460 |
| 13 | [Cysteine and methionine metabolism](file:///E:\\%E7%8E%8B%E9%9B%AA%E5%B3%B0\\iTRAQ\\%E5%8D%8E%E5%A4%A7%E5%9F%BA%E5%9B%A0\\%E5%8D%8E%E5%A4%A7%E6%95%B0%E6%8D%AE\\%E8%9B%8B%E7%99%BD%E8%B4%A8%E7%BB%84%E4%B8%8E%E8%BD%AC%E5%BD%95%E7%BB%84%E5%85%B3%E8%81%94%E5%88%86%E6%9E%90\\F15FTSNCKF3616\\F15FTSNCKF3616\\Correlation\\SubCorrelation2Function\\Pathway\\SD_59-VS-BS_59_Cor_DEPs_Pathway\\SD_59-VS-BS_59_Cor_DEPs.htm#gene13#gene13) | 3 (5.88%) | ko00270 |
| 14 | [Phenylalanine, tyrosine and tryptophan biosynthesis](file:///E:\\%E7%8E%8B%E9%9B%AA%E5%B3%B0\\iTRAQ\\%E5%8D%8E%E5%A4%A7%E5%9F%BA%E5%9B%A0\\%E5%8D%8E%E5%A4%A7%E6%95%B0%E6%8D%AE\\%E8%9B%8B%E7%99%BD%E8%B4%A8%E7%BB%84%E4%B8%8E%E8%BD%AC%E5%BD%95%E7%BB%84%E5%85%B3%E8%81%94%E5%88%86%E6%9E%90\\F15FTSNCKF3616\\F15FTSNCKF3616\\Correlation\\SubCorrelation2Function\\Pathway\\SD_59-VS-BS_59_Cor_DEPs_Pathway\\SD_59-VS-BS_59_Cor_DEPs.htm#gene14#gene14) | 3 (5.88%) | ko00400 |
| 15 | [Isoquinoline alkaloid biosynthesis](file:///E:\\%E7%8E%8B%E9%9B%AA%E5%B3%B0\\iTRAQ\\%E5%8D%8E%E5%A4%A7%E5%9F%BA%E5%9B%A0\\%E5%8D%8E%E5%A4%A7%E6%95%B0%E6%8D%AE\\%E8%9B%8B%E7%99%BD%E8%B4%A8%E7%BB%84%E4%B8%8E%E8%BD%AC%E5%BD%95%E7%BB%84%E5%85%B3%E8%81%94%E5%88%86%E6%9E%90\\F15FTSNCKF3616\\F15FTSNCKF3616\\Correlation\\SubCorrelation2Function\\Pathway\\SD_59-VS-BS_59_Cor_DEPs_Pathway\\SD_59-VS-BS_59_Cor_DEPs.htm#gene15#gene15) | 2 (3.92%) | ko00950 |
| 16 | [Peroxisome](file:///E:\\%E7%8E%8B%E9%9B%AA%E5%B3%B0\\iTRAQ\\%E5%8D%8E%E5%A4%A7%E5%9F%BA%E5%9B%A0\\%E5%8D%8E%E5%A4%A7%E6%95%B0%E6%8D%AE\\%E8%9B%8B%E7%99%BD%E8%B4%A8%E7%BB%84%E4%B8%8E%E8%BD%AC%E5%BD%95%E7%BB%84%E5%85%B3%E8%81%94%E5%88%86%E6%9E%90\\F15FTSNCKF3616\\F15FTSNCKF3616\\Correlation\\SubCorrelation2Function\\Pathway\\SD_59-VS-BS_59_Cor_DEPs_Pathway\\SD_59-VS-BS_59_Cor_DEPs.htm#gene16#gene16) | 2 (3.92%) | ko04146 |
| 17 | [Amino sugar and nucleotide sugar metabolism](file:///E:\\%E7%8E%8B%E9%9B%AA%E5%B3%B0\\iTRAQ\\%E5%8D%8E%E5%A4%A7%E5%9F%BA%E5%9B%A0\\%E5%8D%8E%E5%A4%A7%E6%95%B0%E6%8D%AE\\%E8%9B%8B%E7%99%BD%E8%B4%A8%E7%BB%84%E4%B8%8E%E8%BD%AC%E5%BD%95%E7%BB%84%E5%85%B3%E8%81%94%E5%88%86%E6%9E%90\\F15FTSNCKF3616\\F15FTSNCKF3616\\Correlation\\SubCorrelation2Function\\Pathway\\SD_59-VS-BS_59_Cor_DEPs_Pathway\\SD_59-VS-BS_59_Cor_DEPs.htm#gene17#gene17) | 2 (3.92%) | ko00520 |
| 18 | [Glyoxylate and dicarboxylate metabolism](file:///E:\\%E7%8E%8B%E9%9B%AA%E5%B3%B0\\iTRAQ\\%E5%8D%8E%E5%A4%A7%E5%9F%BA%E5%9B%A0\\%E5%8D%8E%E5%A4%A7%E6%95%B0%E6%8D%AE\\%E8%9B%8B%E7%99%BD%E8%B4%A8%E7%BB%84%E4%B8%8E%E8%BD%AC%E5%BD%95%E7%BB%84%E5%85%B3%E8%81%94%E5%88%86%E6%9E%90\\F15FTSNCKF3616\\F15FTSNCKF3616\\Correlation\\SubCorrelation2Function\\Pathway\\SD_59-VS-BS_59_Cor_DEPs_Pathway\\SD_59-VS-BS_59_Cor_DEPs.htm#gene18#gene18) | 2 (3.92%) | ko00630 |
| 19 | [Galactose metabolism](file:///E:\\%E7%8E%8B%E9%9B%AA%E5%B3%B0\\iTRAQ\\%E5%8D%8E%E5%A4%A7%E5%9F%BA%E5%9B%A0\\%E5%8D%8E%E5%A4%A7%E6%95%B0%E6%8D%AE\\%E8%9B%8B%E7%99%BD%E8%B4%A8%E7%BB%84%E4%B8%8E%E8%BD%AC%E5%BD%95%E7%BB%84%E5%85%B3%E8%81%94%E5%88%86%E6%9E%90\\F15FTSNCKF3616\\F15FTSNCKF3616\\Correlation\\SubCorrelation2Function\\Pathway\\SD_59-VS-BS_59_Cor_DEPs_Pathway\\SD_59-VS-BS_59_Cor_DEPs.htm#gene19#gene19) | 2 (3.92%) | ko00052 |
| 20 | [Ubiquinone and other terpenoid-quinone biosynthesis](file:///E:\\%E7%8E%8B%E9%9B%AA%E5%B3%B0\\iTRAQ\\%E5%8D%8E%E5%A4%A7%E5%9F%BA%E5%9B%A0\\%E5%8D%8E%E5%A4%A7%E6%95%B0%E6%8D%AE\\%E8%9B%8B%E7%99%BD%E8%B4%A8%E7%BB%84%E4%B8%8E%E8%BD%AC%E5%BD%95%E7%BB%84%E5%85%B3%E8%81%94%E5%88%86%E6%9E%90\\F15FTSNCKF3616\\F15FTSNCKF3616\\Correlation\\SubCorrelation2Function\\Pathway\\SD_59-VS-BS_59_Cor_DEPs_Pathway\\SD_59-VS-BS_59_Cor_DEPs.htm#gene20#gene20) | 2 (3.92%) | ko00130 |
| 21 | [Tropane, piperidine and pyridine alkaloid biosynthesis](file:///E:\\%E7%8E%8B%E9%9B%AA%E5%B3%B0\\iTRAQ\\%E5%8D%8E%E5%A4%A7%E5%9F%BA%E5%9B%A0\\%E5%8D%8E%E5%A4%A7%E6%95%B0%E6%8D%AE\\%E8%9B%8B%E7%99%BD%E8%B4%A8%E7%BB%84%E4%B8%8E%E8%BD%AC%E5%BD%95%E7%BB%84%E5%85%B3%E8%81%94%E5%88%86%E6%9E%90\\F15FTSNCKF3616\\F15FTSNCKF3616\\Correlation\\SubCorrelation2Function\\Pathway\\SD_59-VS-BS_59_Cor_DEPs_Pathway\\SD_59-VS-BS_59_Cor_DEPs.htm#gene21#gene21) | 2 (3.92%) | ko00960 |
| 22 | [Fructose and mannose metabolism](file:///E:\\%E7%8E%8B%E9%9B%AA%E5%B3%B0\\iTRAQ\\%E5%8D%8E%E5%A4%A7%E5%9F%BA%E5%9B%A0\\%E5%8D%8E%E5%A4%A7%E6%95%B0%E6%8D%AE\\%E8%9B%8B%E7%99%BD%E8%B4%A8%E7%BB%84%E4%B8%8E%E8%BD%AC%E5%BD%95%E7%BB%84%E5%85%B3%E8%81%94%E5%88%86%E6%9E%90\\F15FTSNCKF3616\\F15FTSNCKF3616\\Correlation\\SubCorrelation2Function\\Pathway\\SD_59-VS-BS_59_Cor_DEPs_Pathway\\SD_59-VS-BS_59_Cor_DEPs.htm#gene22#gene22) | 2 (3.92%) | ko00051 |
| 23 | [Other glycan degradation](file:///E:\\%E7%8E%8B%E9%9B%AA%E5%B3%B0\\iTRAQ\\%E5%8D%8E%E5%A4%A7%E5%9F%BA%E5%9B%A0\\%E5%8D%8E%E5%A4%A7%E6%95%B0%E6%8D%AE\\%E8%9B%8B%E7%99%BD%E8%B4%A8%E7%BB%84%E4%B8%8E%E8%BD%AC%E5%BD%95%E7%BB%84%E5%85%B3%E8%81%94%E5%88%86%E6%9E%90\\F15FTSNCKF3616\\F15FTSNCKF3616\\Correlation\\SubCorrelation2Function\\Pathway\\SD_59-VS-BS_59_Cor_DEPs_Pathway\\SD_59-VS-BS_59_Cor_DEPs.htm#gene23#gene23) | 2 (3.92%) | ko00511 |
| 24 | [Tyrosine metabolism](file:///E:\\%E7%8E%8B%E9%9B%AA%E5%B3%B0\\iTRAQ\\%E5%8D%8E%E5%A4%A7%E5%9F%BA%E5%9B%A0\\%E5%8D%8E%E5%A4%A7%E6%95%B0%E6%8D%AE\\%E8%9B%8B%E7%99%BD%E8%B4%A8%E7%BB%84%E4%B8%8E%E8%BD%AC%E5%BD%95%E7%BB%84%E5%85%B3%E8%81%94%E5%88%86%E6%9E%90\\F15FTSNCKF3616\\F15FTSNCKF3616\\Correlation\\SubCorrelation2Function\\Pathway\\SD_59-VS-BS_59_Cor_DEPs_Pathway\\SD_59-VS-BS_59_Cor_DEPs.htm#gene24#gene24) | 2 (3.92%) | ko00350 |
| 25 | [Sphingolipid metabolism](file:///E:\\%E7%8E%8B%E9%9B%AA%E5%B3%B0\\iTRAQ\\%E5%8D%8E%E5%A4%A7%E5%9F%BA%E5%9B%A0\\%E5%8D%8E%E5%A4%A7%E6%95%B0%E6%8D%AE\\%E8%9B%8B%E7%99%BD%E8%B4%A8%E7%BB%84%E4%B8%8E%E8%BD%AC%E5%BD%95%E7%BB%84%E5%85%B3%E8%81%94%E5%88%86%E6%9E%90\\F15FTSNCKF3616\\F15FTSNCKF3616\\Correlation\\SubCorrelation2Function\\Pathway\\SD_59-VS-BS_59_Cor_DEPs_Pathway\\SD_59-VS-BS_59_Cor_DEPs.htm#gene25#gene25) | 1 (1.96%) | ko00600 |
| 26 | [Arginine and proline metabolism](file:///E:\\%E7%8E%8B%E9%9B%AA%E5%B3%B0\\iTRAQ\\%E5%8D%8E%E5%A4%A7%E5%9F%BA%E5%9B%A0\\%E5%8D%8E%E5%A4%A7%E6%95%B0%E6%8D%AE\\%E8%9B%8B%E7%99%BD%E8%B4%A8%E7%BB%84%E4%B8%8E%E8%BD%AC%E5%BD%95%E7%BB%84%E5%85%B3%E8%81%94%E5%88%86%E6%9E%90\\F15FTSNCKF3616\\F15FTSNCKF3616\\Correlation\\SubCorrelation2Function\\Pathway\\SD_59-VS-BS_59_Cor_DEPs_Pathway\\SD_59-VS-BS_59_Cor_DEPs.htm#gene26#gene26) | 1 (1.96%) | ko00330 |
| 27 | [Plant hormone signal transduction](file:///E:\\%E7%8E%8B%E9%9B%AA%E5%B3%B0\\iTRAQ\\%E5%8D%8E%E5%A4%A7%E5%9F%BA%E5%9B%A0\\%E5%8D%8E%E5%A4%A7%E6%95%B0%E6%8D%AE\\%E8%9B%8B%E7%99%BD%E8%B4%A8%E7%BB%84%E4%B8%8E%E8%BD%AC%E5%BD%95%E7%BB%84%E5%85%B3%E8%81%94%E5%88%86%E6%9E%90\\F15FTSNCKF3616\\F15FTSNCKF3616\\Correlation\\SubCorrelation2Function\\Pathway\\SD_59-VS-BS_59_Cor_DEPs_Pathway\\SD_59-VS-BS_59_Cor_DEPs.htm#gene27#gene27) | 1 (1.96%) | ko04075 |
| 28 | [Tryptophan metabolism](file:///E:\\%E7%8E%8B%E9%9B%AA%E5%B3%B0\\iTRAQ\\%E5%8D%8E%E5%A4%A7%E5%9F%BA%E5%9B%A0\\%E5%8D%8E%E5%A4%A7%E6%95%B0%E6%8D%AE\\%E8%9B%8B%E7%99%BD%E8%B4%A8%E7%BB%84%E4%B8%8E%E8%BD%AC%E5%BD%95%E7%BB%84%E5%85%B3%E8%81%94%E5%88%86%E6%9E%90\\F15FTSNCKF3616\\F15FTSNCKF3616\\Correlation\\SubCorrelation2Function\\Pathway\\SD_59-VS-BS_59_Cor_DEPs_Pathway\\SD_59-VS-BS_59_Cor_DEPs.htm#gene28#gene28) | 1 (1.96%) | ko00380 |
| 29 | [Stilbenoid, diarylheptanoid and gingerol biosynthesis](file:///E:\\%E7%8E%8B%E9%9B%AA%E5%B3%B0\\iTRAQ\\%E5%8D%8E%E5%A4%A7%E5%9F%BA%E5%9B%A0\\%E5%8D%8E%E5%A4%A7%E6%95%B0%E6%8D%AE\\%E8%9B%8B%E7%99%BD%E8%B4%A8%E7%BB%84%E4%B8%8E%E8%BD%AC%E5%BD%95%E7%BB%84%E5%85%B3%E8%81%94%E5%88%86%E6%9E%90\\F15FTSNCKF3616\\F15FTSNCKF3616\\Correlation\\SubCorrelation2Function\\Pathway\\SD_59-VS-BS_59_Cor_DEPs_Pathway\\SD_59-VS-BS_59_Cor_DEPs.htm#gene29#gene29) | 1 (1.96%) | ko00945 |
| 30 | [Glycerolipid metabolism](file:///E:\\%E7%8E%8B%E9%9B%AA%E5%B3%B0\\iTRAQ\\%E5%8D%8E%E5%A4%A7%E5%9F%BA%E5%9B%A0\\%E5%8D%8E%E5%A4%A7%E6%95%B0%E6%8D%AE\\%E8%9B%8B%E7%99%BD%E8%B4%A8%E7%BB%84%E4%B8%8E%E8%BD%AC%E5%BD%95%E7%BB%84%E5%85%B3%E8%81%94%E5%88%86%E6%9E%90\\F15FTSNCKF3616\\F15FTSNCKF3616\\Correlation\\SubCorrelation2Function\\Pathway\\SD_59-VS-BS_59_Cor_DEPs_Pathway\\SD_59-VS-BS_59_Cor_DEPs.htm#gene30#gene30) | 1 (1.96%) | ko00561 |
| 31 | [Limonene and pinene degradation](file:///E:\\%E7%8E%8B%E9%9B%AA%E5%B3%B0\\iTRAQ\\%E5%8D%8E%E5%A4%A7%E5%9F%BA%E5%9B%A0\\%E5%8D%8E%E5%A4%A7%E6%95%B0%E6%8D%AE\\%E8%9B%8B%E7%99%BD%E8%B4%A8%E7%BB%84%E4%B8%8E%E8%BD%AC%E5%BD%95%E7%BB%84%E5%85%B3%E8%81%94%E5%88%86%E6%9E%90\\F15FTSNCKF3616\\F15FTSNCKF3616\\Correlation\\SubCorrelation2Function\\Pathway\\SD_59-VS-BS_59_Cor_DEPs_Pathway\\SD_59-VS-BS_59_Cor_DEPs.htm#gene31#gene31) | 1 (1.96%) | ko00903 |
| 32 | [Cutin, suberine and wax biosynthesis](file:///E:\\%E7%8E%8B%E9%9B%AA%E5%B3%B0\\iTRAQ\\%E5%8D%8E%E5%A4%A7%E5%9F%BA%E5%9B%A0\\%E5%8D%8E%E5%A4%A7%E6%95%B0%E6%8D%AE\\%E8%9B%8B%E7%99%BD%E8%B4%A8%E7%BB%84%E4%B8%8E%E8%BD%AC%E5%BD%95%E7%BB%84%E5%85%B3%E8%81%94%E5%88%86%E6%9E%90\\F15FTSNCKF3616\\F15FTSNCKF3616\\Correlation\\SubCorrelation2Function\\Pathway\\SD_59-VS-BS_59_Cor_DEPs_Pathway\\SD_59-VS-BS_59_Cor_DEPs.htm#gene32#gene32) | 1 (1.96%) | ko00073 |
| 33 | [Protein processing in endoplasmic reticulum](file:///E:\\%E7%8E%8B%E9%9B%AA%E5%B3%B0\\iTRAQ\\%E5%8D%8E%E5%A4%A7%E5%9F%BA%E5%9B%A0\\%E5%8D%8E%E5%A4%A7%E6%95%B0%E6%8D%AE\\%E8%9B%8B%E7%99%BD%E8%B4%A8%E7%BB%84%E4%B8%8E%E8%BD%AC%E5%BD%95%E7%BB%84%E5%85%B3%E8%81%94%E5%88%86%E6%9E%90\\F15FTSNCKF3616\\F15FTSNCKF3616\\Correlation\\SubCorrelation2Function\\Pathway\\SD_59-VS-BS_59_Cor_DEPs_Pathway\\SD_59-VS-BS_59_Cor_DEPs.htm#gene33#gene33) | 1 (1.96%) | ko04141 |
| 34 | [Diterpenoid biosynthesis](file:///E:\\%E7%8E%8B%E9%9B%AA%E5%B3%B0\\iTRAQ\\%E5%8D%8E%E5%A4%A7%E5%9F%BA%E5%9B%A0\\%E5%8D%8E%E5%A4%A7%E6%95%B0%E6%8D%AE\\%E8%9B%8B%E7%99%BD%E8%B4%A8%E7%BB%84%E4%B8%8E%E8%BD%AC%E5%BD%95%E7%BB%84%E5%85%B3%E8%81%94%E5%88%86%E6%9E%90\\F15FTSNCKF3616\\F15FTSNCKF3616\\Correlation\\SubCorrelation2Function\\Pathway\\SD_59-VS-BS_59_Cor_DEPs_Pathway\\SD_59-VS-BS_59_Cor_DEPs.htm#gene34#gene34) | 1 (1.96%) | ko00904 |
| 35 | [Sulfur metabolism](file:///E:\\%E7%8E%8B%E9%9B%AA%E5%B3%B0\\iTRAQ\\%E5%8D%8E%E5%A4%A7%E5%9F%BA%E5%9B%A0\\%E5%8D%8E%E5%A4%A7%E6%95%B0%E6%8D%AE\\%E8%9B%8B%E7%99%BD%E8%B4%A8%E7%BB%84%E4%B8%8E%E8%BD%AC%E5%BD%95%E7%BB%84%E5%85%B3%E8%81%94%E5%88%86%E6%9E%90\\F15FTSNCKF3616\\F15FTSNCKF3616\\Correlation\\SubCorrelation2Function\\Pathway\\SD_59-VS-BS_59_Cor_DEPs_Pathway\\SD_59-VS-BS_59_Cor_DEPs.htm#gene35#gene35) | 1 (1.96%) | ko00920 |
| 36 | [Monoterpenoid biosynthesis](file:///E:\\%E7%8E%8B%E9%9B%AA%E5%B3%B0\\iTRAQ\\%E5%8D%8E%E5%A4%A7%E5%9F%BA%E5%9B%A0\\%E5%8D%8E%E5%A4%A7%E6%95%B0%E6%8D%AE\\%E8%9B%8B%E7%99%BD%E8%B4%A8%E7%BB%84%E4%B8%8E%E8%BD%AC%E5%BD%95%E7%BB%84%E5%85%B3%E8%81%94%E5%88%86%E6%9E%90\\F15FTSNCKF3616\\F15FTSNCKF3616\\Correlation\\SubCorrelation2Function\\Pathway\\SD_59-VS-BS_59_Cor_DEPs_Pathway\\SD_59-VS-BS_59_Cor_DEPs.htm#gene36#gene36) | 1 (1.96%) | ko00902 |
| 37 | [mRNA surveillance pathway](file:///E:\\%E7%8E%8B%E9%9B%AA%E5%B3%B0\\iTRAQ\\%E5%8D%8E%E5%A4%A7%E5%9F%BA%E5%9B%A0\\%E5%8D%8E%E5%A4%A7%E6%95%B0%E6%8D%AE\\%E8%9B%8B%E7%99%BD%E8%B4%A8%E7%BB%84%E4%B8%8E%E8%BD%AC%E5%BD%95%E7%BB%84%E5%85%B3%E8%81%94%E5%88%86%E6%9E%90\\F15FTSNCKF3616\\F15FTSNCKF3616\\Correlation\\SubCorrelation2Function\\Pathway\\SD_59-VS-BS_59_Cor_DEPs_Pathway\\SD_59-VS-BS_59_Cor_DEPs.htm#gene37#gene37) | 1 (1.96%) | ko03015 |
| 38 | [Glycine, serine and threonine metabolism](file:///E:\\%E7%8E%8B%E9%9B%AA%E5%B3%B0\\iTRAQ\\%E5%8D%8E%E5%A4%A7%E5%9F%BA%E5%9B%A0\\%E5%8D%8E%E5%A4%A7%E6%95%B0%E6%8D%AE\\%E8%9B%8B%E7%99%BD%E8%B4%A8%E7%BB%84%E4%B8%8E%E8%BD%AC%E5%BD%95%E7%BB%84%E5%85%B3%E8%81%94%E5%88%86%E6%9E%90\\F15FTSNCKF3616\\F15FTSNCKF3616\\Correlation\\SubCorrelation2Function\\Pathway\\SD_59-VS-BS_59_Cor_DEPs_Pathway\\SD_59-VS-BS_59_Cor_DEPs.htm#gene38#gene38) | 1 (1.96%) | ko00260 |
| **SD82-VS-BS82_Cor_DEGs_DEPs** | | | |
| # | Pathway | Diff Proteins with pathway annotation (112) | Pathway ID |
| 1 | [Metabolic pathways](file:///E:\\%E7%8E%8B%E9%9B%AA%E5%B3%B0\\iTRAQ\\%E5%8D%8E%E5%A4%A7%E5%9F%BA%E5%9B%A0\\%E5%8D%8E%E5%A4%A7%E6%95%B0%E6%8D%AE\\%E8%9B%8B%E7%99%BD%E8%B4%A8%E7%BB%84%E4%B8%8E%E8%BD%AC%E5%BD%95%E7%BB%84%E5%85%B3%E8%81%94%E5%88%86%E6%9E%90\\F15FTSNCKF3616\\F15FTSNCKF3616\\Correlation\\SubCorrelation2Function\\Pathway\\SD_82-VS-BS_82_Cor_DEPs_Pathway\\SD_82-VS-BS_82_Cor_DEPs.htm#gene1#gene1) | 48 (42.86%) | ko01100 |
| 2 | [Biosynthesis of secondary metabolites](file:///E:\\%E7%8E%8B%E9%9B%AA%E5%B3%B0\\iTRAQ\\%E5%8D%8E%E5%A4%A7%E5%9F%BA%E5%9B%A0\\%E5%8D%8E%E5%A4%A7%E6%95%B0%E6%8D%AE\\%E8%9B%8B%E7%99%BD%E8%B4%A8%E7%BB%84%E4%B8%8E%E8%BD%AC%E5%BD%95%E7%BB%84%E5%85%B3%E8%81%94%E5%88%86%E6%9E%90\\F15FTSNCKF3616\\F15FTSNCKF3616\\Correlation\\SubCorrelation2Function\\Pathway\\SD_82-VS-BS_82_Cor_DEPs_Pathway\\SD_82-VS-BS_82_Cor_DEPs.htm#gene2#gene2) | 30 (26.79%) | ko01110 |
| 3 | [Phenylpropanoid biosynthesis](file:///E:\\%E7%8E%8B%E9%9B%AA%E5%B3%B0\\iTRAQ\\%E5%8D%8E%E5%A4%A7%E5%9F%BA%E5%9B%A0\\%E5%8D%8E%E5%A4%A7%E6%95%B0%E6%8D%AE\\%E8%9B%8B%E7%99%BD%E8%B4%A8%E7%BB%84%E4%B8%8E%E8%BD%AC%E5%BD%95%E7%BB%84%E5%85%B3%E8%81%94%E5%88%86%E6%9E%90\\F15FTSNCKF3616\\F15FTSNCKF3616\\Correlation\\SubCorrelation2Function\\Pathway\\SD_82-VS-BS_82_Cor_DEPs_Pathway\\SD_82-VS-BS_82_Cor_DEPs.htm#gene3#gene3) | 11 (9.82%) | ko00940 |
| 4 | [Amino sugar and nucleotide sugar metabolism](file:///E:\\%E7%8E%8B%E9%9B%AA%E5%B3%B0\\iTRAQ\\%E5%8D%8E%E5%A4%A7%E5%9F%BA%E5%9B%A0\\%E5%8D%8E%E5%A4%A7%E6%95%B0%E6%8D%AE\\%E8%9B%8B%E7%99%BD%E8%B4%A8%E7%BB%84%E4%B8%8E%E8%BD%AC%E5%BD%95%E7%BB%84%E5%85%B3%E8%81%94%E5%88%86%E6%9E%90\\F15FTSNCKF3616\\F15FTSNCKF3616\\Correlation\\SubCorrelation2Function\\Pathway\\SD_82-VS-BS_82_Cor_DEPs_Pathway\\SD_82-VS-BS_82_Cor_DEPs.htm#gene4#gene4) | 8 (7.14%) | ko00520 |
| 5 | [Starch and sucrose metabolism](file:///E:\\%E7%8E%8B%E9%9B%AA%E5%B3%B0\\iTRAQ\\%E5%8D%8E%E5%A4%A7%E5%9F%BA%E5%9B%A0\\%E5%8D%8E%E5%A4%A7%E6%95%B0%E6%8D%AE\\%E8%9B%8B%E7%99%BD%E8%B4%A8%E7%BB%84%E4%B8%8E%E8%BD%AC%E5%BD%95%E7%BB%84%E5%85%B3%E8%81%94%E5%88%86%E6%9E%90\\F15FTSNCKF3616\\F15FTSNCKF3616\\Correlation\\SubCorrelation2Function\\Pathway\\SD_82-VS-BS_82_Cor_DEPs_Pathway\\SD_82-VS-BS_82_Cor_DEPs.htm#gene5#gene5) | 7 (6.25%) | ko00500 |
| 6 | [Plant hormone signal transduction](file:///E:\\%E7%8E%8B%E9%9B%AA%E5%B3%B0\\iTRAQ\\%E5%8D%8E%E5%A4%A7%E5%9F%BA%E5%9B%A0\\%E5%8D%8E%E5%A4%A7%E6%95%B0%E6%8D%AE\\%E8%9B%8B%E7%99%BD%E8%B4%A8%E7%BB%84%E4%B8%8E%E8%BD%AC%E5%BD%95%E7%BB%84%E5%85%B3%E8%81%94%E5%88%86%E6%9E%90\\F15FTSNCKF3616\\F15FTSNCKF3616\\Correlation\\SubCorrelation2Function\\Pathway\\SD_82-VS-BS_82_Cor_DEPs_Pathway\\SD_82-VS-BS_82_Cor_DEPs.htm#gene6#gene6) | 5 (4.46%) | ko04075 |
| 7 | [Glutathione metabolism](file:///E:\\%E7%8E%8B%E9%9B%AA%E5%B3%B0\\iTRAQ\\%E5%8D%8E%E5%A4%A7%E5%9F%BA%E5%9B%A0\\%E5%8D%8E%E5%A4%A7%E6%95%B0%E6%8D%AE\\%E8%9B%8B%E7%99%BD%E8%B4%A8%E7%BB%84%E4%B8%8E%E8%BD%AC%E5%BD%95%E7%BB%84%E5%85%B3%E8%81%94%E5%88%86%E6%9E%90\\F15FTSNCKF3616\\F15FTSNCKF3616\\Correlation\\SubCorrelation2Function\\Pathway\\SD_82-VS-BS_82_Cor_DEPs_Pathway\\SD_82-VS-BS_82_Cor_DEPs.htm#gene7#gene7) | 5 (4.46%) | ko00480 |
| 8 | [Ribosome](file:///E:\\%E7%8E%8B%E9%9B%AA%E5%B3%B0\\iTRAQ\\%E5%8D%8E%E5%A4%A7%E5%9F%BA%E5%9B%A0\\%E5%8D%8E%E5%A4%A7%E6%95%B0%E6%8D%AE\\%E8%9B%8B%E7%99%BD%E8%B4%A8%E7%BB%84%E4%B8%8E%E8%BD%AC%E5%BD%95%E7%BB%84%E5%85%B3%E8%81%94%E5%88%86%E6%9E%90\\F15FTSNCKF3616\\F15FTSNCKF3616\\Correlation\\SubCorrelation2Function\\Pathway\\SD_82-VS-BS_82_Cor_DEPs_Pathway\\SD_82-VS-BS_82_Cor_DEPs.htm#gene8#gene8) | 5 (4.46%) | ko03010 |
| 9 | [Plant-pathogen interaction](file:///E:\\%E7%8E%8B%E9%9B%AA%E5%B3%B0\\iTRAQ\\%E5%8D%8E%E5%A4%A7%E5%9F%BA%E5%9B%A0\\%E5%8D%8E%E5%A4%A7%E6%95%B0%E6%8D%AE\\%E8%9B%8B%E7%99%BD%E8%B4%A8%E7%BB%84%E4%B8%8E%E8%BD%AC%E5%BD%95%E7%BB%84%E5%85%B3%E8%81%94%E5%88%86%E6%9E%90\\F15FTSNCKF3616\\F15FTSNCKF3616\\Correlation\\SubCorrelation2Function\\Pathway\\SD_82-VS-BS_82_Cor_DEPs_Pathway\\SD_82-VS-BS_82_Cor_DEPs.htm#gene9#gene9) | 5 (4.46%) | ko04626 |
| 10 | [Endocytosis](file:///E:\\%E7%8E%8B%E9%9B%AA%E5%B3%B0\\iTRAQ\\%E5%8D%8E%E5%A4%A7%E5%9F%BA%E5%9B%A0\\%E5%8D%8E%E5%A4%A7%E6%95%B0%E6%8D%AE\\%E8%9B%8B%E7%99%BD%E8%B4%A8%E7%BB%84%E4%B8%8E%E8%BD%AC%E5%BD%95%E7%BB%84%E5%85%B3%E8%81%94%E5%88%86%E6%9E%90\\F15FTSNCKF3616\\F15FTSNCKF3616\\Correlation\\SubCorrelation2Function\\Pathway\\SD_82-VS-BS_82_Cor_DEPs_Pathway\\SD_82-VS-BS_82_Cor_DEPs.htm#gene10#gene10) | 4 (3.57%) | ko04144 |
| 11 | [Flavonoid biosynthesis](file:///E:\\%E7%8E%8B%E9%9B%AA%E5%B3%B0\\iTRAQ\\%E5%8D%8E%E5%A4%A7%E5%9F%BA%E5%9B%A0\\%E5%8D%8E%E5%A4%A7%E6%95%B0%E6%8D%AE\\%E8%9B%8B%E7%99%BD%E8%B4%A8%E7%BB%84%E4%B8%8E%E8%BD%AC%E5%BD%95%E7%BB%84%E5%85%B3%E8%81%94%E5%88%86%E6%9E%90\\F15FTSNCKF3616\\F15FTSNCKF3616\\Correlation\\SubCorrelation2Function\\Pathway\\SD_82-VS-BS_82_Cor_DEPs_Pathway\\SD_82-VS-BS_82_Cor_DEPs.htm#gene11#gene11) | 4 (3.57%) | ko00941 |
| 12 | [Other glycan degradation](file:///E:\\%E7%8E%8B%E9%9B%AA%E5%B3%B0\\iTRAQ\\%E5%8D%8E%E5%A4%A7%E5%9F%BA%E5%9B%A0\\%E5%8D%8E%E5%A4%A7%E6%95%B0%E6%8D%AE\\%E8%9B%8B%E7%99%BD%E8%B4%A8%E7%BB%84%E4%B8%8E%E8%BD%AC%E5%BD%95%E7%BB%84%E5%85%B3%E8%81%94%E5%88%86%E6%9E%90\\F15FTSNCKF3616\\F15FTSNCKF3616\\Correlation\\SubCorrelation2Function\\Pathway\\SD_82-VS-BS_82_Cor_DEPs_Pathway\\SD_82-VS-BS_82_Cor_DEPs.htm#gene12#gene12) | 4 (3.57%) | ko00511 |
| 13 | [Ascorbate and aldarate metabolism](file:///E:\\%E7%8E%8B%E9%9B%AA%E5%B3%B0\\iTRAQ\\%E5%8D%8E%E5%A4%A7%E5%9F%BA%E5%9B%A0\\%E5%8D%8E%E5%A4%A7%E6%95%B0%E6%8D%AE\\%E8%9B%8B%E7%99%BD%E8%B4%A8%E7%BB%84%E4%B8%8E%E8%BD%AC%E5%BD%95%E7%BB%84%E5%85%B3%E8%81%94%E5%88%86%E6%9E%90\\F15FTSNCKF3616\\F15FTSNCKF3616\\Correlation\\SubCorrelation2Function\\Pathway\\SD_82-VS-BS_82_Cor_DEPs_Pathway\\SD_82-VS-BS_82_Cor_DEPs.htm#gene13#gene13) | 4 (3.57%) | ko00053 |
| 14 | [Isoflavonoid biosynthesis](file:///E:\\%E7%8E%8B%E9%9B%AA%E5%B3%B0\\iTRAQ\\%E5%8D%8E%E5%A4%A7%E5%9F%BA%E5%9B%A0\\%E5%8D%8E%E5%A4%A7%E6%95%B0%E6%8D%AE\\%E8%9B%8B%E7%99%BD%E8%B4%A8%E7%BB%84%E4%B8%8E%E8%BD%AC%E5%BD%95%E7%BB%84%E5%85%B3%E8%81%94%E5%88%86%E6%9E%90\\F15FTSNCKF3616\\F15FTSNCKF3616\\Correlation\\SubCorrelation2Function\\Pathway\\SD_82-VS-BS_82_Cor_DEPs_Pathway\\SD_82-VS-BS_82_Cor_DEPs.htm#gene14#gene14) | 3 (2.68%) | ko00943 |
| 15 | [Peroxisome](file:///E:\\%E7%8E%8B%E9%9B%AA%E5%B3%B0\\iTRAQ\\%E5%8D%8E%E5%A4%A7%E5%9F%BA%E5%9B%A0\\%E5%8D%8E%E5%A4%A7%E6%95%B0%E6%8D%AE\\%E8%9B%8B%E7%99%BD%E8%B4%A8%E7%BB%84%E4%B8%8E%E8%BD%AC%E5%BD%95%E7%BB%84%E5%85%B3%E8%81%94%E5%88%86%E6%9E%90\\F15FTSNCKF3616\\F15FTSNCKF3616\\Correlation\\SubCorrelation2Function\\Pathway\\SD_82-VS-BS_82_Cor_DEPs_Pathway\\SD_82-VS-BS_82_Cor_DEPs.htm#gene15#gene15) | 3 (2.68%) | ko04146 |
| 16 | [Biosynthesis of amino acids](file:///E:\\%E7%8E%8B%E9%9B%AA%E5%B3%B0\\iTRAQ\\%E5%8D%8E%E5%A4%A7%E5%9F%BA%E5%9B%A0\\%E5%8D%8E%E5%A4%A7%E6%95%B0%E6%8D%AE\\%E8%9B%8B%E7%99%BD%E8%B4%A8%E7%BB%84%E4%B8%8E%E8%BD%AC%E5%BD%95%E7%BB%84%E5%85%B3%E8%81%94%E5%88%86%E6%9E%90\\F15FTSNCKF3616\\F15FTSNCKF3616\\Correlation\\SubCorrelation2Function\\Pathway\\SD_82-VS-BS_82_Cor_DEPs_Pathway\\SD_82-VS-BS_82_Cor_DEPs.htm#gene16#gene16) | 3 (2.68%) | ko01230 |
| 17 | [Glycosaminoglycan degradation](file:///E:\\%E7%8E%8B%E9%9B%AA%E5%B3%B0\\iTRAQ\\%E5%8D%8E%E5%A4%A7%E5%9F%BA%E5%9B%A0\\%E5%8D%8E%E5%A4%A7%E6%95%B0%E6%8D%AE\\%E8%9B%8B%E7%99%BD%E8%B4%A8%E7%BB%84%E4%B8%8E%E8%BD%AC%E5%BD%95%E7%BB%84%E5%85%B3%E8%81%94%E5%88%86%E6%9E%90\\F15FTSNCKF3616\\F15FTSNCKF3616\\Correlation\\SubCorrelation2Function\\Pathway\\SD_82-VS-BS_82_Cor_DEPs_Pathway\\SD_82-VS-BS_82_Cor_DEPs.htm#gene17#gene17) | 3 (2.68%) | ko00531 |
| 18 | [Base excision repair](file:///E:\\%E7%8E%8B%E9%9B%AA%E5%B3%B0\\iTRAQ\\%E5%8D%8E%E5%A4%A7%E5%9F%BA%E5%9B%A0\\%E5%8D%8E%E5%A4%A7%E6%95%B0%E6%8D%AE\\%E8%9B%8B%E7%99%BD%E8%B4%A8%E7%BB%84%E4%B8%8E%E8%BD%AC%E5%BD%95%E7%BB%84%E5%85%B3%E8%81%94%E5%88%86%E6%9E%90\\F15FTSNCKF3616\\F15FTSNCKF3616\\Correlation\\SubCorrelation2Function\\Pathway\\SD_82-VS-BS_82_Cor_DEPs_Pathway\\SD_82-VS-BS_82_Cor_DEPs.htm#gene18#gene18) | 3 (2.68%) | ko03410 |
| 19 | [alpha-Linolenic acid metabolism](file:///E:\\%E7%8E%8B%E9%9B%AA%E5%B3%B0\\iTRAQ\\%E5%8D%8E%E5%A4%A7%E5%9F%BA%E5%9B%A0\\%E5%8D%8E%E5%A4%A7%E6%95%B0%E6%8D%AE\\%E8%9B%8B%E7%99%BD%E8%B4%A8%E7%BB%84%E4%B8%8E%E8%BD%AC%E5%BD%95%E7%BB%84%E5%85%B3%E8%81%94%E5%88%86%E6%9E%90\\F15FTSNCKF3616\\F15FTSNCKF3616\\Correlation\\SubCorrelation2Function\\Pathway\\SD_82-VS-BS_82_Cor_DEPs_Pathway\\SD_82-VS-BS_82_Cor_DEPs.htm#gene19#gene19) | 2 (1.79%) | ko00592 |
| 20 | [Protein processing in endoplasmic reticulum](file:///E:\\%E7%8E%8B%E9%9B%AA%E5%B3%B0\\iTRAQ\\%E5%8D%8E%E5%A4%A7%E5%9F%BA%E5%9B%A0\\%E5%8D%8E%E5%A4%A7%E6%95%B0%E6%8D%AE\\%E8%9B%8B%E7%99%BD%E8%B4%A8%E7%BB%84%E4%B8%8E%E8%BD%AC%E5%BD%95%E7%BB%84%E5%85%B3%E8%81%94%E5%88%86%E6%9E%90\\F15FTSNCKF3616\\F15FTSNCKF3616\\Correlation\\SubCorrelation2Function\\Pathway\\SD_82-VS-BS_82_Cor_DEPs_Pathway\\SD_82-VS-BS_82_Cor_DEPs.htm#gene20#gene20) | 2 (1.79%) | ko04141 |
| 21 | [Terpenoid backbone biosynthesis](file:///E:\\%E7%8E%8B%E9%9B%AA%E5%B3%B0\\iTRAQ\\%E5%8D%8E%E5%A4%A7%E5%9F%BA%E5%9B%A0\\%E5%8D%8E%E5%A4%A7%E6%95%B0%E6%8D%AE\\%E8%9B%8B%E7%99%BD%E8%B4%A8%E7%BB%84%E4%B8%8E%E8%BD%AC%E5%BD%95%E7%BB%84%E5%85%B3%E8%81%94%E5%88%86%E6%9E%90\\F15FTSNCKF3616\\F15FTSNCKF3616\\Correlation\\SubCorrelation2Function\\Pathway\\SD_82-VS-BS_82_Cor_DEPs_Pathway\\SD_82-VS-BS_82_Cor_DEPs.htm#gene21#gene21) | 2 (1.79%) | ko00900 |
| 22 | [Arachidonic acid metabolism](file:///E:\\%E7%8E%8B%E9%9B%AA%E5%B3%B0\\iTRAQ\\%E5%8D%8E%E5%A4%A7%E5%9F%BA%E5%9B%A0\\%E5%8D%8E%E5%A4%A7%E6%95%B0%E6%8D%AE\\%E8%9B%8B%E7%99%BD%E8%B4%A8%E7%BB%84%E4%B8%8E%E8%BD%AC%E5%BD%95%E7%BB%84%E5%85%B3%E8%81%94%E5%88%86%E6%9E%90\\F15FTSNCKF3616\\F15FTSNCKF3616\\Correlation\\SubCorrelation2Function\\Pathway\\SD_82-VS-BS_82_Cor_DEPs_Pathway\\SD_82-VS-BS_82_Cor_DEPs.htm#gene22#gene22) | 2 (1.79%) | ko00590 |
| 23 | [Spliceosome](file:///E:\\%E7%8E%8B%E9%9B%AA%E5%B3%B0\\iTRAQ\\%E5%8D%8E%E5%A4%A7%E5%9F%BA%E5%9B%A0\\%E5%8D%8E%E5%A4%A7%E6%95%B0%E6%8D%AE\\%E8%9B%8B%E7%99%BD%E8%B4%A8%E7%BB%84%E4%B8%8E%E8%BD%AC%E5%BD%95%E7%BB%84%E5%85%B3%E8%81%94%E5%88%86%E6%9E%90\\F15FTSNCKF3616\\F15FTSNCKF3616\\Correlation\\SubCorrelation2Function\\Pathway\\SD_82-VS-BS_82_Cor_DEPs_Pathway\\SD_82-VS-BS_82_Cor_DEPs.htm#gene23#gene23) | 2 (1.79%) | ko03040 |
| 24 | [Nitrogen metabolism](file:///E:\\%E7%8E%8B%E9%9B%AA%E5%B3%B0\\iTRAQ\\%E5%8D%8E%E5%A4%A7%E5%9F%BA%E5%9B%A0\\%E5%8D%8E%E5%A4%A7%E6%95%B0%E6%8D%AE\\%E8%9B%8B%E7%99%BD%E8%B4%A8%E7%BB%84%E4%B8%8E%E8%BD%AC%E5%BD%95%E7%BB%84%E5%85%B3%E8%81%94%E5%88%86%E6%9E%90\\F15FTSNCKF3616\\F15FTSNCKF3616\\Correlation\\SubCorrelation2Function\\Pathway\\SD_82-VS-BS_82_Cor_DEPs_Pathway\\SD_82-VS-BS_82_Cor_DEPs.htm#gene24#gene24) | 2 (1.79%) | ko00910 |
| 25 | [Aminoacyl-tRNA biosynthesis](file:///E:\\%E7%8E%8B%E9%9B%AA%E5%B3%B0\\iTRAQ\\%E5%8D%8E%E5%A4%A7%E5%9F%BA%E5%9B%A0\\%E5%8D%8E%E5%A4%A7%E6%95%B0%E6%8D%AE\\%E8%9B%8B%E7%99%BD%E8%B4%A8%E7%BB%84%E4%B8%8E%E8%BD%AC%E5%BD%95%E7%BB%84%E5%85%B3%E8%81%94%E5%88%86%E6%9E%90\\F15FTSNCKF3616\\F15FTSNCKF3616\\Correlation\\SubCorrelation2Function\\Pathway\\SD_82-VS-BS_82_Cor_DEPs_Pathway\\SD_82-VS-BS_82_Cor_DEPs.htm#gene25#gene25) | 2 (1.79%) | ko00970 |
| 26 | [Galactose metabolism](file:///E:\\%E7%8E%8B%E9%9B%AA%E5%B3%B0\\iTRAQ\\%E5%8D%8E%E5%A4%A7%E5%9F%BA%E5%9B%A0\\%E5%8D%8E%E5%A4%A7%E6%95%B0%E6%8D%AE\\%E8%9B%8B%E7%99%BD%E8%B4%A8%E7%BB%84%E4%B8%8E%E8%BD%AC%E5%BD%95%E7%BB%84%E5%85%B3%E8%81%94%E5%88%86%E6%9E%90\\F15FTSNCKF3616\\F15FTSNCKF3616\\Correlation\\SubCorrelation2Function\\Pathway\\SD_82-VS-BS_82_Cor_DEPs_Pathway\\SD_82-VS-BS_82_Cor_DEPs.htm#gene26#gene26) | 2 (1.79%) | ko00052 |
| 27 | [Phenylalanine metabolism](file:///E:\\%E7%8E%8B%E9%9B%AA%E5%B3%B0\\iTRAQ\\%E5%8D%8E%E5%A4%A7%E5%9F%BA%E5%9B%A0\\%E5%8D%8E%E5%A4%A7%E6%95%B0%E6%8D%AE\\%E8%9B%8B%E7%99%BD%E8%B4%A8%E7%BB%84%E4%B8%8E%E8%BD%AC%E5%BD%95%E7%BB%84%E5%85%B3%E8%81%94%E5%88%86%E6%9E%90\\F15FTSNCKF3616\\F15FTSNCKF3616\\Correlation\\SubCorrelation2Function\\Pathway\\SD_82-VS-BS_82_Cor_DEPs_Pathway\\SD_82-VS-BS_82_Cor_DEPs.htm#gene27#gene27) | 2 (1.79%) | ko00360 |
| 28 | [Ubiquinone and other terpenoid-quinone biosynthesis](file:///E:\\%E7%8E%8B%E9%9B%AA%E5%B3%B0\\iTRAQ\\%E5%8D%8E%E5%A4%A7%E5%9F%BA%E5%9B%A0\\%E5%8D%8E%E5%A4%A7%E6%95%B0%E6%8D%AE\\%E8%9B%8B%E7%99%BD%E8%B4%A8%E7%BB%84%E4%B8%8E%E8%BD%AC%E5%BD%95%E7%BB%84%E5%85%B3%E8%81%94%E5%88%86%E6%9E%90\\F15FTSNCKF3616\\F15FTSNCKF3616\\Correlation\\SubCorrelation2Function\\Pathway\\SD_82-VS-BS_82_Cor_DEPs_Pathway\\SD_82-VS-BS_82_Cor_DEPs.htm#gene28#gene28) | 2 (1.79%) | ko00130 |
| 29 | [Valine, leucine and isoleucine degradation](file:///E:\\%E7%8E%8B%E9%9B%AA%E5%B3%B0\\iTRAQ\\%E5%8D%8E%E5%A4%A7%E5%9F%BA%E5%9B%A0\\%E5%8D%8E%E5%A4%A7%E6%95%B0%E6%8D%AE\\%E8%9B%8B%E7%99%BD%E8%B4%A8%E7%BB%84%E4%B8%8E%E8%BD%AC%E5%BD%95%E7%BB%84%E5%85%B3%E8%81%94%E5%88%86%E6%9E%90\\F15FTSNCKF3616\\F15FTSNCKF3616\\Correlation\\SubCorrelation2Function\\Pathway\\SD_82-VS-BS_82_Cor_DEPs_Pathway\\SD_82-VS-BS_82_Cor_DEPs.htm#gene29#gene29) | 2 (1.79%) | ko00280 |
| 30 | [Fatty acid metabolism](file:///E:\\%E7%8E%8B%E9%9B%AA%E5%B3%B0\\iTRAQ\\%E5%8D%8E%E5%A4%A7%E5%9F%BA%E5%9B%A0\\%E5%8D%8E%E5%A4%A7%E6%95%B0%E6%8D%AE\\%E8%9B%8B%E7%99%BD%E8%B4%A8%E7%BB%84%E4%B8%8E%E8%BD%AC%E5%BD%95%E7%BB%84%E5%85%B3%E8%81%94%E5%88%86%E6%9E%90\\F15FTSNCKF3616\\F15FTSNCKF3616\\Correlation\\SubCorrelation2Function\\Pathway\\SD_82-VS-BS_82_Cor_DEPs_Pathway\\SD_82-VS-BS_82_Cor_DEPs.htm#gene30#gene30) | 2 (1.79%) | ko01212 |
| 31 | [Pentose and glucuronate interconversions](file:///E:\\%E7%8E%8B%E9%9B%AA%E5%B3%B0\\iTRAQ\\%E5%8D%8E%E5%A4%A7%E5%9F%BA%E5%9B%A0\\%E5%8D%8E%E5%A4%A7%E6%95%B0%E6%8D%AE\\%E8%9B%8B%E7%99%BD%E8%B4%A8%E7%BB%84%E4%B8%8E%E8%BD%AC%E5%BD%95%E7%BB%84%E5%85%B3%E8%81%94%E5%88%86%E6%9E%90\\F15FTSNCKF3616\\F15FTSNCKF3616\\Correlation\\SubCorrelation2Function\\Pathway\\SD_82-VS-BS_82_Cor_DEPs_Pathway\\SD_82-VS-BS_82_Cor_DEPs.htm#gene31#gene31) | 2 (1.79%) | ko00040 |
| 32 | [Fatty acid biosynthesis](file:///E:\\%E7%8E%8B%E9%9B%AA%E5%B3%B0\\iTRAQ\\%E5%8D%8E%E5%A4%A7%E5%9F%BA%E5%9B%A0\\%E5%8D%8E%E5%A4%A7%E6%95%B0%E6%8D%AE\\%E8%9B%8B%E7%99%BD%E8%B4%A8%E7%BB%84%E4%B8%8E%E8%BD%AC%E5%BD%95%E7%BB%84%E5%85%B3%E8%81%94%E5%88%86%E6%9E%90\\F15FTSNCKF3616\\F15FTSNCKF3616\\Correlation\\SubCorrelation2Function\\Pathway\\SD_82-VS-BS_82_Cor_DEPs_Pathway\\SD_82-VS-BS_82_Cor_DEPs.htm#gene32#gene32) | 1 (0.89%) | ko00061 |
| 33 | [Zeatin biosynthesis](file:///E:\\%E7%8E%8B%E9%9B%AA%E5%B3%B0\\iTRAQ\\%E5%8D%8E%E5%A4%A7%E5%9F%BA%E5%9B%A0\\%E5%8D%8E%E5%A4%A7%E6%95%B0%E6%8D%AE\\%E8%9B%8B%E7%99%BD%E8%B4%A8%E7%BB%84%E4%B8%8E%E8%BD%AC%E5%BD%95%E7%BB%84%E5%85%B3%E8%81%94%E5%88%86%E6%9E%90\\F15FTSNCKF3616\\F15FTSNCKF3616\\Correlation\\SubCorrelation2Function\\Pathway\\SD_82-VS-BS_82_Cor_DEPs_Pathway\\SD_82-VS-BS_82_Cor_DEPs.htm#gene33#gene33) | 1 (0.89%) | ko00908 |
| 34 | [Glycosphingolipid biosynthesis - ganglio series](file:///E:\\%E7%8E%8B%E9%9B%AA%E5%B3%B0\\iTRAQ\\%E5%8D%8E%E5%A4%A7%E5%9F%BA%E5%9B%A0\\%E5%8D%8E%E5%A4%A7%E6%95%B0%E6%8D%AE\\%E8%9B%8B%E7%99%BD%E8%B4%A8%E7%BB%84%E4%B8%8E%E8%BD%AC%E5%BD%95%E7%BB%84%E5%85%B3%E8%81%94%E5%88%86%E6%9E%90\\F15FTSNCKF3616\\F15FTSNCKF3616\\Correlation\\SubCorrelation2Function\\Pathway\\SD_82-VS-BS_82_Cor_DEPs_Pathway\\SD_82-VS-BS_82_Cor_DEPs.htm#gene34#gene34) | 1 (0.89%) | ko00604 |
| 35 | [ABC transporters](file:///E:\\%E7%8E%8B%E9%9B%AA%E5%B3%B0\\iTRAQ\\%E5%8D%8E%E5%A4%A7%E5%9F%BA%E5%9B%A0\\%E5%8D%8E%E5%A4%A7%E6%95%B0%E6%8D%AE\\%E8%9B%8B%E7%99%BD%E8%B4%A8%E7%BB%84%E4%B8%8E%E8%BD%AC%E5%BD%95%E7%BB%84%E5%85%B3%E8%81%94%E5%88%86%E6%9E%90\\F15FTSNCKF3616\\F15FTSNCKF3616\\Correlation\\SubCorrelation2Function\\Pathway\\SD_82-VS-BS_82_Cor_DEPs_Pathway\\SD_82-VS-BS_82_Cor_DEPs.htm#gene35#gene35) | 1 (0.89%) | ko02010 |
| 36 | [Linoleic acid metabolism](file:///E:\\%E7%8E%8B%E9%9B%AA%E5%B3%B0\\iTRAQ\\%E5%8D%8E%E5%A4%A7%E5%9F%BA%E5%9B%A0\\%E5%8D%8E%E5%A4%A7%E6%95%B0%E6%8D%AE\\%E8%9B%8B%E7%99%BD%E8%B4%A8%E7%BB%84%E4%B8%8E%E8%BD%AC%E5%BD%95%E7%BB%84%E5%85%B3%E8%81%94%E5%88%86%E6%9E%90\\F15FTSNCKF3616\\F15FTSNCKF3616\\Correlation\\SubCorrelation2Function\\Pathway\\SD_82-VS-BS_82_Cor_DEPs_Pathway\\SD_82-VS-BS_82_Cor_DEPs.htm#gene36#gene36) | 1 (0.89%) | ko00591 |
| 37 | [Porphyrin and chlorophyll metabolism](file:///E:\\%E7%8E%8B%E9%9B%AA%E5%B3%B0\\iTRAQ\\%E5%8D%8E%E5%A4%A7%E5%9F%BA%E5%9B%A0\\%E5%8D%8E%E5%A4%A7%E6%95%B0%E6%8D%AE\\%E8%9B%8B%E7%99%BD%E8%B4%A8%E7%BB%84%E4%B8%8E%E8%BD%AC%E5%BD%95%E7%BB%84%E5%85%B3%E8%81%94%E5%88%86%E6%9E%90\\F15FTSNCKF3616\\F15FTSNCKF3616\\Correlation\\SubCorrelation2Function\\Pathway\\SD_82-VS-BS_82_Cor_DEPs_Pathway\\SD_82-VS-BS_82_Cor_DEPs.htm#gene37#gene37) | 1 (0.89%) | ko00860 |
| 38 | [Oxidative phosphorylation](file:///E:\\%E7%8E%8B%E9%9B%AA%E5%B3%B0\\iTRAQ\\%E5%8D%8E%E5%A4%A7%E5%9F%BA%E5%9B%A0\\%E5%8D%8E%E5%A4%A7%E6%95%B0%E6%8D%AE\\%E8%9B%8B%E7%99%BD%E8%B4%A8%E7%BB%84%E4%B8%8E%E8%BD%AC%E5%BD%95%E7%BB%84%E5%85%B3%E8%81%94%E5%88%86%E6%9E%90\\F15FTSNCKF3616\\F15FTSNCKF3616\\Correlation\\SubCorrelation2Function\\Pathway\\SD_82-VS-BS_82_Cor_DEPs_Pathway\\SD_82-VS-BS_82_Cor_DEPs.htm#gene38#gene38) | 1 (0.89%) | ko00190 |
| 39 | [Anthocyanin biosynthesis](file:///E:\\%E7%8E%8B%E9%9B%AA%E5%B3%B0\\iTRAQ\\%E5%8D%8E%E5%A4%A7%E5%9F%BA%E5%9B%A0\\%E5%8D%8E%E5%A4%A7%E6%95%B0%E6%8D%AE\\%E8%9B%8B%E7%99%BD%E8%B4%A8%E7%BB%84%E4%B8%8E%E8%BD%AC%E5%BD%95%E7%BB%84%E5%85%B3%E8%81%94%E5%88%86%E6%9E%90\\F15FTSNCKF3616\\F15FTSNCKF3616\\Correlation\\SubCorrelation2Function\\Pathway\\SD_82-VS-BS_82_Cor_DEPs_Pathway\\SD_82-VS-BS_82_Cor_DEPs.htm#gene39#gene39) | 1 (0.89%) | ko00942 |
| 40 | [Inositol phosphate metabolism](file:///E:\\%E7%8E%8B%E9%9B%AA%E5%B3%B0\\iTRAQ\\%E5%8D%8E%E5%A4%A7%E5%9F%BA%E5%9B%A0\\%E5%8D%8E%E5%A4%A7%E6%95%B0%E6%8D%AE\\%E8%9B%8B%E7%99%BD%E8%B4%A8%E7%BB%84%E4%B8%8E%E8%BD%AC%E5%BD%95%E7%BB%84%E5%85%B3%E8%81%94%E5%88%86%E6%9E%90\\F15FTSNCKF3616\\F15FTSNCKF3616\\Correlation\\SubCorrelation2Function\\Pathway\\SD_82-VS-BS_82_Cor_DEPs_Pathway\\SD_82-VS-BS_82_Cor_DEPs.htm#gene40#gene40) | 1 (0.89%) | ko00562 |
| 41 | [Pantothenate and CoA biosynthesis](file:///E:\\%E7%8E%8B%E9%9B%AA%E5%B3%B0\\iTRAQ\\%E5%8D%8E%E5%A4%A7%E5%9F%BA%E5%9B%A0\\%E5%8D%8E%E5%A4%A7%E6%95%B0%E6%8D%AE\\%E8%9B%8B%E7%99%BD%E8%B4%A8%E7%BB%84%E4%B8%8E%E8%BD%AC%E5%BD%95%E7%BB%84%E5%85%B3%E8%81%94%E5%88%86%E6%9E%90\\F15FTSNCKF3616\\F15FTSNCKF3616\\Correlation\\SubCorrelation2Function\\Pathway\\SD_82-VS-BS_82_Cor_DEPs_Pathway\\SD_82-VS-BS_82_Cor_DEPs.htm#gene41#gene41) | 1 (0.89%) | ko00770 |
| 42 | [RNA transport](file:///E:\\%E7%8E%8B%E9%9B%AA%E5%B3%B0\\iTRAQ\\%E5%8D%8E%E5%A4%A7%E5%9F%BA%E5%9B%A0\\%E5%8D%8E%E5%A4%A7%E6%95%B0%E6%8D%AE\\%E8%9B%8B%E7%99%BD%E8%B4%A8%E7%BB%84%E4%B8%8E%E8%BD%AC%E5%BD%95%E7%BB%84%E5%85%B3%E8%81%94%E5%88%86%E6%9E%90\\F15FTSNCKF3616\\F15FTSNCKF3616\\Correlation\\SubCorrelation2Function\\Pathway\\SD_82-VS-BS_82_Cor_DEPs_Pathway\\SD_82-VS-BS_82_Cor_DEPs.htm#gene42#gene42) | 1 (0.89%) | ko03013 |
| 43 | [Carotenoid biosynthesis](file:///E:\\%E7%8E%8B%E9%9B%AA%E5%B3%B0\\iTRAQ\\%E5%8D%8E%E5%A4%A7%E5%9F%BA%E5%9B%A0\\%E5%8D%8E%E5%A4%A7%E6%95%B0%E6%8D%AE\\%E8%9B%8B%E7%99%BD%E8%B4%A8%E7%BB%84%E4%B8%8E%E8%BD%AC%E5%BD%95%E7%BB%84%E5%85%B3%E8%81%94%E5%88%86%E6%9E%90\\F15FTSNCKF3616\\F15FTSNCKF3616\\Correlation\\SubCorrelation2Function\\Pathway\\SD_82-VS-BS_82_Cor_DEPs_Pathway\\SD_82-VS-BS_82_Cor_DEPs.htm#gene43#gene43) | 1 (0.89%) | ko00906 |
| 44 | [2-Oxocarboxylic acid metabolism](file:///E:\\%E7%8E%8B%E9%9B%AA%E5%B3%B0\\iTRAQ\\%E5%8D%8E%E5%A4%A7%E5%9F%BA%E5%9B%A0\\%E5%8D%8E%E5%A4%A7%E6%95%B0%E6%8D%AE\\%E8%9B%8B%E7%99%BD%E8%B4%A8%E7%BB%84%E4%B8%8E%E8%BD%AC%E5%BD%95%E7%BB%84%E5%85%B3%E8%81%94%E5%88%86%E6%9E%90\\F15FTSNCKF3616\\F15FTSNCKF3616\\Correlation\\SubCorrelation2Function\\Pathway\\SD_82-VS-BS_82_Cor_DEPs_Pathway\\SD_82-VS-BS_82_Cor_DEPs.htm#gene44#gene44) | 1 (0.89%) | ko01210 |
| 45 | [Cysteine and methionine metabolism](file:///E:\\%E7%8E%8B%E9%9B%AA%E5%B3%B0\\iTRAQ\\%E5%8D%8E%E5%A4%A7%E5%9F%BA%E5%9B%A0\\%E5%8D%8E%E5%A4%A7%E6%95%B0%E6%8D%AE\\%E8%9B%8B%E7%99%BD%E8%B4%A8%E7%BB%84%E4%B8%8E%E8%BD%AC%E5%BD%95%E7%BB%84%E5%85%B3%E8%81%94%E5%88%86%E6%9E%90\\F15FTSNCKF3616\\F15FTSNCKF3616\\Correlation\\SubCorrelation2Function\\Pathway\\SD_82-VS-BS_82_Cor_DEPs_Pathway\\SD_82-VS-BS_82_Cor_DEPs.htm#gene45#gene45) | 1 (0.89%) | ko00270 |
| 46 | [Photosynthesis - antenna proteins](file:///E:\\%E7%8E%8B%E9%9B%AA%E5%B3%B0\\iTRAQ\\%E5%8D%8E%E5%A4%A7%E5%9F%BA%E5%9B%A0\\%E5%8D%8E%E5%A4%A7%E6%95%B0%E6%8D%AE\\%E8%9B%8B%E7%99%BD%E8%B4%A8%E7%BB%84%E4%B8%8E%E8%BD%AC%E5%BD%95%E7%BB%84%E5%85%B3%E8%81%94%E5%88%86%E6%9E%90\\F15FTSNCKF3616\\F15FTSNCKF3616\\Correlation\\SubCorrelation2Function\\Pathway\\SD_82-VS-BS_82_Cor_DEPs_Pathway\\SD_82-VS-BS_82_Cor_DEPs.htm#gene46#gene46) | 1 (0.89%) | ko00196 |
| 47 | [Fatty acid degradation](file:///E:\\%E7%8E%8B%E9%9B%AA%E5%B3%B0\\iTRAQ\\%E5%8D%8E%E5%A4%A7%E5%9F%BA%E5%9B%A0\\%E5%8D%8E%E5%A4%A7%E6%95%B0%E6%8D%AE\\%E8%9B%8B%E7%99%BD%E8%B4%A8%E7%BB%84%E4%B8%8E%E8%BD%AC%E5%BD%95%E7%BB%84%E5%85%B3%E8%81%94%E5%88%86%E6%9E%90\\F15FTSNCKF3616\\F15FTSNCKF3616\\Correlation\\SubCorrelation2Function\\Pathway\\SD_82-VS-BS_82_Cor_DEPs_Pathway\\SD_82-VS-BS_82_Cor_DEPs.htm#gene47#gene47) | 1 (0.89%) | ko00071 |
| 48 | [Biosynthesis of unsaturated fatty acids](file:///E:\\%E7%8E%8B%E9%9B%AA%E5%B3%B0\\iTRAQ\\%E5%8D%8E%E5%A4%A7%E5%9F%BA%E5%9B%A0\\%E5%8D%8E%E5%A4%A7%E6%95%B0%E6%8D%AE\\%E8%9B%8B%E7%99%BD%E8%B4%A8%E7%BB%84%E4%B8%8E%E8%BD%AC%E5%BD%95%E7%BB%84%E5%85%B3%E8%81%94%E5%88%86%E6%9E%90\\F15FTSNCKF3616\\F15FTSNCKF3616\\Correlation\\SubCorrelation2Function\\Pathway\\SD_82-VS-BS_82_Cor_DEPs_Pathway\\SD_82-VS-BS_82_Cor_DEPs.htm#gene48#gene48) | 1 (0.89%) | ko01040 |
| 49 | [Folate biosynthesis](file:///E:\\%E7%8E%8B%E9%9B%AA%E5%B3%B0\\iTRAQ\\%E5%8D%8E%E5%A4%A7%E5%9F%BA%E5%9B%A0\\%E5%8D%8E%E5%A4%A7%E6%95%B0%E6%8D%AE\\%E8%9B%8B%E7%99%BD%E8%B4%A8%E7%BB%84%E4%B8%8E%E8%BD%AC%E5%BD%95%E7%BB%84%E5%85%B3%E8%81%94%E5%88%86%E6%9E%90\\F15FTSNCKF3616\\F15FTSNCKF3616\\Correlation\\SubCorrelation2Function\\Pathway\\SD_82-VS-BS_82_Cor_DEPs_Pathway\\SD_82-VS-BS_82_Cor_DEPs.htm#gene49#gene49) | 1 (0.89%) | ko00790 |
| 50 | [Monoterpenoid biosynthesis](file:///E:\\%E7%8E%8B%E9%9B%AA%E5%B3%B0\\iTRAQ\\%E5%8D%8E%E5%A4%A7%E5%9F%BA%E5%9B%A0\\%E5%8D%8E%E5%A4%A7%E6%95%B0%E6%8D%AE\\%E8%9B%8B%E7%99%BD%E8%B4%A8%E7%BB%84%E4%B8%8E%E8%BD%AC%E5%BD%95%E7%BB%84%E5%85%B3%E8%81%94%E5%88%86%E6%9E%90\\F15FTSNCKF3616\\F15FTSNCKF3616\\Correlation\\SubCorrelation2Function\\Pathway\\SD_82-VS-BS_82_Cor_DEPs_Pathway\\SD_82-VS-BS_82_Cor_DEPs.htm#gene50#gene50) | 1 (0.89%) | ko00902 |
| 51 | [Glucosinolate biosynthesis](file:///E:\\%E7%8E%8B%E9%9B%AA%E5%B3%B0\\iTRAQ\\%E5%8D%8E%E5%A4%A7%E5%9F%BA%E5%9B%A0\\%E5%8D%8E%E5%A4%A7%E6%95%B0%E6%8D%AE\\%E8%9B%8B%E7%99%BD%E8%B4%A8%E7%BB%84%E4%B8%8E%E8%BD%AC%E5%BD%95%E7%BB%84%E5%85%B3%E8%81%94%E5%88%86%E6%9E%90\\F15FTSNCKF3616\\F15FTSNCKF3616\\Correlation\\SubCorrelation2Function\\Pathway\\SD_82-VS-BS_82_Cor_DEPs_Pathway\\SD_82-VS-BS_82_Cor_DEPs.htm#gene51#gene51) | 1 (0.89%) | ko00966 |
| 52 | [Phenylalanine, tyrosine and tryptophan biosynthesis](file:///E:\\%E7%8E%8B%E9%9B%AA%E5%B3%B0\\iTRAQ\\%E5%8D%8E%E5%A4%A7%E5%9F%BA%E5%9B%A0\\%E5%8D%8E%E5%A4%A7%E6%95%B0%E6%8D%AE\\%E8%9B%8B%E7%99%BD%E8%B4%A8%E7%BB%84%E4%B8%8E%E8%BD%AC%E5%BD%95%E7%BB%84%E5%85%B3%E8%81%94%E5%88%86%E6%9E%90\\F15FTSNCKF3616\\F15FTSNCKF3616\\Correlation\\SubCorrelation2Function\\Pathway\\SD_82-VS-BS_82_Cor_DEPs_Pathway\\SD_82-VS-BS_82_Cor_DEPs.htm#gene52#gene52) | 1 (0.89%) | ko00400 |
| 53 | [Butanoate metabolism](file:///E:\\%E7%8E%8B%E9%9B%AA%E5%B3%B0\\iTRAQ\\%E5%8D%8E%E5%A4%A7%E5%9F%BA%E5%9B%A0\\%E5%8D%8E%E5%A4%A7%E6%95%B0%E6%8D%AE\\%E8%9B%8B%E7%99%BD%E8%B4%A8%E7%BB%84%E4%B8%8E%E8%BD%AC%E5%BD%95%E7%BB%84%E5%85%B3%E8%81%94%E5%88%86%E6%9E%90\\F15FTSNCKF3616\\F15FTSNCKF3616\\Correlation\\SubCorrelation2Function\\Pathway\\SD_82-VS-BS_82_Cor_DEPs_Pathway\\SD_82-VS-BS_82_Cor_DEPs.htm#gene53#gene53) | 1 (0.89%) | ko00650 |
| 54 | [mRNA surveillance pathway](file:///E:\\%E7%8E%8B%E9%9B%AA%E5%B3%B0\\iTRAQ\\%E5%8D%8E%E5%A4%A7%E5%9F%BA%E5%9B%A0\\%E5%8D%8E%E5%A4%A7%E6%95%B0%E6%8D%AE\\%E8%9B%8B%E7%99%BD%E8%B4%A8%E7%BB%84%E4%B8%8E%E8%BD%AC%E5%BD%95%E7%BB%84%E5%85%B3%E8%81%94%E5%88%86%E6%9E%90\\F15FTSNCKF3616\\F15FTSNCKF3616\\Correlation\\SubCorrelation2Function\\Pathway\\SD_82-VS-BS_82_Cor_DEPs_Pathway\\SD_82-VS-BS_82_Cor_DEPs.htm#gene54#gene54) | 1 (0.89%) | ko03015 |
| 55 | [Flavone and flavonol biosynthesis](file:///E:\\%E7%8E%8B%E9%9B%AA%E5%B3%B0\\iTRAQ\\%E5%8D%8E%E5%A4%A7%E5%9F%BA%E5%9B%A0\\%E5%8D%8E%E5%A4%A7%E6%95%B0%E6%8D%AE\\%E8%9B%8B%E7%99%BD%E8%B4%A8%E7%BB%84%E4%B8%8E%E8%BD%AC%E5%BD%95%E7%BB%84%E5%85%B3%E8%81%94%E5%88%86%E6%9E%90\\F15FTSNCKF3616\\F15FTSNCKF3616\\Correlation\\SubCorrelation2Function\\Pathway\\SD_82-VS-BS_82_Cor_DEPs_Pathway\\SD_82-VS-BS_82_Cor_DEPs.htm#gene55#gene55) | 1 (0.89%) | ko00944 |
| 56 | [Sphingolipid metabolism](file:///E:\\%E7%8E%8B%E9%9B%AA%E5%B3%B0\\iTRAQ\\%E5%8D%8E%E5%A4%A7%E5%9F%BA%E5%9B%A0\\%E5%8D%8E%E5%A4%A7%E6%95%B0%E6%8D%AE\\%E8%9B%8B%E7%99%BD%E8%B4%A8%E7%BB%84%E4%B8%8E%E8%BD%AC%E5%BD%95%E7%BB%84%E5%85%B3%E8%81%94%E5%88%86%E6%9E%90\\F15FTSNCKF3616\\F15FTSNCKF3616\\Correlation\\SubCorrelation2Function\\Pathway\\SD_82-VS-BS_82_Cor_DEPs_Pathway\\SD_82-VS-BS_82_Cor_DEPs.htm#gene56#gene56) | 1 (0.89%) | ko00600 |
| 57 | [Isoquinoline alkaloid biosynthesis](file:///E:\\%E7%8E%8B%E9%9B%AA%E5%B3%B0\\iTRAQ\\%E5%8D%8E%E5%A4%A7%E5%9F%BA%E5%9B%A0\\%E5%8D%8E%E5%A4%A7%E6%95%B0%E6%8D%AE\\%E8%9B%8B%E7%99%BD%E8%B4%A8%E7%BB%84%E4%B8%8E%E8%BD%AC%E5%BD%95%E7%BB%84%E5%85%B3%E8%81%94%E5%88%86%E6%9E%90\\F15FTSNCKF3616\\F15FTSNCKF3616\\Correlation\\SubCorrelation2Function\\Pathway\\SD_82-VS-BS_82_Cor_DEPs_Pathway\\SD_82-VS-BS_82_Cor_DEPs.htm#gene57#gene57) | 1 (0.89%) | ko00950 |
| 58 | [Stilbenoid, diarylheptanoid and gingerol biosynthesis](file:///E:\\%E7%8E%8B%E9%9B%AA%E5%B3%B0\\iTRAQ\\%E5%8D%8E%E5%A4%A7%E5%9F%BA%E5%9B%A0\\%E5%8D%8E%E5%A4%A7%E6%95%B0%E6%8D%AE\\%E8%9B%8B%E7%99%BD%E8%B4%A8%E7%BB%84%E4%B8%8E%E8%BD%AC%E5%BD%95%E7%BB%84%E5%85%B3%E8%81%94%E5%88%86%E6%9E%90\\F15FTSNCKF3616\\F15FTSNCKF3616\\Correlation\\SubCorrelation2Function\\Pathway\\SD_82-VS-BS_82_Cor_DEPs_Pathway\\SD_82-VS-BS_82_Cor_DEPs.htm#gene58#gene58) | 1 (0.89%) | ko00945 |
| 59 | [Synthesis and degradation of ketone bodies](file:///E:\\%E7%8E%8B%E9%9B%AA%E5%B3%B0\\iTRAQ\\%E5%8D%8E%E5%A4%A7%E5%9F%BA%E5%9B%A0\\%E5%8D%8E%E5%A4%A7%E6%95%B0%E6%8D%AE\\%E8%9B%8B%E7%99%BD%E8%B4%A8%E7%BB%84%E4%B8%8E%E8%BD%AC%E5%BD%95%E7%BB%84%E5%85%B3%E8%81%94%E5%88%86%E6%9E%90\\F15FTSNCKF3616\\F15FTSNCKF3616\\Correlation\\SubCorrelation2Function\\Pathway\\SD_82-VS-BS_82_Cor_DEPs_Pathway\\SD_82-VS-BS_82_Cor_DEPs.htm#gene59#gene59) | 1 (0.89%) | ko00072 |
| 60 | [Pyrimidine metabolism](file:///E:\\%E7%8E%8B%E9%9B%AA%E5%B3%B0\\iTRAQ\\%E5%8D%8E%E5%A4%A7%E5%9F%BA%E5%9B%A0\\%E5%8D%8E%E5%A4%A7%E6%95%B0%E6%8D%AE\\%E8%9B%8B%E7%99%BD%E8%B4%A8%E7%BB%84%E4%B8%8E%E8%BD%AC%E5%BD%95%E7%BB%84%E5%85%B3%E8%81%94%E5%88%86%E6%9E%90\\F15FTSNCKF3616\\F15FTSNCKF3616\\Correlation\\SubCorrelation2Function\\Pathway\\SD_82-VS-BS_82_Cor_DEPs_Pathway\\SD_82-VS-BS_82_Cor_DEPs.htm#gene60#gene60) | 1 (0.89%) | ko00240 |
| 61 | [Lysine biosynthesis](file:///E:\\%E7%8E%8B%E9%9B%AA%E5%B3%B0\\iTRAQ\\%E5%8D%8E%E5%A4%A7%E5%9F%BA%E5%9B%A0\\%E5%8D%8E%E5%A4%A7%E6%95%B0%E6%8D%AE\\%E8%9B%8B%E7%99%BD%E8%B4%A8%E7%BB%84%E4%B8%8E%E8%BD%AC%E5%BD%95%E7%BB%84%E5%85%B3%E8%81%94%E5%88%86%E6%9E%90\\F15FTSNCKF3616\\F15FTSNCKF3616\\Correlation\\SubCorrelation2Function\\Pathway\\SD_82-VS-BS_82_Cor_DEPs_Pathway\\SD_82-VS-BS_82_Cor_DEPs.htm#gene61#gene61) | 1 (0.89%) | ko00300 |
| 62 | [Cutin, suberine and wax biosynthesis](file:///E:\\%E7%8E%8B%E9%9B%AA%E5%B3%B0\\iTRAQ\\%E5%8D%8E%E5%A4%A7%E5%9F%BA%E5%9B%A0\\%E5%8D%8E%E5%A4%A7%E6%95%B0%E6%8D%AE\\%E8%9B%8B%E7%99%BD%E8%B4%A8%E7%BB%84%E4%B8%8E%E8%BD%AC%E5%BD%95%E7%BB%84%E5%85%B3%E8%81%94%E5%88%86%E6%9E%90\\F15FTSNCKF3616\\F15FTSNCKF3616\\Correlation\\SubCorrelation2Function\\Pathway\\SD_82-VS-BS_82_Cor_DEPs_Pathway\\SD_82-VS-BS_82_Cor_DEPs.htm#gene62#gene62) | 1 (0.89%) | ko00073 |
| 63 | [Glycerophospholipid metabolism](file:///E:\\%E7%8E%8B%E9%9B%AA%E5%B3%B0\\iTRAQ\\%E5%8D%8E%E5%A4%A7%E5%9F%BA%E5%9B%A0\\%E5%8D%8E%E5%A4%A7%E6%95%B0%E6%8D%AE\\%E8%9B%8B%E7%99%BD%E8%B4%A8%E7%BB%84%E4%B8%8E%E8%BD%AC%E5%BD%95%E7%BB%84%E5%85%B3%E8%81%94%E5%88%86%E6%9E%90\\F15FTSNCKF3616\\F15FTSNCKF3616\\Correlation\\SubCorrelation2Function\\Pathway\\SD_82-VS-BS_82_Cor_DEPs_Pathway\\SD_82-VS-BS_82_Cor_DEPs.htm#gene63#gene63) | 1 (0.89%) | ko00564 |
| 64 | [Biotin metabolism](file:///E:\\%E7%8E%8B%E9%9B%AA%E5%B3%B0\\iTRAQ\\%E5%8D%8E%E5%A4%A7%E5%9F%BA%E5%9B%A0\\%E5%8D%8E%E5%A4%A7%E6%95%B0%E6%8D%AE\\%E8%9B%8B%E7%99%BD%E8%B4%A8%E7%BB%84%E4%B8%8E%E8%BD%AC%E5%BD%95%E7%BB%84%E5%85%B3%E8%81%94%E5%88%86%E6%9E%90\\F15FTSNCKF3616\\F15FTSNCKF3616\\Correlation\\SubCorrelation2Function\\Pathway\\SD_82-VS-BS_82_Cor_DEPs_Pathway\\SD_82-VS-BS_82_Cor_DEPs.htm#gene64#gene64) | 1 (0.89%) | ko00780 |
| 65 | [Cyanoamino acid metabolism](file:///E:\\%E7%8E%8B%E9%9B%AA%E5%B3%B0\\iTRAQ\\%E5%8D%8E%E5%A4%A7%E5%9F%BA%E5%9B%A0\\%E5%8D%8E%E5%A4%A7%E6%95%B0%E6%8D%AE\\%E8%9B%8B%E7%99%BD%E8%B4%A8%E7%BB%84%E4%B8%8E%E8%BD%AC%E5%BD%95%E7%BB%84%E5%85%B3%E8%81%94%E5%88%86%E6%9E%90\\F15FTSNCKF3616\\F15FTSNCKF3616\\Correlation\\SubCorrelation2Function\\Pathway\\SD_82-VS-BS_82_Cor_DEPs_Pathway\\SD_82-VS-BS_82_Cor_DEPs.htm#gene65#gene65) | 1 (0.89%) | ko00460 |
| 66 | [Insulin resistance](file:///E:\\%E7%8E%8B%E9%9B%AA%E5%B3%B0\\iTRAQ\\%E5%8D%8E%E5%A4%A7%E5%9F%BA%E5%9B%A0\\%E5%8D%8E%E5%A4%A7%E6%95%B0%E6%8D%AE\\%E8%9B%8B%E7%99%BD%E8%B4%A8%E7%BB%84%E4%B8%8E%E8%BD%AC%E5%BD%95%E7%BB%84%E5%85%B3%E8%81%94%E5%88%86%E6%9E%90\\F15FTSNCKF3616\\F15FTSNCKF3616\\Correlation\\SubCorrelation2Function\\Pathway\\SD_82-VS-BS_82_Cor_DEPs_Pathway\\SD_82-VS-BS_82_Cor_DEPs.htm#gene66#gene66) | 1 (0.89%) | ko04931 |
| 67 | [Valine, leucine and isoleucine biosynthesis](file:///E:\\%E7%8E%8B%E9%9B%AA%E5%B3%B0\\iTRAQ\\%E5%8D%8E%E5%A4%A7%E5%9F%BA%E5%9B%A0\\%E5%8D%8E%E5%A4%A7%E6%95%B0%E6%8D%AE\\%E8%9B%8B%E7%99%BD%E8%B4%A8%E7%BB%84%E4%B8%8E%E8%BD%AC%E5%BD%95%E7%BB%84%E5%85%B3%E8%81%94%E5%88%86%E6%9E%90\\F15FTSNCKF3616\\F15FTSNCKF3616\\Correlation\\SubCorrelation2Function\\Pathway\\SD_82-VS-BS_82_Cor_DEPs_Pathway\\SD_82-VS-BS_82_Cor_DEPs.htm#gene67#gene67) | 1 (0.89%) | ko00290 |
| 68 | [Phagosome](file:///E:\\%E7%8E%8B%E9%9B%AA%E5%B3%B0\\iTRAQ\\%E5%8D%8E%E5%A4%A7%E5%9F%BA%E5%9B%A0\\%E5%8D%8E%E5%A4%A7%E6%95%B0%E6%8D%AE\\%E8%9B%8B%E7%99%BD%E8%B4%A8%E7%BB%84%E4%B8%8E%E8%BD%AC%E5%BD%95%E7%BB%84%E5%85%B3%E8%81%94%E5%88%86%E6%9E%90\\F15FTSNCKF3616\\F15FTSNCKF3616\\Correlation\\SubCorrelation2Function\\Pathway\\SD_82-VS-BS_82_Cor_DEPs_Pathway\\SD_82-VS-BS_82_Cor_DEPs.htm#gene68#gene68) | 1 (0.89%) | ko04145 |
| 69 | [Regulation of autophagy](file:///E:\\%E7%8E%8B%E9%9B%AA%E5%B3%B0\\iTRAQ\\%E5%8D%8E%E5%A4%A7%E5%9F%BA%E5%9B%A0\\%E5%8D%8E%E5%A4%A7%E6%95%B0%E6%8D%AE\\%E8%9B%8B%E7%99%BD%E8%B4%A8%E7%BB%84%E4%B8%8E%E8%BD%AC%E5%BD%95%E7%BB%84%E5%85%B3%E8%81%94%E5%88%86%E6%9E%90\\F15FTSNCKF3616\\F15FTSNCKF3616\\Correlation\\SubCorrelation2Function\\Pathway\\SD_82-VS-BS_82_Cor_DEPs_Pathway\\SD_82-VS-BS_82_Cor_DEPs.htm#gene69#gene69) | 1 (0.89%) | ko04140 |
| 70 | [Tyrosine metabolism](file:///E:\\%E7%8E%8B%E9%9B%AA%E5%B3%B0\\iTRAQ\\%E5%8D%8E%E5%A4%A7%E5%9F%BA%E5%9B%A0\\%E5%8D%8E%E5%A4%A7%E6%95%B0%E6%8D%AE\\%E8%9B%8B%E7%99%BD%E8%B4%A8%E7%BB%84%E4%B8%8E%E8%BD%AC%E5%BD%95%E7%BB%84%E5%85%B3%E8%81%94%E5%88%86%E6%9E%90\\F15FTSNCKF3616\\F15FTSNCKF3616\\Correlation\\SubCorrelation2Function\\Pathway\\SD_82-VS-BS_82_Cor_DEPs_Pathway\\SD_82-VS-BS_82_Cor_DEPs.htm#gene70#gene70) | 1 (0.89%) | ko00350 |
| **SD82-VS-SD59_Cor_DEGs_DEPs** | | | |
| # | Pathway | Diff Proteins with pathway annotation (170) | Pathway ID |
| 1 | [Metabolic pathways](file:///E:\\%E7%8E%8B%E9%9B%AA%E5%B3%B0\\iTRAQ\\%E5%8D%8E%E5%A4%A7%E5%9F%BA%E5%9B%A0\\%E5%8D%8E%E5%A4%A7%E6%95%B0%E6%8D%AE\\%E8%9B%8B%E7%99%BD%E8%B4%A8%E7%BB%84%E4%B8%8E%E8%BD%AC%E5%BD%95%E7%BB%84%E5%85%B3%E8%81%94%E5%88%86%E6%9E%90\\F15FTSNCKF3616\\F15FTSNCKF3616\\Correlation\\SubCorrelation2Function\\Pathway\\SD_82-VS-SD_59_Cor_DEPs_Pathway\\SD_82-VS-SD_59_Cor_DEPs.htm#gene1#gene1) | 65 (38.24%) | ko01100 |
| 2 | [Biosynthesis of secondary metabolites](file:///E:\\%E7%8E%8B%E9%9B%AA%E5%B3%B0\\iTRAQ\\%E5%8D%8E%E5%A4%A7%E5%9F%BA%E5%9B%A0\\%E5%8D%8E%E5%A4%A7%E6%95%B0%E6%8D%AE\\%E8%9B%8B%E7%99%BD%E8%B4%A8%E7%BB%84%E4%B8%8E%E8%BD%AC%E5%BD%95%E7%BB%84%E5%85%B3%E8%81%94%E5%88%86%E6%9E%90\\F15FTSNCKF3616\\F15FTSNCKF3616\\Correlation\\SubCorrelation2Function\\Pathway\\SD_82-VS-SD_59_Cor_DEPs_Pathway\\SD_82-VS-SD_59_Cor_DEPs.htm#gene2#gene2) | 34 (20%) | ko01110 |
| 3 | [Starch and sucrose metabolism](file:///E:\\%E7%8E%8B%E9%9B%AA%E5%B3%B0\\iTRAQ\\%E5%8D%8E%E5%A4%A7%E5%9F%BA%E5%9B%A0\\%E5%8D%8E%E5%A4%A7%E6%95%B0%E6%8D%AE\\%E8%9B%8B%E7%99%BD%E8%B4%A8%E7%BB%84%E4%B8%8E%E8%BD%AC%E5%BD%95%E7%BB%84%E5%85%B3%E8%81%94%E5%88%86%E6%9E%90\\F15FTSNCKF3616\\F15FTSNCKF3616\\Correlation\\SubCorrelation2Function\\Pathway\\SD_82-VS-SD_59_Cor_DEPs_Pathway\\SD_82-VS-SD_59_Cor_DEPs.htm#gene3#gene3) | 15 (8.82%) | ko00500 |
| 4 | [Amino sugar and nucleotide sugar metabolism](file:///E:\\%E7%8E%8B%E9%9B%AA%E5%B3%B0\\iTRAQ\\%E5%8D%8E%E5%A4%A7%E5%9F%BA%E5%9B%A0\\%E5%8D%8E%E5%A4%A7%E6%95%B0%E6%8D%AE\\%E8%9B%8B%E7%99%BD%E8%B4%A8%E7%BB%84%E4%B8%8E%E8%BD%AC%E5%BD%95%E7%BB%84%E5%85%B3%E8%81%94%E5%88%86%E6%9E%90\\F15FTSNCKF3616\\F15FTSNCKF3616\\Correlation\\SubCorrelation2Function\\Pathway\\SD_82-VS-SD_59_Cor_DEPs_Pathway\\SD_82-VS-SD_59_Cor_DEPs.htm#gene4#gene4) | 12 (7.06%) | ko00520 |
| 5 | [Plant-pathogen interaction](file:///E:\\%E7%8E%8B%E9%9B%AA%E5%B3%B0\\iTRAQ\\%E5%8D%8E%E5%A4%A7%E5%9F%BA%E5%9B%A0\\%E5%8D%8E%E5%A4%A7%E6%95%B0%E6%8D%AE\\%E8%9B%8B%E7%99%BD%E8%B4%A8%E7%BB%84%E4%B8%8E%E8%BD%AC%E5%BD%95%E7%BB%84%E5%85%B3%E8%81%94%E5%88%86%E6%9E%90\\F15FTSNCKF3616\\F15FTSNCKF3616\\Correlation\\SubCorrelation2Function\\Pathway\\SD_82-VS-SD_59_Cor_DEPs_Pathway\\SD_82-VS-SD_59_Cor_DEPs.htm#gene5#gene5) | 10 (5.88%) | ko04626 |
| 6 | [Phenylpropanoid biosynthesis](file:///E:\\%E7%8E%8B%E9%9B%AA%E5%B3%B0\\iTRAQ\\%E5%8D%8E%E5%A4%A7%E5%9F%BA%E5%9B%A0\\%E5%8D%8E%E5%A4%A7%E6%95%B0%E6%8D%AE\\%E8%9B%8B%E7%99%BD%E8%B4%A8%E7%BB%84%E4%B8%8E%E8%BD%AC%E5%BD%95%E7%BB%84%E5%85%B3%E8%81%94%E5%88%86%E6%9E%90\\F15FTSNCKF3616\\F15FTSNCKF3616\\Correlation\\SubCorrelation2Function\\Pathway\\SD_82-VS-SD_59_Cor_DEPs_Pathway\\SD_82-VS-SD_59_Cor_DEPs.htm#gene6#gene6) | 9 (5.29%) | ko00940 |
| 7 | [Endocytosis](file:///E:\\%E7%8E%8B%E9%9B%AA%E5%B3%B0\\iTRAQ\\%E5%8D%8E%E5%A4%A7%E5%9F%BA%E5%9B%A0\\%E5%8D%8E%E5%A4%A7%E6%95%B0%E6%8D%AE\\%E8%9B%8B%E7%99%BD%E8%B4%A8%E7%BB%84%E4%B8%8E%E8%BD%AC%E5%BD%95%E7%BB%84%E5%85%B3%E8%81%94%E5%88%86%E6%9E%90\\F15FTSNCKF3616\\F15FTSNCKF3616\\Correlation\\SubCorrelation2Function\\Pathway\\SD_82-VS-SD_59_Cor_DEPs_Pathway\\SD_82-VS-SD_59_Cor_DEPs.htm#gene7#gene7) | 7 (4.12%) | ko04144 |
| 8 | [Carbon metabolism](file:///E:\\%E7%8E%8B%E9%9B%AA%E5%B3%B0\\iTRAQ\\%E5%8D%8E%E5%A4%A7%E5%9F%BA%E5%9B%A0\\%E5%8D%8E%E5%A4%A7%E6%95%B0%E6%8D%AE\\%E8%9B%8B%E7%99%BD%E8%B4%A8%E7%BB%84%E4%B8%8E%E8%BD%AC%E5%BD%95%E7%BB%84%E5%85%B3%E8%81%94%E5%88%86%E6%9E%90\\F15FTSNCKF3616\\F15FTSNCKF3616\\Correlation\\SubCorrelation2Function\\Pathway\\SD_82-VS-SD_59_Cor_DEPs_Pathway\\SD_82-VS-SD_59_Cor_DEPs.htm#gene8#gene8) | 7 (4.12%) | ko01200 |
| 9 | [Biosynthesis of amino acids](file:///E:\\%E7%8E%8B%E9%9B%AA%E5%B3%B0\\iTRAQ\\%E5%8D%8E%E5%A4%A7%E5%9F%BA%E5%9B%A0\\%E5%8D%8E%E5%A4%A7%E6%95%B0%E6%8D%AE\\%E8%9B%8B%E7%99%BD%E8%B4%A8%E7%BB%84%E4%B8%8E%E8%BD%AC%E5%BD%95%E7%BB%84%E5%85%B3%E8%81%94%E5%88%86%E6%9E%90\\F15FTSNCKF3616\\F15FTSNCKF3616\\Correlation\\SubCorrelation2Function\\Pathway\\SD_82-VS-SD_59_Cor_DEPs_Pathway\\SD_82-VS-SD_59_Cor_DEPs.htm#gene9#gene9) | 6 (3.53%) | ko01230 |
| 10 | [Glutathione metabolism](file:///E:\\%E7%8E%8B%E9%9B%AA%E5%B3%B0\\iTRAQ\\%E5%8D%8E%E5%A4%A7%E5%9F%BA%E5%9B%A0\\%E5%8D%8E%E5%A4%A7%E6%95%B0%E6%8D%AE\\%E8%9B%8B%E7%99%BD%E8%B4%A8%E7%BB%84%E4%B8%8E%E8%BD%AC%E5%BD%95%E7%BB%84%E5%85%B3%E8%81%94%E5%88%86%E6%9E%90\\F15FTSNCKF3616\\F15FTSNCKF3616\\Correlation\\SubCorrelation2Function\\Pathway\\SD_82-VS-SD_59_Cor_DEPs_Pathway\\SD_82-VS-SD_59_Cor_DEPs.htm#gene10#gene10) | 6 (3.53%) | ko00480 |
| 11 | [Peroxisome](file:///E:\\%E7%8E%8B%E9%9B%AA%E5%B3%B0\\iTRAQ\\%E5%8D%8E%E5%A4%A7%E5%9F%BA%E5%9B%A0\\%E5%8D%8E%E5%A4%A7%E6%95%B0%E6%8D%AE\\%E8%9B%8B%E7%99%BD%E8%B4%A8%E7%BB%84%E4%B8%8E%E8%BD%AC%E5%BD%95%E7%BB%84%E5%85%B3%E8%81%94%E5%88%86%E6%9E%90\\F15FTSNCKF3616\\F15FTSNCKF3616\\Correlation\\SubCorrelation2Function\\Pathway\\SD_82-VS-SD_59_Cor_DEPs_Pathway\\SD_82-VS-SD_59_Cor_DEPs.htm#gene11#gene11) | 5 (2.94%) | ko04146 |
| 12 | [Glyoxylate and dicarboxylate metabolism](file:///E:\\%E7%8E%8B%E9%9B%AA%E5%B3%B0\\iTRAQ\\%E5%8D%8E%E5%A4%A7%E5%9F%BA%E5%9B%A0\\%E5%8D%8E%E5%A4%A7%E6%95%B0%E6%8D%AE\\%E8%9B%8B%E7%99%BD%E8%B4%A8%E7%BB%84%E4%B8%8E%E8%BD%AC%E5%BD%95%E7%BB%84%E5%85%B3%E8%81%94%E5%88%86%E6%9E%90\\F15FTSNCKF3616\\F15FTSNCKF3616\\Correlation\\SubCorrelation2Function\\Pathway\\SD_82-VS-SD_59_Cor_DEPs_Pathway\\SD_82-VS-SD_59_Cor_DEPs.htm#gene12#gene12) | 5 (2.94%) | ko00630 |
| 13 | [RNA transport](file:///E:\\%E7%8E%8B%E9%9B%AA%E5%B3%B0\\iTRAQ\\%E5%8D%8E%E5%A4%A7%E5%9F%BA%E5%9B%A0\\%E5%8D%8E%E5%A4%A7%E6%95%B0%E6%8D%AE\\%E8%9B%8B%E7%99%BD%E8%B4%A8%E7%BB%84%E4%B8%8E%E8%BD%AC%E5%BD%95%E7%BB%84%E5%85%B3%E8%81%94%E5%88%86%E6%9E%90\\F15FTSNCKF3616\\F15FTSNCKF3616\\Correlation\\SubCorrelation2Function\\Pathway\\SD_82-VS-SD_59_Cor_DEPs_Pathway\\SD_82-VS-SD_59_Cor_DEPs.htm#gene13#gene13) | 5 (2.94%) | ko03013 |
| 14 | [Protein processing in endoplasmic reticulum](file:///E:\\%E7%8E%8B%E9%9B%AA%E5%B3%B0\\iTRAQ\\%E5%8D%8E%E5%A4%A7%E5%9F%BA%E5%9B%A0\\%E5%8D%8E%E5%A4%A7%E6%95%B0%E6%8D%AE\\%E8%9B%8B%E7%99%BD%E8%B4%A8%E7%BB%84%E4%B8%8E%E8%BD%AC%E5%BD%95%E7%BB%84%E5%85%B3%E8%81%94%E5%88%86%E6%9E%90\\F15FTSNCKF3616\\F15FTSNCKF3616\\Correlation\\SubCorrelation2Function\\Pathway\\SD_82-VS-SD_59_Cor_DEPs_Pathway\\SD_82-VS-SD_59_Cor_DEPs.htm#gene14#gene14) | 5 (2.94%) | ko04141 |
| 15 | [Glycine, serine and threonine metabolism](file:///E:\\%E7%8E%8B%E9%9B%AA%E5%B3%B0\\iTRAQ\\%E5%8D%8E%E5%A4%A7%E5%9F%BA%E5%9B%A0\\%E5%8D%8E%E5%A4%A7%E6%95%B0%E6%8D%AE\\%E8%9B%8B%E7%99%BD%E8%B4%A8%E7%BB%84%E4%B8%8E%E8%BD%AC%E5%BD%95%E7%BB%84%E5%85%B3%E8%81%94%E5%88%86%E6%9E%90\\F15FTSNCKF3616\\F15FTSNCKF3616\\Correlation\\SubCorrelation2Function\\Pathway\\SD_82-VS-SD_59_Cor_DEPs_Pathway\\SD_82-VS-SD_59_Cor_DEPs.htm#gene15#gene15) | 5 (2.94%) | ko00260 |
| 16 | [Plant hormone signal transduction](file:///E:\\%E7%8E%8B%E9%9B%AA%E5%B3%B0\\iTRAQ\\%E5%8D%8E%E5%A4%A7%E5%9F%BA%E5%9B%A0\\%E5%8D%8E%E5%A4%A7%E6%95%B0%E6%8D%AE\\%E8%9B%8B%E7%99%BD%E8%B4%A8%E7%BB%84%E4%B8%8E%E8%BD%AC%E5%BD%95%E7%BB%84%E5%85%B3%E8%81%94%E5%88%86%E6%9E%90\\F15FTSNCKF3616\\F15FTSNCKF3616\\Correlation\\SubCorrelation2Function\\Pathway\\SD_82-VS-SD_59_Cor_DEPs_Pathway\\SD_82-VS-SD_59_Cor_DEPs.htm#gene16#gene16) | 4 (2.35%) | ko04075 |
| 17 | [Terpenoid backbone biosynthesis](file:///E:\\%E7%8E%8B%E9%9B%AA%E5%B3%B0\\iTRAQ\\%E5%8D%8E%E5%A4%A7%E5%9F%BA%E5%9B%A0\\%E5%8D%8E%E5%A4%A7%E6%95%B0%E6%8D%AE\\%E8%9B%8B%E7%99%BD%E8%B4%A8%E7%BB%84%E4%B8%8E%E8%BD%AC%E5%BD%95%E7%BB%84%E5%85%B3%E8%81%94%E5%88%86%E6%9E%90\\F15FTSNCKF3616\\F15FTSNCKF3616\\Correlation\\SubCorrelation2Function\\Pathway\\SD_82-VS-SD_59_Cor_DEPs_Pathway\\SD_82-VS-SD_59_Cor_DEPs.htm#gene17#gene17) | 4 (2.35%) | ko00900 |
| 18 | [Glycolysis / Gluconeogenesis](file:///E:\\%E7%8E%8B%E9%9B%AA%E5%B3%B0\\iTRAQ\\%E5%8D%8E%E5%A4%A7%E5%9F%BA%E5%9B%A0\\%E5%8D%8E%E5%A4%A7%E6%95%B0%E6%8D%AE\\%E8%9B%8B%E7%99%BD%E8%B4%A8%E7%BB%84%E4%B8%8E%E8%BD%AC%E5%BD%95%E7%BB%84%E5%85%B3%E8%81%94%E5%88%86%E6%9E%90\\F15FTSNCKF3616\\F15FTSNCKF3616\\Correlation\\SubCorrelation2Function\\Pathway\\SD_82-VS-SD_59_Cor_DEPs_Pathway\\SD_82-VS-SD_59_Cor_DEPs.htm#gene18#gene18) | 4 (2.35%) | ko00010 |
| 19 | [DNA replication](file:///E:\\%E7%8E%8B%E9%9B%AA%E5%B3%B0\\iTRAQ\\%E5%8D%8E%E5%A4%A7%E5%9F%BA%E5%9B%A0\\%E5%8D%8E%E5%A4%A7%E6%95%B0%E6%8D%AE\\%E8%9B%8B%E7%99%BD%E8%B4%A8%E7%BB%84%E4%B8%8E%E8%BD%AC%E5%BD%95%E7%BB%84%E5%85%B3%E8%81%94%E5%88%86%E6%9E%90\\F15FTSNCKF3616\\F15FTSNCKF3616\\Correlation\\SubCorrelation2Function\\Pathway\\SD_82-VS-SD_59_Cor_DEPs_Pathway\\SD_82-VS-SD_59_Cor_DEPs.htm#gene19#gene19) | 4 (2.35%) | ko03030 |
| 20 | [Galactose metabolism](file:///E:\\%E7%8E%8B%E9%9B%AA%E5%B3%B0\\iTRAQ\\%E5%8D%8E%E5%A4%A7%E5%9F%BA%E5%9B%A0\\%E5%8D%8E%E5%A4%A7%E6%95%B0%E6%8D%AE\\%E8%9B%8B%E7%99%BD%E8%B4%A8%E7%BB%84%E4%B8%8E%E8%BD%AC%E5%BD%95%E7%BB%84%E5%85%B3%E8%81%94%E5%88%86%E6%9E%90\\F15FTSNCKF3616\\F15FTSNCKF3616\\Correlation\\SubCorrelation2Function\\Pathway\\SD_82-VS-SD_59_Cor_DEPs_Pathway\\SD_82-VS-SD_59_Cor_DEPs.htm#gene20#gene20) | 4 (2.35%) | ko00052 |
| 21 | [Ribosome](file:///E:\\%E7%8E%8B%E9%9B%AA%E5%B3%B0\\iTRAQ\\%E5%8D%8E%E5%A4%A7%E5%9F%BA%E5%9B%A0\\%E5%8D%8E%E5%A4%A7%E6%95%B0%E6%8D%AE\\%E8%9B%8B%E7%99%BD%E8%B4%A8%E7%BB%84%E4%B8%8E%E8%BD%AC%E5%BD%95%E7%BB%84%E5%85%B3%E8%81%94%E5%88%86%E6%9E%90\\F15FTSNCKF3616\\F15FTSNCKF3616\\Correlation\\SubCorrelation2Function\\Pathway\\SD_82-VS-SD_59_Cor_DEPs_Pathway\\SD_82-VS-SD_59_Cor_DEPs.htm#gene21#gene21) | 4 (2.35%) | ko03010 |
| 22 | [Phagosome](file:///E:\\%E7%8E%8B%E9%9B%AA%E5%B3%B0\\iTRAQ\\%E5%8D%8E%E5%A4%A7%E5%9F%BA%E5%9B%A0\\%E5%8D%8E%E5%A4%A7%E6%95%B0%E6%8D%AE\\%E8%9B%8B%E7%99%BD%E8%B4%A8%E7%BB%84%E4%B8%8E%E8%BD%AC%E5%BD%95%E7%BB%84%E5%85%B3%E8%81%94%E5%88%86%E6%9E%90\\F15FTSNCKF3616\\F15FTSNCKF3616\\Correlation\\SubCorrelation2Function\\Pathway\\SD_82-VS-SD_59_Cor_DEPs_Pathway\\SD_82-VS-SD_59_Cor_DEPs.htm#gene22#gene22) | 4 (2.35%) | ko04145 |
| 23 | [Pentose and glucuronate interconversions](file:///E:\\%E7%8E%8B%E9%9B%AA%E5%B3%B0\\iTRAQ\\%E5%8D%8E%E5%A4%A7%E5%9F%BA%E5%9B%A0\\%E5%8D%8E%E5%A4%A7%E6%95%B0%E6%8D%AE\\%E8%9B%8B%E7%99%BD%E8%B4%A8%E7%BB%84%E4%B8%8E%E8%BD%AC%E5%BD%95%E7%BB%84%E5%85%B3%E8%81%94%E5%88%86%E6%9E%90\\F15FTSNCKF3616\\F15FTSNCKF3616\\Correlation\\SubCorrelation2Function\\Pathway\\SD_82-VS-SD_59_Cor_DEPs_Pathway\\SD_82-VS-SD_59_Cor_DEPs.htm#gene23#gene23) | 4 (2.35%) | ko00040 |
| 24 | [Pyruvate metabolism](file:///E:\\%E7%8E%8B%E9%9B%AA%E5%B3%B0\\iTRAQ\\%E5%8D%8E%E5%A4%A7%E5%9F%BA%E5%9B%A0\\%E5%8D%8E%E5%A4%A7%E6%95%B0%E6%8D%AE\\%E8%9B%8B%E7%99%BD%E8%B4%A8%E7%BB%84%E4%B8%8E%E8%BD%AC%E5%BD%95%E7%BB%84%E5%85%B3%E8%81%94%E5%88%86%E6%9E%90\\F15FTSNCKF3616\\F15FTSNCKF3616\\Correlation\\SubCorrelation2Function\\Pathway\\SD_82-VS-SD_59_Cor_DEPs_Pathway\\SD_82-VS-SD_59_Cor_DEPs.htm#gene24#gene24) | 4 (2.35%) | ko00620 |
| 25 | [Cysteine and methionine metabolism](file:///E:\\%E7%8E%8B%E9%9B%AA%E5%B3%B0\\iTRAQ\\%E5%8D%8E%E5%A4%A7%E5%9F%BA%E5%9B%A0\\%E5%8D%8E%E5%A4%A7%E6%95%B0%E6%8D%AE\\%E8%9B%8B%E7%99%BD%E8%B4%A8%E7%BB%84%E4%B8%8E%E8%BD%AC%E5%BD%95%E7%BB%84%E5%85%B3%E8%81%94%E5%88%86%E6%9E%90\\F15FTSNCKF3616\\F15FTSNCKF3616\\Correlation\\SubCorrelation2Function\\Pathway\\SD_82-VS-SD_59_Cor_DEPs_Pathway\\SD_82-VS-SD_59_Cor_DEPs.htm#gene25#gene25) | 3 (1.76%) | ko00270 |
| 26 | [Other glycan degradation](file:///E:\\%E7%8E%8B%E9%9B%AA%E5%B3%B0\\iTRAQ\\%E5%8D%8E%E5%A4%A7%E5%9F%BA%E5%9B%A0\\%E5%8D%8E%E5%A4%A7%E6%95%B0%E6%8D%AE\\%E8%9B%8B%E7%99%BD%E8%B4%A8%E7%BB%84%E4%B8%8E%E8%BD%AC%E5%BD%95%E7%BB%84%E5%85%B3%E8%81%94%E5%88%86%E6%9E%90\\F15FTSNCKF3616\\F15FTSNCKF3616\\Correlation\\SubCorrelation2Function\\Pathway\\SD_82-VS-SD_59_Cor_DEPs_Pathway\\SD_82-VS-SD_59_Cor_DEPs.htm#gene26#gene26) | 3 (1.76%) | ko00511 |
| 27 | [Ascorbate and aldarate metabolism](file:///E:\\%E7%8E%8B%E9%9B%AA%E5%B3%B0\\iTRAQ\\%E5%8D%8E%E5%A4%A7%E5%9F%BA%E5%9B%A0\\%E5%8D%8E%E5%A4%A7%E6%95%B0%E6%8D%AE\\%E8%9B%8B%E7%99%BD%E8%B4%A8%E7%BB%84%E4%B8%8E%E8%BD%AC%E5%BD%95%E7%BB%84%E5%85%B3%E8%81%94%E5%88%86%E6%9E%90\\F15FTSNCKF3616\\F15FTSNCKF3616\\Correlation\\SubCorrelation2Function\\Pathway\\SD_82-VS-SD_59_Cor_DEPs_Pathway\\SD_82-VS-SD_59_Cor_DEPs.htm#gene27#gene27) | 3 (1.76%) | ko00053 |
| 28 | [Propanoate metabolism](file:///E:\\%E7%8E%8B%E9%9B%AA%E5%B3%B0\\iTRAQ\\%E5%8D%8E%E5%A4%A7%E5%9F%BA%E5%9B%A0\\%E5%8D%8E%E5%A4%A7%E6%95%B0%E6%8D%AE\\%E8%9B%8B%E7%99%BD%E8%B4%A8%E7%BB%84%E4%B8%8E%E8%BD%AC%E5%BD%95%E7%BB%84%E5%85%B3%E8%81%94%E5%88%86%E6%9E%90\\F15FTSNCKF3616\\F15FTSNCKF3616\\Correlation\\SubCorrelation2Function\\Pathway\\SD_82-VS-SD_59_Cor_DEPs_Pathway\\SD_82-VS-SD_59_Cor_DEPs.htm#gene28#gene28) | 3 (1.76%) | ko00640 |
| 29 | [Nucleotide excision repair](file:///E:\\%E7%8E%8B%E9%9B%AA%E5%B3%B0\\iTRAQ\\%E5%8D%8E%E5%A4%A7%E5%9F%BA%E5%9B%A0\\%E5%8D%8E%E5%A4%A7%E6%95%B0%E6%8D%AE\\%E8%9B%8B%E7%99%BD%E8%B4%A8%E7%BB%84%E4%B8%8E%E8%BD%AC%E5%BD%95%E7%BB%84%E5%85%B3%E8%81%94%E5%88%86%E6%9E%90\\F15FTSNCKF3616\\F15FTSNCKF3616\\Correlation\\SubCorrelation2Function\\Pathway\\SD_82-VS-SD_59_Cor_DEPs_Pathway\\SD_82-VS-SD_59_Cor_DEPs.htm#gene29#gene29) | 3 (1.76%) | ko03420 |
| 30 | [Glycerophospholipid metabolism](file:///E:\\%E7%8E%8B%E9%9B%AA%E5%B3%B0\\iTRAQ\\%E5%8D%8E%E5%A4%A7%E5%9F%BA%E5%9B%A0\\%E5%8D%8E%E5%A4%A7%E6%95%B0%E6%8D%AE\\%E8%9B%8B%E7%99%BD%E8%B4%A8%E7%BB%84%E4%B8%8E%E8%BD%AC%E5%BD%95%E7%BB%84%E5%85%B3%E8%81%94%E5%88%86%E6%9E%90\\F15FTSNCKF3616\\F15FTSNCKF3616\\Correlation\\SubCorrelation2Function\\Pathway\\SD_82-VS-SD_59_Cor_DEPs_Pathway\\SD_82-VS-SD_59_Cor_DEPs.htm#gene30#gene30) | 3 (1.76%) | ko00564 |
| 31 | [Valine, leucine and isoleucine degradation](file:///E:\\%E7%8E%8B%E9%9B%AA%E5%B3%B0\\iTRAQ\\%E5%8D%8E%E5%A4%A7%E5%9F%BA%E5%9B%A0\\%E5%8D%8E%E5%A4%A7%E6%95%B0%E6%8D%AE\\%E8%9B%8B%E7%99%BD%E8%B4%A8%E7%BB%84%E4%B8%8E%E8%BD%AC%E5%BD%95%E7%BB%84%E5%85%B3%E8%81%94%E5%88%86%E6%9E%90\\F15FTSNCKF3616\\F15FTSNCKF3616\\Correlation\\SubCorrelation2Function\\Pathway\\SD_82-VS-SD_59_Cor_DEPs_Pathway\\SD_82-VS-SD_59_Cor_DEPs.htm#gene31#gene31) | 3 (1.76%) | ko00280 |
| 32 | [Fatty acid metabolism](file:///E:\\%E7%8E%8B%E9%9B%AA%E5%B3%B0\\iTRAQ\\%E5%8D%8E%E5%A4%A7%E5%9F%BA%E5%9B%A0\\%E5%8D%8E%E5%A4%A7%E6%95%B0%E6%8D%AE\\%E8%9B%8B%E7%99%BD%E8%B4%A8%E7%BB%84%E4%B8%8E%E8%BD%AC%E5%BD%95%E7%BB%84%E5%85%B3%E8%81%94%E5%88%86%E6%9E%90\\F15FTSNCKF3616\\F15FTSNCKF3616\\Correlation\\SubCorrelation2Function\\Pathway\\SD_82-VS-SD_59_Cor_DEPs_Pathway\\SD_82-VS-SD_59_Cor_DEPs.htm#gene32#gene32) | 3 (1.76%) | ko01212 |
| 33 | [Cyanoamino acid metabolism](file:///E:\\%E7%8E%8B%E9%9B%AA%E5%B3%B0\\iTRAQ\\%E5%8D%8E%E5%A4%A7%E5%9F%BA%E5%9B%A0\\%E5%8D%8E%E5%A4%A7%E6%95%B0%E6%8D%AE\\%E8%9B%8B%E7%99%BD%E8%B4%A8%E7%BB%84%E4%B8%8E%E8%BD%AC%E5%BD%95%E7%BB%84%E5%85%B3%E8%81%94%E5%88%86%E6%9E%90\\F15FTSNCKF3616\\F15FTSNCKF3616\\Correlation\\SubCorrelation2Function\\Pathway\\SD_82-VS-SD_59_Cor_DEPs_Pathway\\SD_82-VS-SD_59_Cor_DEPs.htm#gene33#gene33) | 3 (1.76%) | ko00460 |
| 34 | [Purine metabolism](file:///E:\\%E7%8E%8B%E9%9B%AA%E5%B3%B0\\iTRAQ\\%E5%8D%8E%E5%A4%A7%E5%9F%BA%E5%9B%A0\\%E5%8D%8E%E5%A4%A7%E6%95%B0%E6%8D%AE\\%E8%9B%8B%E7%99%BD%E8%B4%A8%E7%BB%84%E4%B8%8E%E8%BD%AC%E5%BD%95%E7%BB%84%E5%85%B3%E8%81%94%E5%88%86%E6%9E%90\\F15FTSNCKF3616\\F15FTSNCKF3616\\Correlation\\SubCorrelation2Function\\Pathway\\SD_82-VS-SD_59_Cor_DEPs_Pathway\\SD_82-VS-SD_59_Cor_DEPs.htm#gene34#gene34) | 2 (1.18%) | ko00230 |
| 35 | [Isoflavonoid biosynthesis](file:///E:\\%E7%8E%8B%E9%9B%AA%E5%B3%B0\\iTRAQ\\%E5%8D%8E%E5%A4%A7%E5%9F%BA%E5%9B%A0\\%E5%8D%8E%E5%A4%A7%E6%95%B0%E6%8D%AE\\%E8%9B%8B%E7%99%BD%E8%B4%A8%E7%BB%84%E4%B8%8E%E8%BD%AC%E5%BD%95%E7%BB%84%E5%85%B3%E8%81%94%E5%88%86%E6%9E%90\\F15FTSNCKF3616\\F15FTSNCKF3616\\Correlation\\SubCorrelation2Function\\Pathway\\SD_82-VS-SD_59_Cor_DEPs_Pathway\\SD_82-VS-SD_59_Cor_DEPs.htm#gene35#gene35) | 2 (1.18%) | ko00943 |
| 36 | [Mismatch repair](file:///E:\\%E7%8E%8B%E9%9B%AA%E5%B3%B0\\iTRAQ\\%E5%8D%8E%E5%A4%A7%E5%9F%BA%E5%9B%A0\\%E5%8D%8E%E5%A4%A7%E6%95%B0%E6%8D%AE\\%E8%9B%8B%E7%99%BD%E8%B4%A8%E7%BB%84%E4%B8%8E%E8%BD%AC%E5%BD%95%E7%BB%84%E5%85%B3%E8%81%94%E5%88%86%E6%9E%90\\F15FTSNCKF3616\\F15FTSNCKF3616\\Correlation\\SubCorrelation2Function\\Pathway\\SD_82-VS-SD_59_Cor_DEPs_Pathway\\SD_82-VS-SD_59_Cor_DEPs.htm#gene36#gene36) | 2 (1.18%) | ko03430 |
| 37 | [Lysine degradation](file:///E:\\%E7%8E%8B%E9%9B%AA%E5%B3%B0\\iTRAQ\\%E5%8D%8E%E5%A4%A7%E5%9F%BA%E5%9B%A0\\%E5%8D%8E%E5%A4%A7%E6%95%B0%E6%8D%AE\\%E8%9B%8B%E7%99%BD%E8%B4%A8%E7%BB%84%E4%B8%8E%E8%BD%AC%E5%BD%95%E7%BB%84%E5%85%B3%E8%81%94%E5%88%86%E6%9E%90\\F15FTSNCKF3616\\F15FTSNCKF3616\\Correlation\\SubCorrelation2Function\\Pathway\\SD_82-VS-SD_59_Cor_DEPs_Pathway\\SD_82-VS-SD_59_Cor_DEPs.htm#gene37#gene37) | 2 (1.18%) | ko00310 |
| 38 | [alpha-Linolenic acid metabolism](file:///E:\\%E7%8E%8B%E9%9B%AA%E5%B3%B0\\iTRAQ\\%E5%8D%8E%E5%A4%A7%E5%9F%BA%E5%9B%A0\\%E5%8D%8E%E5%A4%A7%E6%95%B0%E6%8D%AE\\%E8%9B%8B%E7%99%BD%E8%B4%A8%E7%BB%84%E4%B8%8E%E8%BD%AC%E5%BD%95%E7%BB%84%E5%85%B3%E8%81%94%E5%88%86%E6%9E%90\\F15FTSNCKF3616\\F15FTSNCKF3616\\Correlation\\SubCorrelation2Function\\Pathway\\SD_82-VS-SD_59_Cor_DEPs_Pathway\\SD_82-VS-SD_59_Cor_DEPs.htm#gene38#gene38) | 2 (1.18%) | ko00592 |
| 39 | [Arachidonic acid metabolism](file:///E:\\%E7%8E%8B%E9%9B%AA%E5%B3%B0\\iTRAQ\\%E5%8D%8E%E5%A4%A7%E5%9F%BA%E5%9B%A0\\%E5%8D%8E%E5%A4%A7%E6%95%B0%E6%8D%AE\\%E8%9B%8B%E7%99%BD%E8%B4%A8%E7%BB%84%E4%B8%8E%E8%BD%AC%E5%BD%95%E7%BB%84%E5%85%B3%E8%81%94%E5%88%86%E6%9E%90\\F15FTSNCKF3616\\F15FTSNCKF3616\\Correlation\\SubCorrelation2Function\\Pathway\\SD_82-VS-SD_59_Cor_DEPs_Pathway\\SD_82-VS-SD_59_Cor_DEPs.htm#gene39#gene39) | 2 (1.18%) | ko00590 |
| 40 | [Fatty acid degradation](file:///E:\\%E7%8E%8B%E9%9B%AA%E5%B3%B0\\iTRAQ\\%E5%8D%8E%E5%A4%A7%E5%9F%BA%E5%9B%A0\\%E5%8D%8E%E5%A4%A7%E6%95%B0%E6%8D%AE\\%E8%9B%8B%E7%99%BD%E8%B4%A8%E7%BB%84%E4%B8%8E%E8%BD%AC%E5%BD%95%E7%BB%84%E5%85%B3%E8%81%94%E5%88%86%E6%9E%90\\F15FTSNCKF3616\\F15FTSNCKF3616\\Correlation\\SubCorrelation2Function\\Pathway\\SD_82-VS-SD_59_Cor_DEPs_Pathway\\SD_82-VS-SD_59_Cor_DEPs.htm#gene40#gene40) | 2 (1.18%) | ko00071 |
| 41 | [Spliceosome](file:///E:\\%E7%8E%8B%E9%9B%AA%E5%B3%B0\\iTRAQ\\%E5%8D%8E%E5%A4%A7%E5%9F%BA%E5%9B%A0\\%E5%8D%8E%E5%A4%A7%E6%95%B0%E6%8D%AE\\%E8%9B%8B%E7%99%BD%E8%B4%A8%E7%BB%84%E4%B8%8E%E8%BD%AC%E5%BD%95%E7%BB%84%E5%85%B3%E8%81%94%E5%88%86%E6%9E%90\\F15FTSNCKF3616\\F15FTSNCKF3616\\Correlation\\SubCorrelation2Function\\Pathway\\SD_82-VS-SD_59_Cor_DEPs_Pathway\\SD_82-VS-SD_59_Cor_DEPs.htm#gene41#gene41) | 2 (1.18%) | ko03040 |
| 42 | [Butanoate metabolism](file:///E:\\%E7%8E%8B%E9%9B%AA%E5%B3%B0\\iTRAQ\\%E5%8D%8E%E5%A4%A7%E5%9F%BA%E5%9B%A0\\%E5%8D%8E%E5%A4%A7%E6%95%B0%E6%8D%AE\\%E8%9B%8B%E7%99%BD%E8%B4%A8%E7%BB%84%E4%B8%8E%E8%BD%AC%E5%BD%95%E7%BB%84%E5%85%B3%E8%81%94%E5%88%86%E6%9E%90\\F15FTSNCKF3616\\F15FTSNCKF3616\\Correlation\\SubCorrelation2Function\\Pathway\\SD_82-VS-SD_59_Cor_DEPs_Pathway\\SD_82-VS-SD_59_Cor_DEPs.htm#gene42#gene42) | 2 (1.18%) | ko00650 |
| 43 | [mRNA surveillance pathway](file:///E:\\%E7%8E%8B%E9%9B%AA%E5%B3%B0\\iTRAQ\\%E5%8D%8E%E5%A4%A7%E5%9F%BA%E5%9B%A0\\%E5%8D%8E%E5%A4%A7%E6%95%B0%E6%8D%AE\\%E8%9B%8B%E7%99%BD%E8%B4%A8%E7%BB%84%E4%B8%8E%E8%BD%AC%E5%BD%95%E7%BB%84%E5%85%B3%E8%81%94%E5%88%86%E6%9E%90\\F15FTSNCKF3616\\F15FTSNCKF3616\\Correlation\\SubCorrelation2Function\\Pathway\\SD_82-VS-SD_59_Cor_DEPs_Pathway\\SD_82-VS-SD_59_Cor_DEPs.htm#gene43#gene43) | 2 (1.18%) | ko03015 |
| 44 | [Nitrogen metabolism](file:///E:\\%E7%8E%8B%E9%9B%AA%E5%B3%B0\\iTRAQ\\%E5%8D%8E%E5%A4%A7%E5%9F%BA%E5%9B%A0\\%E5%8D%8E%E5%A4%A7%E6%95%B0%E6%8D%AE\\%E8%9B%8B%E7%99%BD%E8%B4%A8%E7%BB%84%E4%B8%8E%E8%BD%AC%E5%BD%95%E7%BB%84%E5%85%B3%E8%81%94%E5%88%86%E6%9E%90\\F15FTSNCKF3616\\F15FTSNCKF3616\\Correlation\\SubCorrelation2Function\\Pathway\\SD_82-VS-SD_59_Cor_DEPs_Pathway\\SD_82-VS-SD_59_Cor_DEPs.htm#gene44#gene44) | 2 (1.18%) | ko00910 |
| 45 | [Aminoacyl-tRNA biosynthesis](file:///E:\\%E7%8E%8B%E9%9B%AA%E5%B3%B0\\iTRAQ\\%E5%8D%8E%E5%A4%A7%E5%9F%BA%E5%9B%A0\\%E5%8D%8E%E5%A4%A7%E6%95%B0%E6%8D%AE\\%E8%9B%8B%E7%99%BD%E8%B4%A8%E7%BB%84%E4%B8%8E%E8%BD%AC%E5%BD%95%E7%BB%84%E5%85%B3%E8%81%94%E5%88%86%E6%9E%90\\F15FTSNCKF3616\\F15FTSNCKF3616\\Correlation\\SubCorrelation2Function\\Pathway\\SD_82-VS-SD_59_Cor_DEPs_Pathway\\SD_82-VS-SD_59_Cor_DEPs.htm#gene45#gene45) | 2 (1.18%) | ko00970 |
| 46 | [Sphingolipid metabolism](file:///E:\\%E7%8E%8B%E9%9B%AA%E5%B3%B0\\iTRAQ\\%E5%8D%8E%E5%A4%A7%E5%9F%BA%E5%9B%A0\\%E5%8D%8E%E5%A4%A7%E6%95%B0%E6%8D%AE\\%E8%9B%8B%E7%99%BD%E8%B4%A8%E7%BB%84%E4%B8%8E%E8%BD%AC%E5%BD%95%E7%BB%84%E5%85%B3%E8%81%94%E5%88%86%E6%9E%90\\F15FTSNCKF3616\\F15FTSNCKF3616\\Correlation\\SubCorrelation2Function\\Pathway\\SD_82-VS-SD_59_Cor_DEPs_Pathway\\SD_82-VS-SD_59_Cor_DEPs.htm#gene46#gene46) | 2 (1.18%) | ko00600 |
| 47 | [Alanine, aspartate and glutamate metabolism](file:///E:\\%E7%8E%8B%E9%9B%AA%E5%B3%B0\\iTRAQ\\%E5%8D%8E%E5%A4%A7%E5%9F%BA%E5%9B%A0\\%E5%8D%8E%E5%A4%A7%E6%95%B0%E6%8D%AE\\%E8%9B%8B%E7%99%BD%E8%B4%A8%E7%BB%84%E4%B8%8E%E8%BD%AC%E5%BD%95%E7%BB%84%E5%85%B3%E8%81%94%E5%88%86%E6%9E%90\\F15FTSNCKF3616\\F15FTSNCKF3616\\Correlation\\SubCorrelation2Function\\Pathway\\SD_82-VS-SD_59_Cor_DEPs_Pathway\\SD_82-VS-SD_59_Cor_DEPs.htm#gene47#gene47) | 2 (1.18%) | ko00250 |
| 48 | [Tryptophan metabolism](file:///E:\\%E7%8E%8B%E9%9B%AA%E5%B3%B0\\iTRAQ\\%E5%8D%8E%E5%A4%A7%E5%9F%BA%E5%9B%A0\\%E5%8D%8E%E5%A4%A7%E6%95%B0%E6%8D%AE\\%E8%9B%8B%E7%99%BD%E8%B4%A8%E7%BB%84%E4%B8%8E%E8%BD%AC%E5%BD%95%E7%BB%84%E5%85%B3%E8%81%94%E5%88%86%E6%9E%90\\F15FTSNCKF3616\\F15FTSNCKF3616\\Correlation\\SubCorrelation2Function\\Pathway\\SD_82-VS-SD_59_Cor_DEPs_Pathway\\SD_82-VS-SD_59_Cor_DEPs.htm#gene48#gene48) | 2 (1.18%) | ko00380 |
| 49 | [Citrate cycle (TCA cycle)](file:///E:\\%E7%8E%8B%E9%9B%AA%E5%B3%B0\\iTRAQ\\%E5%8D%8E%E5%A4%A7%E5%9F%BA%E5%9B%A0\\%E5%8D%8E%E5%A4%A7%E6%95%B0%E6%8D%AE\\%E8%9B%8B%E7%99%BD%E8%B4%A8%E7%BB%84%E4%B8%8E%E8%BD%AC%E5%BD%95%E7%BB%84%E5%85%B3%E8%81%94%E5%88%86%E6%9E%90\\F15FTSNCKF3616\\F15FTSNCKF3616\\Correlation\\SubCorrelation2Function\\Pathway\\SD_82-VS-SD_59_Cor_DEPs_Pathway\\SD_82-VS-SD_59_Cor_DEPs.htm#gene49#gene49) | 2 (1.18%) | ko00020 |
| 50 | [Glycerolipid metabolism](file:///E:\\%E7%8E%8B%E9%9B%AA%E5%B3%B0\\iTRAQ\\%E5%8D%8E%E5%A4%A7%E5%9F%BA%E5%9B%A0\\%E5%8D%8E%E5%A4%A7%E6%95%B0%E6%8D%AE\\%E8%9B%8B%E7%99%BD%E8%B4%A8%E7%BB%84%E4%B8%8E%E8%BD%AC%E5%BD%95%E7%BB%84%E5%85%B3%E8%81%94%E5%88%86%E6%9E%90\\F15FTSNCKF3616\\F15FTSNCKF3616\\Correlation\\SubCorrelation2Function\\Pathway\\SD_82-VS-SD_59_Cor_DEPs_Pathway\\SD_82-VS-SD_59_Cor_DEPs.htm#gene50#gene50) | 2 (1.18%) | ko00561 |
| 51 | [Synthesis and degradation of ketone bodies](file:///E:\\%E7%8E%8B%E9%9B%AA%E5%B3%B0\\iTRAQ\\%E5%8D%8E%E5%A4%A7%E5%9F%BA%E5%9B%A0\\%E5%8D%8E%E5%A4%A7%E6%95%B0%E6%8D%AE\\%E8%9B%8B%E7%99%BD%E8%B4%A8%E7%BB%84%E4%B8%8E%E8%BD%AC%E5%BD%95%E7%BB%84%E5%85%B3%E8%81%94%E5%88%86%E6%9E%90\\F15FTSNCKF3616\\F15FTSNCKF3616\\Correlation\\SubCorrelation2Function\\Pathway\\SD_82-VS-SD_59_Cor_DEPs_Pathway\\SD_82-VS-SD_59_Cor_DEPs.htm#gene51#gene51) | 2 (1.18%) | ko00072 |
| 52 | [Proteasome](file:///E:\\%E7%8E%8B%E9%9B%AA%E5%B3%B0\\iTRAQ\\%E5%8D%8E%E5%A4%A7%E5%9F%BA%E5%9B%A0\\%E5%8D%8E%E5%A4%A7%E6%95%B0%E6%8D%AE\\%E8%9B%8B%E7%99%BD%E8%B4%A8%E7%BB%84%E4%B8%8E%E8%BD%AC%E5%BD%95%E7%BB%84%E5%85%B3%E8%81%94%E5%88%86%E6%9E%90\\F15FTSNCKF3616\\F15FTSNCKF3616\\Correlation\\SubCorrelation2Function\\Pathway\\SD_82-VS-SD_59_Cor_DEPs_Pathway\\SD_82-VS-SD_59_Cor_DEPs.htm#gene52#gene52) | 2 (1.18%) | ko03050 |
| 53 | [Fructose and mannose metabolism](file:///E:\\%E7%8E%8B%E9%9B%AA%E5%B3%B0\\iTRAQ\\%E5%8D%8E%E5%A4%A7%E5%9F%BA%E5%9B%A0\\%E5%8D%8E%E5%A4%A7%E6%95%B0%E6%8D%AE\\%E8%9B%8B%E7%99%BD%E8%B4%A8%E7%BB%84%E4%B8%8E%E8%BD%AC%E5%BD%95%E7%BB%84%E5%85%B3%E8%81%94%E5%88%86%E6%9E%90\\F15FTSNCKF3616\\F15FTSNCKF3616\\Correlation\\SubCorrelation2Function\\Pathway\\SD_82-VS-SD_59_Cor_DEPs_Pathway\\SD_82-VS-SD_59_Cor_DEPs.htm#gene53#gene53) | 2 (1.18%) | ko00051 |
| 54 | [Base excision repair](file:///E:\\%E7%8E%8B%E9%9B%AA%E5%B3%B0\\iTRAQ\\%E5%8D%8E%E5%A4%A7%E5%9F%BA%E5%9B%A0\\%E5%8D%8E%E5%A4%A7%E6%95%B0%E6%8D%AE\\%E8%9B%8B%E7%99%BD%E8%B4%A8%E7%BB%84%E4%B8%8E%E8%BD%AC%E5%BD%95%E7%BB%84%E5%85%B3%E8%81%94%E5%88%86%E6%9E%90\\F15FTSNCKF3616\\F15FTSNCKF3616\\Correlation\\SubCorrelation2Function\\Pathway\\SD_82-VS-SD_59_Cor_DEPs_Pathway\\SD_82-VS-SD_59_Cor_DEPs.htm#gene54#gene54) | 2 (1.18%) | ko03410 |
| 55 | [Pentose phosphate pathway](file:///E:\\%E7%8E%8B%E9%9B%AA%E5%B3%B0\\iTRAQ\\%E5%8D%8E%E5%A4%A7%E5%9F%BA%E5%9B%A0\\%E5%8D%8E%E5%A4%A7%E6%95%B0%E6%8D%AE\\%E8%9B%8B%E7%99%BD%E8%B4%A8%E7%BB%84%E4%B8%8E%E8%BD%AC%E5%BD%95%E7%BB%84%E5%85%B3%E8%81%94%E5%88%86%E6%9E%90\\F15FTSNCKF3616\\F15FTSNCKF3616\\Correlation\\SubCorrelation2Function\\Pathway\\SD_82-VS-SD_59_Cor_DEPs_Pathway\\SD_82-VS-SD_59_Cor_DEPs.htm#gene55#gene55) | 1 (0.59%) | ko00030 |
| 56 | [Homologous recombination](file:///E:\\%E7%8E%8B%E9%9B%AA%E5%B3%B0\\iTRAQ\\%E5%8D%8E%E5%A4%A7%E5%9F%BA%E5%9B%A0\\%E5%8D%8E%E5%A4%A7%E6%95%B0%E6%8D%AE\\%E8%9B%8B%E7%99%BD%E8%B4%A8%E7%BB%84%E4%B8%8E%E8%BD%AC%E5%BD%95%E7%BB%84%E5%85%B3%E8%81%94%E5%88%86%E6%9E%90\\F15FTSNCKF3616\\F15FTSNCKF3616\\Correlation\\SubCorrelation2Function\\Pathway\\SD_82-VS-SD_59_Cor_DEPs_Pathway\\SD_82-VS-SD_59_Cor_DEPs.htm#gene56#gene56) | 1 (0.59%) | ko03440 |
| 57 | [Fatty acid biosynthesis](file:///E:\\%E7%8E%8B%E9%9B%AA%E5%B3%B0\\iTRAQ\\%E5%8D%8E%E5%A4%A7%E5%9F%BA%E5%9B%A0\\%E5%8D%8E%E5%A4%A7%E6%95%B0%E6%8D%AE\\%E8%9B%8B%E7%99%BD%E8%B4%A8%E7%BB%84%E4%B8%8E%E8%BD%AC%E5%BD%95%E7%BB%84%E5%85%B3%E8%81%94%E5%88%86%E6%9E%90\\F15FTSNCKF3616\\F15FTSNCKF3616\\Correlation\\SubCorrelation2Function\\Pathway\\SD_82-VS-SD_59_Cor_DEPs_Pathway\\SD_82-VS-SD_59_Cor_DEPs.htm#gene57#gene57) | 1 (0.59%) | ko00061 |
| 58 | [Flavonoid biosynthesis](file:///E:\\%E7%8E%8B%E9%9B%AA%E5%B3%B0\\iTRAQ\\%E5%8D%8E%E5%A4%A7%E5%9F%BA%E5%9B%A0\\%E5%8D%8E%E5%A4%A7%E6%95%B0%E6%8D%AE\\%E8%9B%8B%E7%99%BD%E8%B4%A8%E7%BB%84%E4%B8%8E%E8%BD%AC%E5%BD%95%E7%BB%84%E5%85%B3%E8%81%94%E5%88%86%E6%9E%90\\F15FTSNCKF3616\\F15FTSNCKF3616\\Correlation\\SubCorrelation2Function\\Pathway\\SD_82-VS-SD_59_Cor_DEPs_Pathway\\SD_82-VS-SD_59_Cor_DEPs.htm#gene58#gene58) | 1 (0.59%) | ko00941 |
| 59 | [Glycosphingolipid biosynthesis - ganglio series](file:///E:\\%E7%8E%8B%E9%9B%AA%E5%B3%B0\\iTRAQ\\%E5%8D%8E%E5%A4%A7%E5%9F%BA%E5%9B%A0\\%E5%8D%8E%E5%A4%A7%E6%95%B0%E6%8D%AE\\%E8%9B%8B%E7%99%BD%E8%B4%A8%E7%BB%84%E4%B8%8E%E8%BD%AC%E5%BD%95%E7%BB%84%E5%85%B3%E8%81%94%E5%88%86%E6%9E%90\\F15FTSNCKF3616\\F15FTSNCKF3616\\Correlation\\SubCorrelation2Function\\Pathway\\SD_82-VS-SD_59_Cor_DEPs_Pathway\\SD_82-VS-SD_59_Cor_DEPs.htm#gene59#gene59) | 1 (0.59%) | ko00604 |
| 60 | [Linoleic acid metabolism](file:///E:\\%E7%8E%8B%E9%9B%AA%E5%B3%B0\\iTRAQ\\%E5%8D%8E%E5%A4%A7%E5%9F%BA%E5%9B%A0\\%E5%8D%8E%E5%A4%A7%E6%95%B0%E6%8D%AE\\%E8%9B%8B%E7%99%BD%E8%B4%A8%E7%BB%84%E4%B8%8E%E8%BD%AC%E5%BD%95%E7%BB%84%E5%85%B3%E8%81%94%E5%88%86%E6%9E%90\\F15FTSNCKF3616\\F15FTSNCKF3616\\Correlation\\SubCorrelation2Function\\Pathway\\SD_82-VS-SD_59_Cor_DEPs_Pathway\\SD_82-VS-SD_59_Cor_DEPs.htm#gene60#gene60) | 1 (0.59%) | ko00591 |
| 61 | [Oxidative phosphorylation](file:///E:\\%E7%8E%8B%E9%9B%AA%E5%B3%B0\\iTRAQ\\%E5%8D%8E%E5%A4%A7%E5%9F%BA%E5%9B%A0\\%E5%8D%8E%E5%A4%A7%E6%95%B0%E6%8D%AE\\%E8%9B%8B%E7%99%BD%E8%B4%A8%E7%BB%84%E4%B8%8E%E8%BD%AC%E5%BD%95%E7%BB%84%E5%85%B3%E8%81%94%E5%88%86%E6%9E%90\\F15FTSNCKF3616\\F15FTSNCKF3616\\Correlation\\SubCorrelation2Function\\Pathway\\SD_82-VS-SD_59_Cor_DEPs_Pathway\\SD_82-VS-SD_59_Cor_DEPs.htm#gene61#gene61) | 1 (0.59%) | ko00190 |
| 62 | [Anthocyanin biosynthesis](file:///E:\\%E7%8E%8B%E9%9B%AA%E5%B3%B0\\iTRAQ\\%E5%8D%8E%E5%A4%A7%E5%9F%BA%E5%9B%A0\\%E5%8D%8E%E5%A4%A7%E6%95%B0%E6%8D%AE\\%E8%9B%8B%E7%99%BD%E8%B4%A8%E7%BB%84%E4%B8%8E%E8%BD%AC%E5%BD%95%E7%BB%84%E5%85%B3%E8%81%94%E5%88%86%E6%9E%90\\F15FTSNCKF3616\\F15FTSNCKF3616\\Correlation\\SubCorrelation2Function\\Pathway\\SD_82-VS-SD_59_Cor_DEPs_Pathway\\SD_82-VS-SD_59_Cor_DEPs.htm#gene62#gene62) | 1 (0.59%) | ko00942 |
| 63 | [Betalain biosynthesis](file:///E:\\%E7%8E%8B%E9%9B%AA%E5%B3%B0\\iTRAQ\\%E5%8D%8E%E5%A4%A7%E5%9F%BA%E5%9B%A0\\%E5%8D%8E%E5%A4%A7%E6%95%B0%E6%8D%AE\\%E8%9B%8B%E7%99%BD%E8%B4%A8%E7%BB%84%E4%B8%8E%E8%BD%AC%E5%BD%95%E7%BB%84%E5%85%B3%E8%81%94%E5%88%86%E6%9E%90\\F15FTSNCKF3616\\F15FTSNCKF3616\\Correlation\\SubCorrelation2Function\\Pathway\\SD_82-VS-SD_59_Cor_DEPs_Pathway\\SD_82-VS-SD_59_Cor_DEPs.htm#gene63#gene63) | 1 (0.59%) | ko00965 |
| 64 | [Carotenoid biosynthesis](file:///E:\\%E7%8E%8B%E9%9B%AA%E5%B3%B0\\iTRAQ\\%E5%8D%8E%E5%A4%A7%E5%9F%BA%E5%9B%A0\\%E5%8D%8E%E5%A4%A7%E6%95%B0%E6%8D%AE\\%E8%9B%8B%E7%99%BD%E8%B4%A8%E7%BB%84%E4%B8%8E%E8%BD%AC%E5%BD%95%E7%BB%84%E5%85%B3%E8%81%94%E5%88%86%E6%9E%90\\F15FTSNCKF3616\\F15FTSNCKF3616\\Correlation\\SubCorrelation2Function\\Pathway\\SD_82-VS-SD_59_Cor_DEPs_Pathway\\SD_82-VS-SD_59_Cor_DEPs.htm#gene64#gene64) | 1 (0.59%) | ko00906 |
| 65 | [Protein export](file:///E:\\%E7%8E%8B%E9%9B%AA%E5%B3%B0\\iTRAQ\\%E5%8D%8E%E5%A4%A7%E5%9F%BA%E5%9B%A0\\%E5%8D%8E%E5%A4%A7%E6%95%B0%E6%8D%AE\\%E8%9B%8B%E7%99%BD%E8%B4%A8%E7%BB%84%E4%B8%8E%E8%BD%AC%E5%BD%95%E7%BB%84%E5%85%B3%E8%81%94%E5%88%86%E6%9E%90\\F15FTSNCKF3616\\F15FTSNCKF3616\\Correlation\\SubCorrelation2Function\\Pathway\\SD_82-VS-SD_59_Cor_DEPs_Pathway\\SD_82-VS-SD_59_Cor_DEPs.htm#gene65#gene65) | 1 (0.59%) | ko03060 |
| 66 | [2-Oxocarboxylic acid metabolism](file:///E:\\%E7%8E%8B%E9%9B%AA%E5%B3%B0\\iTRAQ\\%E5%8D%8E%E5%A4%A7%E5%9F%BA%E5%9B%A0\\%E5%8D%8E%E5%A4%A7%E6%95%B0%E6%8D%AE\\%E8%9B%8B%E7%99%BD%E8%B4%A8%E7%BB%84%E4%B8%8E%E8%BD%AC%E5%BD%95%E7%BB%84%E5%85%B3%E8%81%94%E5%88%86%E6%9E%90\\F15FTSNCKF3616\\F15FTSNCKF3616\\Correlation\\SubCorrelation2Function\\Pathway\\SD_82-VS-SD_59_Cor_DEPs_Pathway\\SD_82-VS-SD_59_Cor_DEPs.htm#gene66#gene66) | 1 (0.59%) | ko01210 |
| 67 | [Glycosaminoglycan degradation](file:///E:\\%E7%8E%8B%E9%9B%AA%E5%B3%B0\\iTRAQ\\%E5%8D%8E%E5%A4%A7%E5%9F%BA%E5%9B%A0\\%E5%8D%8E%E5%A4%A7%E6%95%B0%E6%8D%AE\\%E8%9B%8B%E7%99%BD%E8%B4%A8%E7%BB%84%E4%B8%8E%E8%BD%AC%E5%BD%95%E7%BB%84%E5%85%B3%E8%81%94%E5%88%86%E6%9E%90\\F15FTSNCKF3616\\F15FTSNCKF3616\\Correlation\\SubCorrelation2Function\\Pathway\\SD_82-VS-SD_59_Cor_DEPs_Pathway\\SD_82-VS-SD_59_Cor_DEPs.htm#gene67#gene67) | 1 (0.59%) | ko00531 |
| 68 | [Photosynthesis - antenna proteins](file:///E:\\%E7%8E%8B%E9%9B%AA%E5%B3%B0\\iTRAQ\\%E5%8D%8E%E5%A4%A7%E5%9F%BA%E5%9B%A0\\%E5%8D%8E%E5%A4%A7%E6%95%B0%E6%8D%AE\\%E8%9B%8B%E7%99%BD%E8%B4%A8%E7%BB%84%E4%B8%8E%E8%BD%AC%E5%BD%95%E7%BB%84%E5%85%B3%E8%81%94%E5%88%86%E6%9E%90\\F15FTSNCKF3616\\F15FTSNCKF3616\\Correlation\\SubCorrelation2Function\\Pathway\\SD_82-VS-SD_59_Cor_DEPs_Pathway\\SD_82-VS-SD_59_Cor_DEPs.htm#gene68#gene68) | 1 (0.59%) | ko00196 |
| 69 | [Biosynthesis of unsaturated fatty acids](file:///E:\\%E7%8E%8B%E9%9B%AA%E5%B3%B0\\iTRAQ\\%E5%8D%8E%E5%A4%A7%E5%9F%BA%E5%9B%A0\\%E5%8D%8E%E5%A4%A7%E6%95%B0%E6%8D%AE\\%E8%9B%8B%E7%99%BD%E8%B4%A8%E7%BB%84%E4%B8%8E%E8%BD%AC%E5%BD%95%E7%BB%84%E5%85%B3%E8%81%94%E5%88%86%E6%9E%90\\F15FTSNCKF3616\\F15FTSNCKF3616\\Correlation\\SubCorrelation2Function\\Pathway\\SD_82-VS-SD_59_Cor_DEPs_Pathway\\SD_82-VS-SD_59_Cor_DEPs.htm#gene69#gene69) | 1 (0.59%) | ko01040 |
| 70 | [Folate biosynthesis](file:///E:\\%E7%8E%8B%E9%9B%AA%E5%B3%B0\\iTRAQ\\%E5%8D%8E%E5%A4%A7%E5%9F%BA%E5%9B%A0\\%E5%8D%8E%E5%A4%A7%E6%95%B0%E6%8D%AE\\%E8%9B%8B%E7%99%BD%E8%B4%A8%E7%BB%84%E4%B8%8E%E8%BD%AC%E5%BD%95%E7%BB%84%E5%85%B3%E8%81%94%E5%88%86%E6%9E%90\\F15FTSNCKF3616\\F15FTSNCKF3616\\Correlation\\SubCorrelation2Function\\Pathway\\SD_82-VS-SD_59_Cor_DEPs_Pathway\\SD_82-VS-SD_59_Cor_DEPs.htm#gene70#gene70) | 1 (0.59%) | ko00790 |
| 71 | [Thiamine metabolism](file:///E:\\%E7%8E%8B%E9%9B%AA%E5%B3%B0\\iTRAQ\\%E5%8D%8E%E5%A4%A7%E5%9F%BA%E5%9B%A0\\%E5%8D%8E%E5%A4%A7%E6%95%B0%E6%8D%AE\\%E8%9B%8B%E7%99%BD%E8%B4%A8%E7%BB%84%E4%B8%8E%E8%BD%AC%E5%BD%95%E7%BB%84%E5%85%B3%E8%81%94%E5%88%86%E6%9E%90\\F15FTSNCKF3616\\F15FTSNCKF3616\\Correlation\\SubCorrelation2Function\\Pathway\\SD_82-VS-SD_59_Cor_DEPs_Pathway\\SD_82-VS-SD_59_Cor_DEPs.htm#gene71#gene71) | 1 (0.59%) | ko00730 |
| 72 | [Arginine and proline metabolism](file:///E:\\%E7%8E%8B%E9%9B%AA%E5%B3%B0\\iTRAQ\\%E5%8D%8E%E5%A4%A7%E5%9F%BA%E5%9B%A0\\%E5%8D%8E%E5%A4%A7%E6%95%B0%E6%8D%AE\\%E8%9B%8B%E7%99%BD%E8%B4%A8%E7%BB%84%E4%B8%8E%E8%BD%AC%E5%BD%95%E7%BB%84%E5%85%B3%E8%81%94%E5%88%86%E6%9E%90\\F15FTSNCKF3616\\F15FTSNCKF3616\\Correlation\\SubCorrelation2Function\\Pathway\\SD_82-VS-SD_59_Cor_DEPs_Pathway\\SD_82-VS-SD_59_Cor_DEPs.htm#gene72#gene72) | 1 (0.59%) | ko00330 |
| 73 | [Pyrimidine metabolism](file:///E:\\%E7%8E%8B%E9%9B%AA%E5%B3%B0\\iTRAQ\\%E5%8D%8E%E5%A4%A7%E5%9F%BA%E5%9B%A0\\%E5%8D%8E%E5%A4%A7%E6%95%B0%E6%8D%AE\\%E8%9B%8B%E7%99%BD%E8%B4%A8%E7%BB%84%E4%B8%8E%E8%BD%AC%E5%BD%95%E7%BB%84%E5%85%B3%E8%81%94%E5%88%86%E6%9E%90\\F15FTSNCKF3616\\F15FTSNCKF3616\\Correlation\\SubCorrelation2Function\\Pathway\\SD_82-VS-SD_59_Cor_DEPs_Pathway\\SD_82-VS-SD_59_Cor_DEPs.htm#gene73#gene73) | 1 (0.59%) | ko00240 |
| 74 | [Steroid biosynthesis](file:///E:\\%E7%8E%8B%E9%9B%AA%E5%B3%B0\\iTRAQ\\%E5%8D%8E%E5%A4%A7%E5%9F%BA%E5%9B%A0\\%E5%8D%8E%E5%A4%A7%E6%95%B0%E6%8D%AE\\%E8%9B%8B%E7%99%BD%E8%B4%A8%E7%BB%84%E4%B8%8E%E8%BD%AC%E5%BD%95%E7%BB%84%E5%85%B3%E8%81%94%E5%88%86%E6%9E%90\\F15FTSNCKF3616\\F15FTSNCKF3616\\Correlation\\SubCorrelation2Function\\Pathway\\SD_82-VS-SD_59_Cor_DEPs_Pathway\\SD_82-VS-SD_59_Cor_DEPs.htm#gene74#gene74) | 1 (0.59%) | ko00100 |
| 75 | [Cutin, suberine and wax biosynthesis](file:///E:\\%E7%8E%8B%E9%9B%AA%E5%B3%B0\\iTRAQ\\%E5%8D%8E%E5%A4%A7%E5%9F%BA%E5%9B%A0\\%E5%8D%8E%E5%A4%A7%E6%95%B0%E6%8D%AE\\%E8%9B%8B%E7%99%BD%E8%B4%A8%E7%BB%84%E4%B8%8E%E8%BD%AC%E5%BD%95%E7%BB%84%E5%85%B3%E8%81%94%E5%88%86%E6%9E%90\\F15FTSNCKF3616\\F15FTSNCKF3616\\Correlation\\SubCorrelation2Function\\Pathway\\SD_82-VS-SD_59_Cor_DEPs_Pathway\\SD_82-VS-SD_59_Cor_DEPs.htm#gene75#gene75) | 1 (0.59%) | ko00073 |
| 76 | [Carbon fixation in photosynthetic organisms](file:///E:\\%E7%8E%8B%E9%9B%AA%E5%B3%B0\\iTRAQ\\%E5%8D%8E%E5%A4%A7%E5%9F%BA%E5%9B%A0\\%E5%8D%8E%E5%A4%A7%E6%95%B0%E6%8D%AE\\%E8%9B%8B%E7%99%BD%E8%B4%A8%E7%BB%84%E4%B8%8E%E8%BD%AC%E5%BD%95%E7%BB%84%E5%85%B3%E8%81%94%E5%88%86%E6%9E%90\\F15FTSNCKF3616\\F15FTSNCKF3616\\Correlation\\SubCorrelation2Function\\Pathway\\SD_82-VS-SD_59_Cor_DEPs_Pathway\\SD_82-VS-SD_59_Cor_DEPs.htm#gene76#gene76) | 1 (0.59%) | ko00710 |
| 77 | [beta-Alanine metabolism](file:///E:\\%E7%8E%8B%E9%9B%AA%E5%B3%B0\\iTRAQ\\%E5%8D%8E%E5%A4%A7%E5%9F%BA%E5%9B%A0\\%E5%8D%8E%E5%A4%A7%E6%95%B0%E6%8D%AE\\%E8%9B%8B%E7%99%BD%E8%B4%A8%E7%BB%84%E4%B8%8E%E8%BD%AC%E5%BD%95%E7%BB%84%E5%85%B3%E8%81%94%E5%88%86%E6%9E%90\\F15FTSNCKF3616\\F15FTSNCKF3616\\Correlation\\SubCorrelation2Function\\Pathway\\SD_82-VS-SD_59_Cor_DEPs_Pathway\\SD_82-VS-SD_59_Cor_DEPs.htm#gene77#gene77) | 1 (0.59%) | ko00410 |
| 78 | [Vitamin B6 metabolism](file:///E:\\%E7%8E%8B%E9%9B%AA%E5%B3%B0\\iTRAQ\\%E5%8D%8E%E5%A4%A7%E5%9F%BA%E5%9B%A0\\%E5%8D%8E%E5%A4%A7%E6%95%B0%E6%8D%AE\\%E8%9B%8B%E7%99%BD%E8%B4%A8%E7%BB%84%E4%B8%8E%E8%BD%AC%E5%BD%95%E7%BB%84%E5%85%B3%E8%81%94%E5%88%86%E6%9E%90\\F15FTSNCKF3616\\F15FTSNCKF3616\\Correlation\\SubCorrelation2Function\\Pathway\\SD_82-VS-SD_59_Cor_DEPs_Pathway\\SD_82-VS-SD_59_Cor_DEPs.htm#gene78#gene78) | 1 (0.59%) | ko00750 |
| 79 | [Glycosphingolipid biosynthesis - globo series](file:///E:\\%E7%8E%8B%E9%9B%AA%E5%B3%B0\\iTRAQ\\%E5%8D%8E%E5%A4%A7%E5%9F%BA%E5%9B%A0\\%E5%8D%8E%E5%A4%A7%E6%95%B0%E6%8D%AE\\%E8%9B%8B%E7%99%BD%E8%B4%A8%E7%BB%84%E4%B8%8E%E8%BD%AC%E5%BD%95%E7%BB%84%E5%85%B3%E8%81%94%E5%88%86%E6%9E%90\\F15FTSNCKF3616\\F15FTSNCKF3616\\Correlation\\SubCorrelation2Function\\Pathway\\SD_82-VS-SD_59_Cor_DEPs_Pathway\\SD_82-VS-SD_59_Cor_DEPs.htm#gene79#gene79) | 1 (0.59%) | ko00603 |

**Supplementary Table 4**. List of cor-DEGs-DEPs enriched in both transcriptome and proteome in BS82-VS-BS59, SD82-VS-SD59, and SD82-VS-BS82 groups.

**BS82-VS-BS59**

| Gene name | Correlations | NCBInr Description |
| --- | --- | --- |
| Bv6_153260_pdmn.t1 | (Protein:-0.599\|- @@Gene:-2.502\|-); | ribonuclease 1-like |
| Bv9_207310_uxns.t1 | (Protein:-0.269\|-@@Gene:1.069\|+); | DEAD-box ATP-dependent RNA helicase |
| Bv3_060640_zokm.t1 | (Protein:0.367\|+@@Gene:2.301\|+); | TolB protein-like protein |
| Bv3_053900_qhgs.t1 | (Protein:0.333\|+@@Gene:2.700\|+); | heat shock protein 83-like |
| Bv1_003130_spiz.t1 | (Protein:-0.377\|- @@Gene:-1.881\|-); | aspartyl protease family protein 2 |
| Bv8u_204710_otoo.t1 | (Protein:-0.690\|- @@Gene:-2.061\|-) | Xyloglucan endotransglucosylase/hydrolase protein 8 |
| Bv4u_091190_ygqi.t1 | (Protein:-0.396\|- @@Gene:-1.234\|-); | 20 kDa chaperonin, chloroplastic |
| Bv2_035530_ffcc.t1 | (Protein:-0.218\|- @@Gene:-4.331\|-); | hypothetical protein PRUPE_ppa006179mg |
| Bv7_172260_jeep.t1 | (Protein:-0.667\|- @@Gene:-3.627\|-); | pectinesterase 3-like |
| Bv_43200_scgp.t1 | (Protein:0.791\|+ @@Gene:-1.879\|-); | Periplasmic beta-glucosidase precursor |

**SD82-VS-SD59**

| Gene name | Correlations | NCBInr Description |
| --- | --- | --- |
| Bv6_153260_pdmn.t1 | (Protein:-0.599\|- @@Gene:-2.502\|-); | ribonuclease 1-like |
| Bv9_207310_uxns.t1 | (Protein:-0.184\|- @@Gene:1.559\|+) | DEAD-box ATP-dependent RNA helicase |
| Bv3_060640_zokm.t1 | (Protein:0.651\|+ @@Gene:2.046\|+); | TolB protein-like protein |
| Bv3_053900_qhgs.t1 | (Protein:0.516\|+ @@Gene:2.629\|+); | heat shock protein 83-like |
| Bv1_003130_spiz.t1 | (Protein:-0.377\|- @@Gene:-3.104\|-); | aspartyl protease family protein 2 |
| Bv8u_204710_otoo.t1 | (Protein:-0.535\|- @@Gene:-1.084\|-) | xyloglucan endotransglucosylase/hydrolase protein 8 |
| Bv4u_091190_ygqi.t1 | (Protein:-0.474\|- @@Gene:-1.244\|-); | 20 kDa chaperonin, chloroplastic |
| Bv2_043900_thoh.t1 | (Protein:-0.434\|- @@Gene:-3.731\|-); | aspartyl protease AED3 |
| Bv_38810_ipip.t1 | (Protein:-0.690\|- @@Gene:-2.971\|-); | endoglucanase 2 |
| Bv3_053660_hmht.t1 | (Protein:1.778\|+ @@Gene:5.408\|+); | alpha-glucosidase |
| Bv5_094840_upzj.t1 | (Protein:-0.494\|- @@Gene:-4.653\|-); | probable polygalacturonase |
| Bv1_008140_uzgx.t1 | (Protein:1.561\|+ @@Gene:5.284\|+); | basic endochitinase |
| Bv8_202140_kacq.t1 | (Protein:1.570\|+ @@Gene:4.795\|+); | acidic mammalian chitinase |
| Bv9_211960_arex.t1 | (Protein:-0.515\|- @@Gene:-3.298\|-); | ribosome-inactivating protein lychnin |
| Bv9_206800_xxdr.t1 | (Protein:0.918\|+ @@Gene:3.380\|+); | antiviral protein alpha isoform X2 |
| Bv5_107300_inqt.t1 | (Protein:0.585\|+ @@Gene:1.145\|+) | AMP-activated protein kinase, gamma regulatory subunit |
| Bv1_003710_nmtd.t1 | (Protein:-0.304\|- @@Gene:-1.202\|-) | tubulin gamma-2 chain-like |
| Bv4_081320_mxfp.t1 | (Protein:-0.269\|- @@Gene:-1.206\|-) | probable UDP-arabinopyranose mutase 5 |
| Bv1_010170_rznm.t1 | (Protein:-0.599\|- @@Gene:-3.906\|-) | Periplasmic beta-glucosidase precursor |
| Bv4_074260_fchq.t1 | (Protein:-0.322\|- @@Gene:-1.292\|-) | tubulin, beta chain |
| Bv1u_019010_zfif.t1 | (Protein:-0.201\|- @@Gene:-2.111\|-) | beta-tubulin |
| Bv2_033880_rqef.t1 | (Protein:-0.621\|- @@Gene:-2.382\|-) | Sal k 4 pollen allergen |
| Bv6_134170_tjat.t1 | (Protein:-0.269\|- @@Gene:-1.847\|-) | tubulin alpha-1 chain-like |
| Bv_07900_yyiy.t1 | (Protein:-0.396\|- @@Gene:-3.462\|-) | Histone H2B |
| Bv6_126570_nmnx.t1 | (Protein:0.941\|+ @@Gene:1.730\|+) | putative linker histone H1 variant protein |
| Bv4_073360_qhcc.t1 | (Protein:1.367\|+ @@Gene:2.960\|+) | glucan endo-1,3-beta-D-glucosidase |
| Bv6_141340_gsxm.t1 | (Protein:-1.599\|- @@Gene:-5.635\|-) | hypothetical protein PRUPE_ppa004172mg |
| Bv3_053940_tysr.t1 | (Protein:-0.269\|- @@Gene:-3.262\|-) | polygalacturonase |
| Bv6u_155430_hanj.t1 | (Protein:2.370\|+ @@Gene:4.531\|+) | Lichenase precursor, putative |
| Bv4_081830_wknz.t1 | (Protein:0.275\|+ @@Gene:-1.211\|-) | hypothetical protein VITISV_002311 |
| Bv3_066810_jhcc.t1 | (Protein:-0.644\|- @@Gene:-4.397\|-) | endo-1,4-beta-xylanase A-like |
| Bv5_093820_tqes.t1 | (Protein:-0.713\|- @@Gene:-3.864\|-) | endo-1,4-beta-glucanase |

**SD82-VS-BS82**

| Gene name | Correlations | NCBInr Description |
| --- | --- | --- |
| Bv6_153260_pdmn.t1 | (Protein:-0.599\|- @@Gene:-2.502\|-); | ribonuclease 1-like |
| Bv2_043900_thoh.t1 | (Protein:-0.474\|- @@Gene:-2.115\|-) | aspartyl protease AED3 |
| Bv_38810_ipip.t1 | (Protein:-0.737\|- @@Gene:-1.943\|-) | endoglucanase 2 |
| Bv3_053660_hmht.t1 | (Protein:1.000\|+ @@Gene:-2.579\|-) | alpha-glucosidase |
| Bv5_094840_upzj.t1 | (Protein:-0.377\|- @@Gene:-2.678\|-) | probable polygalacturonase |
| Bv1_008140_uzgx.t1 | (Protein:2.722\|+ @@Gene:2.402\|+) | basic endochitinase |
| Bv8_202140_kacq.t1 | (Protein:1.637\|+ @@Gene:4.403\|+) | acidic mammalian chitinase |
| Bv9_211960_arex.t1 | (Protein:-0.916\|- @@Gene:-2.587\|-) | ribosome-inactivating protein lychnin |
| Bv9_206800_xxdr.t1 | (Protein:0.791\|+ @@Gene:1.338\|+) | antiviral protein alpha isoform X2 |
| Bv_51330_psrc.t1 | (Protein:0.585\|+ @@Gene:1.110\|+) | xyloglucan endotransglycosylase/hydrolase 1 |
| Bv5_118870_tmyg.t1 | (Protein:0.791\|+ @@Gene:1.248\|+) | proteinase inhibitor |
| Bv6_125340_epqo.t1 | (Protein:-0.713\|- @@Gene:-2.349\|-) | germin-like protein 2 |
| Bv6_129620_ycwx.t1 | (Protein:-0.152\|- @@Gene:-1.156\|-) | pectinesterase/pectinesterase inhibitor 51 |
| Bv3_049080_cdxa.t1 | (Protein:0.345\|+ @@Gene:1.317\|+) | cyanate hydratase |
| Bv_29360_ynpx.t1 | (Protein:-0.515\|- @@Gene:-1.031\|-) | reticuline oxidase-like protein |
| Bv3_054260_pgry.t1 | (Protein:-0.415\|- @@Gene:-1.184\|-) | medium-chain-fatty-acid--CoA ligase |
| Bv4_071340_dotf.t1 | (Protein:-0.358\|- @@Gene:1.108\|+) | uridylate kinase plant, putative |
| Bv7_177220_cmin.t1 | (Protein:-0.737\|- @@Gene:-1.680\|-) | glucan endo-1,3-beta-glucosidase precursor |
| Bv4_071930_gjzi.t1 | (Protein:-0.286\|- @@Gene:-1.481\|-) | predicted protein |
| Bv7_168180_wfoe.t1 | (Protein:0.454\|+ @@Gene:-1.871\|-) | tyrosine decarboxylase 1 |
| Bv6_131340_pijf.t1 | (Protein:0.454\|+ @@Gene:1.608\|+) | L-ascorbate oxidase homolog |
| Bv1_006110_ctqz.t1 | (Protein:0.856\|+ @@Gene:2.867\|+) | hypothetical protein PRUPE_ppa015667mg |
| Bv8_196990_khdk.t1 | (Protein:-0.234\|- @@Gene:-3.223\|-) | predicted protein |
| Bv6_153060_nwzh.t1 | (Protein:-0.556\|- @@Gene:-4.302\|-) | GTP cyclohydrolase I |
| Bv7_160760_mmcw.t1 | (Protein:0.614\|+ @@Gene:1.105\|+) | caffeic acid 3-O-methyltransferase |
| Bv7_166290_gyqi.t1 | (Protein:-0.358\|- @@Gene:-1.443\|-) | putative UDP-glucuronate decarboxylase 3 |
| Bv9_214480_chaa.t1 | (Protein:0.575\|+ @@Gene:2.127\|+) | acyl-coenzyme A oxidase 3 |
| Bv2_035180_puci.t1 | (Protein:-0.667\|- @@Gene:-2.080\|-) | Lysine decarboxylase-like protein |
| Bv9_212920_hdnw.t1 | (Protein:-0.304\|- @@Gene:-1.711\|-) | beta-galactosidase, putative |
| Bv_39990_qane.t1 | (Protein:0.251\|+ @@Gene:1.089\|+) | branched-chain-amino-acid aminotransferase-like protein 2 |
| Bv6_154620_umck.t1 | (Protein:0.678\|+ @@Gene:1.871\|+) | UDP-glycosyltransferase 79B6-like |
| Bv1_014150_utfq.t1 | (Protein:-0.340\|- @@Gene:-1.390\|-) | protein with unknown function |
| Bv_18470_twpd.t1 | (Protein:0.816\|+ @@Gene:3.067\|+) | GDSL esterase/lipase At3g26430-like |
| Bv8_197720_uyjt.t1 | (Protein:-0.415\|- @@Gene:-2.263\|-) | heparanase-like protein 3-like |
| Bv1_013750_hurs.t1 | (Protein:0.722\|+ @@Gene:2.150\|+) | peroxidase |
| Bv1_009180_jmpr.t1 | (Protein:-0.358\|- @@Gene:-1.823\|-) | phospho-2-dehydro-3-deoxyheptonate aldolase 2 |
| Bv5_099790_mkdg.t1 | (Protein:0.714\|+ @@Gene:1.953\|+) | oxidoreductase, putative |
| Bv5_112700_mohc.t1 | (Protein:0.848\|+ @@Gene:-1.482\|-) | chitinase |
| Bv7_170690_ymgq.t1 | (Protein:-0.340\|- @@Gene:-1.146\|-) | cytochrome b5 |
| Bv_13960_xghz.t1 | (Protein:1.915\|+ @@Gene:3.100\|+) | L-ascorbate peroxidase 2, cytosolic-like |
| Bv3_058520_cotf.t1 | (Protein:-0.535\|- @@Gene:-2.923\|-) | cytosolic sulfotransferase 1 |
| Bv8_197080_yast.t1 | (Protein:-0.737\|- @@Gene:-3.485\|-) | reticuline oxidase precursor, putative |
| Bv9_226790_cjsi.t1 | (Protein:-0.304\|- @@Gene:-1.138\|-) | probable LRR receptor-like serine/threonine protein kinase At4g20940-like isoform 1 |
| Bv8_202760_pqfr.t1 | (Protein:-0.340\|- @@Gene:-1.259\|-) | plasma membrane H+-ATPase |
| Bv4_089870_qooo.t1 | (Protein:-0.136\|- @@Gene:-1.129\|-) | farnesyl pyrophosphate synthase |
| Bv5_096070_yisx.t1 | (Protein:-0.252\|- @@Gene:-2.387\|-) | hydroxymethylglutaryl-CoA synthase |
| Bv8_194340_nxyi.t1 | (Protein:-0.474\|- @@Gene:-2.164\|-) | beta-glucosidase, putative |
| Bv_14340_hneu.t2 | (Protein:-0.322\|- @@Gene:-1.079\|-) | phosphoglucan phosphatase DSP4 |
| Bv7_175830_xykn.t1 | (Protein:-0.535\|- @@Gene:-1.875\|-) | 60S acidic ribosomal protein P0-1-like |
| Bv_18410_tznx.t1 | (Protein:0.390\|+ @@Gene:-1.081\|-) | GDSL esterase/lipase At3g26430-like isoform 1 |
| Bv3_057720_dtna.t1 | (Protein:-0.377\|- @@Gene:-1.875\|-) | cellulose synthase-like protein G1-like |
| Bv5_112390_zxur.t1 | (Protein:-0.454\|- @@Gene:-1.228\|-) | heparanase-2, putative |
| Bv7_158580_joiu.t1 | (Protein:-0.201\|- @@Gene:-1.206\|-) | hypothetical protein PRUPE_ppa004903mg |
| Bv_50640_zudu.t1 | (Protein:-0.667\|- @@Gene:-1.891\|-) | petal death protein |
| Bv5_111820_ofgq.t1 | (Protein:0.356\|+ @@Gene:1.264\|+) | isoaspartyl peptidase/L-asparaginase 1 |
| Bv2_034870_ygce.t1 | (Protein:-0.340\|- @@Gene:-1.851\|-) | hypothetical protein PRUPE_ppa005426mg |
| Bv8_185350_gwqc.t1 | (Protein:0.824\|+ @@Gene:1.449\|+) | epoxide hydrolase, putative |
| Bv2_039920_zdog.t1 | (Protein:-1.089\|- @@Gene:-1.553\|-) | probable inactive receptor kinase At5g58300 |
| Bv5_113980_zuju.t1 | (Protein:-0.621\|- @@Gene:-1.201\|-) | monosaccharide-sensing protein 2 |
| Bv9_219010_mfea.t1 | (Protein:0.322\|+ @@Gene:-1.215\|-) | L-ascorbate oxidase homolog |
| Bv8_203060_pggz.t1 | (Protein:-0.286\|- @@Gene:-1.844\|-) | 4-coumarate:coenzyme A ligase |
| Bv6_130670_hywz.t1 | (Protein:-1.184\|- @@Gene:-4.127\|-) | probable mannitol dehydrogenase |
| Bv2_039770_ariz.t1 | (Protein:-0.415\|- @@Gene:-1.164\|-) | cytochrome P450 734A1 |
| Bv3_065790_cwxc.t1 | (Protein:-0.234\|- @@Gene:-1.391\|-) | hypothetical protein PRUPE_ppa004404mg |
| Bv5_103630_oxnj.t1 | (Protein:1.070\|+ @@Gene:2.873\|+) | similar to isoflavone reductase homolog |
| Bv7u_181660_sqfu.t1 | (Protein:-0.474\|- @@Gene:-1.312\|-) | cytochrome P450, putative |
| Bv4_081710_rtpk.t1 | (Protein:-0.621\|- @@Gene:-1.998\|-) | probable linoleate 9S-lipoxygenase 5 |
| Bv_43370_gpmg.t1 | (Protein:-0.218\|- @@Gene:-1.699\|-) | hypothetical protein VITISV_015121 |
